# Supplementary material for: Developing a core outcome set for interventions to improve discharge from mental health inpatient services: a survey, Delphi and consensus meeting with key stakeholder groups
Source: BMJ Open. 2020 May 12;10(5):e034215. doi: 10.1136/bmjopen-2019-034215 (PMC7228512; doi:10.1136/bmjopen-2019-034215)
Supplement: Supplementary data [file bmjopen-2019-034215supp001.pdf]

Supplementary Material

File 1: Table of questions asked in qualitative questionnaire

Group	Question
All Participants	What would make discharge from an acute mental health ward **safe** in your opinion?
All Participants	What would make discharge from an acute mental health ward **effective** in your opinion?
All Participants	What would you like to see prioritised for research into discharge from an acute ward?
All Participants	Can you think of any important outcomes to measure in research assessing discharge interventions?
Service User	What do you think is the most difficult aspect of discharge from a mental health acute ward?
Service User	What might improve discharge from a mental health ward?
Service User	Was there any one person/group/intervention which really made a difference for you? If so what was it? What made it so powerful?
Service User	Do you have any other feelings or concerns not addressed in previous questions?
Family member/carer	What do you consider to be the most difficult components of discharge for your family member/friend from an acute mental health ward?
Family member/carer	What types of improvement would you expect to see in your family member/friend following a successful discharge from an acute mental health ward?
Mental health professional	What do you consider to be the most difficult aspects of discharge from an acute unit for your service-users?
Mental health professional	Are there any changes/behaviours you would expect to see in a service-user during or following a safe and/or effective discharge?
Researchers	If applicable, what outcomes did you measure in past research (of discharge interventions)?
Researchers	Are there any concepts that you think are important to measure, but chose not to, due to not having a suitable measurement instrument?
End users of research	If you were looking to use research to inform changes to the discharge procedure within your professional role, what outcomes would you like to see reported?
End users of research	In your opinion, what measurements would persuade you that a discharge intervention is effective?

File 2: Results of Meta-synthesis of Outcomes and data from questionnaire

Clinical

From Review	Additional from Meta-synthesis
Reoccurrence (i.e. Relapse)	Stability
Clinical Recovery	Physical Health and Wellbeing
Personal Recovery	Iatrogenic harm (patient safety incidents- harm caused by the care system)
Substance Misuse (inc. alcohol)	Serious accidental injury
Self-injury (i.e. harm to self)	Mortality
Suicide Risk	Mental health and illness (symptoms/psychological distress)
Suicidal Ideation	
Suicide Attempted	
Suicide Completed	
Medication Knowledge	
Medication Adherence	
Medication Side Effects	
Medication Management	
Global Functioning	

Service

From Review	Additional from Meta-synthesis
Emergency department visits post discharge	Police intervention post discharge
Readmission	Engagement with psychological intervention
Length of stay pre-discharge	Engagement with community services
Length of stay post-discharge (i.e. subsequent readmissions)	Availability of appropriate community support (i.e. allocated key worker)
Outpatient appointment/visit adherence	Length of time before follow up/aftercare
Service use	Leaving the hospital against medical advice
	Service user involvement in decision making (shared decision making)
	Serious incidents (i.e. serious incidents reported formally)

Satisfaction

From Review	Additional from Meta-synthesis
Service user expectations of care	Professionals/care teams satisfaction with information provision at discharge
Service user satisfaction with discharge	Service user satisfaction with information provision at discharge (e.g. regarding medication, risk, crisis planning)
Service user experience of discharge	
Service user satisfaction with treatment	
Carer/family/other satisfaction with discharge	
Staff satisfaction with discharge	

Personal

From Review	Additional from Meta-synthesis
Hopelessness	Feelings of safety
Boredom	Feelings of support (from various groups- i.e. family, professionals and friends)
Loneliness	Experience of stigma
Isolation	Quality of interpersonal relationships (friends and family)
Coping skills	Self-management
Concern/anxiety about discharge	
Autonomy (e.g. independence, autonomous decision making)	

Housing Stability	
Discharge to appropriate accommodation	
Service user knowledge of own condition	
Quality of life	
Community participation	
Violence	
Aggression	
Victim of crime	
Stable relationships with health professionals	
Employment	

Discharge Planning

From Review	Additional from Meta-synthesis
	Service user involvement in discharge planning
	Family/carer involvement in discharge planning
	Advocate involvement in discharge planning
	Completion of planned care
	Continuity of contact
	Service user readiness for discharge (incl. preparation)
	Clinical readiness for discharge
	Experience of coercion at discharge (e.g. feeling forced to leave)
	Provision of financial support (e.g. benefits advice, debt advice)
	Delays in expected discharge
	Service availability (e.g. availability of community services, CPN, follow-up, social worker, bed in residential service)
	Family/carer/other readiness for discharge
	Staff understanding of safe and effective discharge
	Plan for community engagement
	Is there a plan for care post discharge created
	Has the plan for care post-discharge been communicated with patient
	Has information about the discharge been reported to other relevant services

Changes/Combinations

Outcomes from Review	Changes/Combinations
Social Recovery	Combined into quality of interpersonal relationships
Relapse	PPI suggestion change to reoccurrence
Self-harm	PPI suggestion change to self-injury
Depression	Combined into symptoms
Anxiety	Combined into symptoms
Illegal drug use	Combined into substance misuse
Alochol use	Combined into substance misuse
Addiction severity	Combined into substance misuse
Crisis planning	Combined into information provision
Risk communication	Combined into information provision
Allocated worker	Combined into availability of appropriate community support
Better knowledge transfer	Combined into information provision (professional)
Therapeutic alliance	Combined into stable relationships with HPs
Contact with ambulatory care	Combined into emergency visits
Treatment adherence	Combined into medication adherence and engagement with psychological services
7 day follow up	Changed to length of time before follow up for global audience
Psychological distress	Combined into symptoms
Family relations	Combined into quality of interpersonal relationships
Community integration and functioning	Combined into Community participation

Outcomes Removed from Process

Outcomes from Review	Reason
Antipsychotic Politherapy	Too specific
Autistic life	Too specific

Medication Problems	Too vague
Functional Recovery	Meaning not explicit
Satisfaction with service in community	Not relevant for this COS
Global satisfaction with discharge	Meaning not explicit
Quality of care	Too vague
Treatment decision making	Not relevant for this COS
Financial cost to provider	Impossible to measure
Number of beds	Too vague
Staff workload	Too vague
Freedom	Too vague
Self-activity	Too vague

Carer outcomes

**Outcomes from Review**	**Decision**
Caregiver Burden	Yes
Caregiver Health Status	No (too specific)
Caregiver Knowledge about illness	Yes
**From Synthesis**	
Carer support	No
Relationship between family and SU	No (probably a variable)

File 3: Participant Demographics

**Categories**											
**Group**	**Service Users**	**Families and Carers**	**Healthcare Professionals**	**Researchers**	**End-users of Research**						
**n**	27	17	39	37	15						
**Gender**	**Male**	**Female**	**Other**								
**n**	28	63	0								
**Age**	**18-24**	**25-34**	**35-44**	**45-54**	**55-64**	**65-74**	**75-84**				
**n**	4	20	30	24	10	4	1				
**Location**	**International**	**East of England**	**East Midlands**	**London**	**North West**	**North East**	**South East**	**South West**	**Wales**	**West Midlands**	**Yorkshire and the Humber**
**n**	24	2	11	7	16	1	6	5	0	13	7

Locations for International participants

**Country**	**N**
Australia	4
Canada	2
China	1
France	1
Germany	1
Iran	1
Italy	1
Northern Ireland	1
South Africa	1
Switzerland	4
USA	7

File 4: Full list of Delphi outcomes, consensus levels, and round of inclusion/exclusion

	R1 Percentage agreement	R1 Percentage Disagreement	Median	Researchers	Sus and carers	HCPs and DMs	Round 2 Status	Round 2	Researcher	Sus and carers	HCP and DMs	Median
Service user involvement in discharge planning (inc. feeling listened to)	87%	4%	7	65%	100%	95%	Include					
Functioning (health, social, etc.)	83%	3%	6	69%	100%	81%	Include					
Mental health and illness (symptom/psychological distress)	83%	3%	6	73%	91%	86%	Include					
Personal Recovery	82%	1%	6	75%	86%	86%	Include					
service user understanding of discharge plan	81%	3%	6	65%	91%	86%	Include					
Quality of life	81%	1%	6.5	65%	90%	86%	Include					
suicide Completed	80%	4%	7	80%	90%	68%	Include					
Readmission	80%	6%	6	77%	77%	86%	Include					
service user involvement in decision making (shared decision making)	77%	4%	7	50%	95%	86%	Include					
service user satisfaction with information provision at discharge (e.g. regarding medication, risk, crisis planning)	77%	6%	6	65%	86%	81%	Include					
service user knowledge of how to access community support (i.e. in an emergency)	77%	3%	6	58%	91%	86%	Include					
Recurrence (i.e. relapse)	75%	1%	6	58%	91%	76%	Include					
Suicide Attempted	75%	4%	6	62%	86%	81%	Include					
discharge to appropriate accommodation	75%	3%	6	69%	91%	67%	Include					
service user satisfaction with treatment	74%	7%	6	58%	91%	71%	Re-present	59%	43%	67%	70%	6
service Availability (e.g. availability of community service, CPN, follow-up, social worker,	74%	7%	6	46%	86%	95%	Re-present	45%	27%	67%	50%	5

bed in residential service)												
Existence of a plan for community engagement/ care post discharge	74%	4%	6	58%	77%	90%	Re-present	72%	57%	83%	90%	6
Feeling of support (from various group i.e. family, professional, friends)	72%	4%	6	65%	77%	71%	Re-present	43%	33%	50%	50%	5
Suicide Risk	71%	3%	6	58%	77%	81%	Re-present	42%	43%	56%	25%	5
Family/carer/other involvement in discharge planning (inc. feeling listened too)	71%	6%	6	65%	77%	75%	Re-present	58%	50%	67%	65%	6
service user readiness for discharge (incl. preparation)	71%	6%	6	58%	77%	81%	Re-present	35%	27%	50%	30%	5
Feeling of safety	70%	7%	6	65%	73%	71%	Re-present	36%	37%	56%	25%	5
service user satisfaction with discharge	69%	4%	6	50%	76%	86%	Re-present	45%	40%	50%	50%	5
Physical Health and Wellbeing	68%	3%	6	50%	77%	81%	Re-present	49%	43%	61%	45%	5
service user experience of discharge	68%	10%	6	46%	86%	76%	Re-present	29%	23%	44%	30%	5
Housing stability	68%	3%	6	62%	77%	67%	Re-present	41%	37%	39%	40%	5
Coping skills	68%	6%	6	50%	77%	80%	Re-present	39%	40%	50%	35%	5
Continuity of contact	68%	6%	6	64%	77%	62%	Re-present	65%	63%	72%	60%	6
Mortality	67%	7%	6	68%	76%	57%	Re-present	42%	43%	50%	30%	5
self management	67%	4%	6	46%	86%	71%	Re-present	29%	23%	39%	20%	5
Length of time before follow up/aftercare	65%	6%	6	46%	77%	76%	Re-present	58%	43%	83%	65%	6
Isolation	65%	9%	6	46%	73%	81%	Re-present	55%	50%	72%	45%	6
Availability of appropriate community support (i.e. allocated key worker)	64%	9%	6	38%	82%	76%	Re-present	52%	27%	72%	70%	6
service user knowledge of own condition	64%	6%	6	42%	91%	62%	Re-present	36%	30%	50%	35%	4
service user expectation of care	63%	3%	6	35%	76%	81%	Re-present	28%	20%	28%	40%	4
Hopelessness	63%	9%	6	54%	67%	71%	Re-present	46%	47%	44%	45%	5
suicidal Ideation	62%	7%	6	46%	77%	67%	Re-present	51%	50%	61%	45%	6

Medication Adherence	62%	4%	6	50%	82%	52%	Re-present	45%	47%	44%	40%	5
Loneliness	62%	10%	6	42%	68%	81%	Re-present	41%	37%	56%	30%	5
Information about the discharge reported to other relevant service	62%	9%	6	50%	73%	71%	Re-present	48%	37%	78%	40%	5
service user experience of coercion at discharge	61%	6%	6	46%	77%	63%	Re-present	43%	40%	67%	35%	5
Clinical readiness for discharge	60%	6%	6	48%	67%	67%	Re-present	36%	30%	39%	40%	5
Medication side Effects	59%	7%	6	38%	77%	67%	Re-present	41%	30%	61%	35%	5
Emergency department visit post discharge	59%	6%	6	58%	68%	52%	Re-present	62%	63%	67%	60%	6
Quality of interpersonal relationship (friend and family)	59%	7%	6	50%	77%	52%	Re-present	46%	40%	72%	35%	5
Iatrogenic harm (patient safety incident or harm caused by the care system)	59%	4%	6	40%	64%	71%	Re-present	51%	37%	72%	50%	6
Completion of planned care	59%	7%	6	50%	76%	52%	Re-present	39%	40%	56%	30%	5
Concern/anxiety about discharge	58%	9%	6	31%	73%	76%	Re-present	26%	17%	33%	35%	4
Autonomy (e.g. independence, autonomous decision making)	57%	3%	6	35%	73%	70%	Re-present	36%	27%	50%	30%	5
staff understanding of safe and effective discharge	57%	10%	6	40%	68%	67%	Re-present	41%	30%	67%	35%	4
self injury (i.e. harm to self)	57%	6%	6	50%	64%	52%	Re-present	49%	53%	61%	35%	5
Financial support (e.g. benefit advice, debt advice)	57%	9%	6	38%	82%	57%	Re-present	46%	40%	72%	30%	5
stable relationship with health professional	55%	4%	6	38%	71%	58%	Re-present	33%	37%	50%	20%	5
Medication Management	54%	3%	6	42%	71%	52%	Re-present	42%	37%	61%	40%	5
Carer/family/other satisfaction with discharge	54%	9%	6	35%	68%	57%	Re-present	36%	27%	61%	35%	4
Clinical Recovery	53%	7%	6	60%	55%	43%	Re-present	36%	33%	50%	30%	5
service use	52%	4%	6	46%	50%	63%	Re-present	30%	33%	39%	15%	4
Violence	52%	9%	6	46%	60%	48%	Re-present	29%	33%	33%	15%	4
Victim of crime	52%	12%	6	38%	68%	48%	Re-present	28%	30%	44%	10%	4
Experience of stigma	52%	7%	6	42%	59%	57%	Re-present	30%	20%	50%	25%	4

Engagement with psychological intervention	51%	4%	6	35%	45%	76%	Re-present	33%	30%	67%	5%	4
Cancellation or change to follow up meeting	51%	12%	6	19%	82%	57%	Re-present	42%	23%	67%	40%	5
serious incident (i.e. serious incident reported formally)	50%	6%	5.5	44%	45%	62%	Exclude					
Employment	50%	7%	5.5	58%	48%	38%	Exclude					
Medication Knowledge	49%	9%	5	35%	64%	52%	Exclude					
Engagement with community service	49%	4%	5	35%	50%	71%	Exclude					
Length of stay pre-discharge	46%	10%	5	38%	55%	48%	Exclude					
Family/carer/other readiness for discharge	46%	9%	5	42%	55%	43%	Exclude					
Leaving the hospital against medical advice	45%	14%	5	46%	55%	33%	Exclude					
Outpatient appointment/visit adherence	44%	4%	5	54%	57%	24%	Exclude					
Aggression	44%	10%	5	42%	43%	48%	Exclude					
Delay in expected discharge	43%	13%	5	12%	67%	57%	Exclude					
substance Misuse (incl. alcohol)	41%	9%	5	28%	50%	48%	Exclude					
serious accidental injury	40%	12%	5	25%	36%	62%	Exclude					
Community participation	39%	6%	5	35%	36%	48%	Exclude					
stability	38%	4%	5	13%	59%	47%	Exclude					
Advocate involvement in discharge planning	37%	12%	5	24%	45%	45%	Exclude					
Professional/care team satisfaction with information provision at discharge	36%	13%	5	19%	45%	48%	Exclude					
Primary Care/Community service/Nongovernmental or Charity service satisfaction	35%	16%	5	15%	50%	48%	Exclude					
Police intervention post discharge	34%	16%	5	35%	32%	35%	Exclude					
staff satisfaction with discharge	28%	16%	5	19%	36%	29%	Exclude					
Boredom	26%	25%	4	12%	36%	33%	Exclude					
Meaningful Activity (i.e. employment, studying, volunteering)	New outcome proposed in round 1						Include	77%	73%	83.33%	80%	6
Resilience	New outcome proposed in round 1				Do not present							
Dual harm (i.e. a person harms themselves and others)	New outcome proposed in round 1						Exclude	32%	23%	55.56%	30%	4

Annual admissions	New outcome proposed in round 1	Do not present						
Harm to others (violence, criminal or risk-taking behaviour)	New outcome proposed in round 1	Do not present						
Levels of patient confidentiality	New outcome proposed in round 1	Do not present						
Personal learning	New outcome proposed in round 1	Do not present						

File 5: Proposed potential service user discharge experience PROM that includes items of most importance to stakeholders in addition to the core outcome set

Item	Strongly Agree	Agree	Neither Agree nor Disagree	Disagree	Strongly Disagree
1. I felt involved in my discharge planning					
2. I understood my discharge plan					
3. I know how to access support in the community					
4. I was frequently involved in decisions about my care at discharge					
5. I was discharged to appropriate accommodation					
6. I was satisfied with the information provided to me at discharge					

File 6: Outcome Measure Recommendation Results

Methods

After the core outcome set was agreed in the consensus meeting, we invited all participants from the earlier stages of the project to recommend measures and time markers in a final online questionnaire. Participants were invited to participate if they had been involved in any of the previous online rounds. The invitation made it clear that the questionnaire is most relevant to researchers, but that other groups with an opinion or interest are welcome to contribute. This was due to the specific knowledge of instruments required to complete this round.

In this questionnaire participants were presented with the four core outcomes. For each core outcome they were presented with any measures used to assess that outcome in our systematic review studies [1] and any additional measures that had been recommended to the team during the process. Participants were asked to choose the one most appropriate, (don't know, other, new instrument, no instrument were also options). A second question also asked which time markers would be recommended, with options to select all applicable. These options were also developed based on time markers used in the systematic review [1].

Results

Forty-three of the 93 invited participants responded (15 service users, 8 family members/carers, 23 researchers, 10 healthcare professionals, 3 end users of research), although as in previous rounds these were not distinct categories. Fifty-three percent of the respondents were researchers, this was expected as in the email we suggested that this stage may be more meaningful or of interest to this group, but as a team we chose not to exclude other groups with opinions on measurement instruments. Twenty-three percent of participants were international

researchers (from USA, Switzerland, Canada, and Australia). Table 4 shows the preliminary minimum measure recommendations and time markers, additional file 5 shows the results upon which the recommendations were based.

Table 1: Summary of Measurement Recommendations

Core Outcome	Instrument/Measure	Time Marker
Readmission	Retrospective review of administrative data	Within 28 days of discharge
Suicide Completed	Retrospective review of administrative data	Within 28 days of discharge
Psychological Distress	Kessler Psychological Distress (K10)	One month post-discharge
Quality of Life	ReQoL-10	One month post-discharge

1. Readmission

A minimum recommendation of using retrospective review of administrative data for readmissions within a defined time period, the most agreed was 28 days. Participants indicated that routine data collection might cover slightly different time periods. Twenty-six of the 43 participants recommended a measure of around a month (1 month, 30 days or 28 days) with 28 days being this most popular. However, they also advise that this should be supplemented either by cross-checking with service-users, case managers or carers, where possible to improve quality of data. Those looking for more comprehensive data may also like to record 7 days, 3 months and 6 months as these were also popular recommendations.

2. Quality of Life

The participants recommended that researchers use the Recovering Quality of Life (ReQoL-10) at one month post-discharge [35]. This was the most recommended instrument by the group. However, many participants also voted for ReQoL 20, a large proportion of the group suggested this outcome. As this is a quality of life measure specific to mental health recovery they felt this is most appropriate. The one month time marker is in-keeping with the other COS time frames, making a more comprehensive and accessible core outcome set. Those using within-participant measures of quality of life may like to also measure a pre-discharge baseline. Researchers looking for more thorough assessment of quality of life may like to also measure at 7 days post-discharge and 3 months, as these were also highly recommended time markers or use the ReQoL 20 and report both scores for comparability.

3. Suicide Completed

The participants recommended retrospective review of administrative data, for suicide completed within 28 days of discharge. Retrospective review is in line with other outcomes and was marginally the highest suggestion. We chose within 28 days for consistency with readmission data. Researchers looking for more comprehensive data may want to use 7 days and 3 months as these were highly recommended also. They may also want to cross-check this information against other sources (carers/case managers) to ensure it is correct and

reported, particularly as participants mentioned the impact of incorrect coroner's reports on such data.

4. Psychological Distress

The participants recommended Kessler Psychological Distress (K10) one month post-discharge [36] . For consistency with other outcomes we recommend measure at one month. Seven days and 3 months are also highly recommended, so we would recommend these for research that is more robust. Although there were very few votes for instruments for psychological distress and qualitative comments revealed that participants felt this is not measurable. The same amount of people who voted for K10 also voted for interviews or other measures and a similar amount recommended the development of a new measure, CORE-10 was similarly close [37]. Whilst we make this recommendation, we also suggest that future researchers may look to develop something specific for Psychological Distress in this core outcome set. Interviews would not effectively facilitate the between study comparison, the key purpose of a COS.

Outcome 1: Readmission

Measure	Number of votes	Important comments
Interviews with SUs	12	• In some countries… there is no easily accessible data on readmission rates…in our experience self-reported in the most reliable way
Retrospective review of administrative data	13	• Might not show people who need admission but don't because there's no bed
Extracted from case-managers notes and cross-checked with hospital records	10	• Might be easier to gather administrative data, but worth cross-checking to improve quality of information
Self-reported questionnaire	1	
Other- carer interview	2	
Total	38	

Conclusion: A minimum recommendation of using retrospective review of administrative data. This will allow for various studies with diverse time and financial limits to use the COS. However, we also advise that this should supplemented either by cross-checking with service-users, case managers or carers, where possible to improve quality of data.

Time Markers

Time marker	Number of Votes
Within 2 days	3
Within 3 days	2
Within 7 days	17
Within 28 days	12
Within 30 days	7
Within 1 month	7
Within 6 weeks	4
Within 12 weeks	7
Within 3 months	11
Within 6 months	14
Other (1yr and 3yrs)	1

Around 3 days	5
Around a month	26
Around 3 months	18

Conclusion: The minimum recommendation to record readmission within 28 days. 26 of the 43 participants recommended a measure of around a month (1 month, 30 days or 28 days) with 28 being this most popular. Those looking for higher quality or more comprehensive data may also like to record 7 days, 3 months and 6 months as these were also popular recommendations.

Outcome 2: Quality of Life

Instrument	**Number of votes**	**Comments**
ReQol- 10	9	'I think ReQol would be best for patients in MH services as I understand it was validated for CMHT patients but if someone didn't have contact with MH services before and isn't under a CMHT afterwards another measure might be better.'
ReQol- 20	3	
Quality of Life Brief Version (Lehman)	2	
WHO Quality of Life Scale	5	
Manchester Short Assessment Quality of Life (MANSA)	1	
Satisfaction with Life Scale (SWLS)	1	
SF12	2	
EQ5D	0	
AQoL-8D	0	
Other (ICECAP)	1	
Develop a new tool	2	
No instrument (interviews etc. instead)	7	I think the use of tools should be complemented with interviews with service users and carers.
Total	33	
ReQoL combined (10+20)	12	

Conclusion: We recommend that researchers use ReQoL-10. This was the most voted for instrument. If we also combine the scores with those who voted for ReQoL 20, a large proportion of the group suggested this outcome. As this is a quality of life measure specific to mental health recovery we feel this is most appropriate.

Time markers

**Time Point**	**Number of votes**
Within 2 days	3
Within 3 days	2
Within 7 days	16
Within 28 days	11
Within 30 days	6
Within 1 month	9
Within 6 weeks	3
Within 12 weeks	5
Within 3 months	21
Within 6 months	11
Other (within 9 months, 12 months and 3 years)	2
Pre-discharge	9
Around 3 days	5
Around 1 month	26
Around 3 months	26

Conclusion: We recommend a minimum measure of QoL at one month post-discharge in RCTs. This is in keeping with the readmission time frames, making a more comprehensive and accessible core outcome set. Those using within-participant measures of quality of life may like to

also measure a pre-discharge baseline. Those looking for more thorough assessment of QoL may like to also measure at 7 days post-discharge and 3 months, as these were also highly recommended time markers.

Core Outcome 3: Suicide completed

Measure	Number of votes
Retrospective review of administrative data	18
Extracted from case managers and cross-checked with hospital records	17
Extracted from clinical case notes	14
Other<br>Extracted from serious incident reporting<br>From family and friends	4
Total	53

Conclusion: Retrospective review of administrative data. To keep in line with other outcomes and was marginally highest measure.

Time Marker	Number of votes
Within 2 days	2
Within 3 days	2
Within 7 days	20
Within 28 days	12
Within 30 days	4
Within 1 month	9
Within 6 weeks	0
Within 12 weeks	4
Within 3 months	14
Within 6 months	13
Other	2
Around 3 days	4
Around 1 month	25
Around 3 months	18

Conclusion. Within 28 days for consistency with other outcomes. Other researchers may want to use 7 days and 3 months as these were highly recommended also.

Outcome 4: Psychological Distress

Instrument	Number of votes
Kessler Psychological Distress (K10)	6
CORE-10	5
Brief Psychiatric Rating Scale (BPRS)	3
Brief Symptom Inventory (BSI)	1
The positive and negative symptom scale	1
The Discharge List (DL) â€“ 20 item scale	2
Clinical Global Impression to Assess illness severity	0
ASI (Psychiatric problem subscale)	0
PSYRAT (Psychological Distress subscale)	1
Develop a new self-assessment tool for psychological distress	5
Other	1
No instrument (interview or other method instead)	6
Total	31

Conclusion: We recommend Kessler Psychological Distress (K10). Although there were very few votes for measures. The same amount of people who voted for K10 also voted for interviews or other measures and a similar amount recommended the development of a new measure,

CORE-10 was similarly close. Whilst we make this recommendation, we also suggest that future researchers may look to develop something specific for this core outcome set. Interviews would not allow for easy comparison of scores so would not be relevant for a core outcome set.

Time Markers

Time Marker	Number of votes
Within 2 days	2
Within 3 days	4
Within 7 days	22
Within 28 days	8
Within 30 days	4
Within 1 month	14
Within 6 weeks	3
Within 12 weeks	4
Within 3 months	15
Within 6 months	14
Other	3
Pre-discharge baseline measure at ...	5
Around 3 days	6
Around 1 month	26
Around 3 months	19

Conclusion: The minimum recommendation is one month post-discharge. For consistency with other outcomes we recommend measure at one month. 7 days and 3 months are also highly recommended, so we would recommend these for more thorough research.
