# Supplementary material for: Developing a core outcome set for interventions to improve discharge from mental health inpatient services: a survey, Delphi and consensus meeting with key stakeholder groups
Source: BMJ Open. 2020 May 12;10(5):e034215. doi: 10.1136/bmjopen-2019-034215 (PMC7228512; doi:10.1136/bmjopen-2019-034215)
Supplement: Author's manuscript [file bmjopen-2019-034215.draft_revisions.pdf]

BMJ Open

BMJ Open is committed to open peer review. As part of this commitment we make the peer review history of every article we publish publicly available.

When an article is published we post the peer reviewers' comments and the authors' responses online. We also post the versions of the paper that were used during peer review. These are the versions that the peer review comments apply to.

The versions of the paper that follow are the versions that were submitted during the peer review process. They are not the versions of record or the final published versions. They should not be cited or distributed as the published version of this manuscript.

BMJ Open is an open access journal and the full, final, typeset and author-corrected version of record of the manuscript is available on our site with no access controls, subscription charges or pay-per-view fees (http://bmjopen.bmj.com).

If you have any questions on BMJ Open's open peer review process please email info.bmjopen@bmj.com

BMJ Open

Interventions to Improve Discharge from Acute Adult Mental Health Inpatient Care to the Community: Development of a Core Outcome Set.

Journal:	*BMJ Open*
Manuscript ID	bmjopen-2019-034215
Article Type:	Original research
Date Submitted by the Author:	15-Sep-2019
Complete List of Authors:	Tyler, Natasha; The University of Manchester, NIHR Greater Manchester Patient Safety Translational Research Centre<br>Wright, Nicola; University of Nottingham<br>Waring, Justin ; University of Birmingham<br>Grundy, Andrew; University of Nottingham
Keywords:	MENTAL HEALTH, Core Outcome Set, Discharge, Care Transitions, Acute Adult Mental Health Services

SCHOLARONE™
Manuscripts

I, the Submitting Author has the right to grant and does grant on behalf of all authors of the Work (as defined in the below author licence), an exclusive licence and/or a non-exclusive licence for contributions from authors who are: i) UK Crown employees; ii) where BMJ has agreed a CC-BY licence shall apply, and/or iii) in accordance with the terms applicable for US Federal Government officers or employees acting as part of their official duties; on a worldwide, perpetual, irrevocable, royalty-free basis to BMJ Publishing Group Ltd ("BMJ") its licensees and where the relevant Journal is co-owned by BMJ to the co-owners of the Journal, to publish the Work in this journal and any other BMJ products and to exploit all rights, as set out in our licence.

The Submitting Author accepts and understands that any supply made under these terms is made by BMJ to the Submitting Author unless you are acting as an employee on behalf of your employer or a postgraduate student of an affiliated institution which is paying any applicable article publishing charge ("APC") for Open Access articles. Where the Submitting Author wishes to make the Work available on an Open Access basis (and intends to pay the relevant APC), the terms of reuse of such Open Access shall be governed by a Creative Commons licence – details of these licences and which Creative Commons licence will apply to this Work are set out in our licence referred to above.

Other than as permitted in any relevant BMJ Author's Self Archiving Policies, I confirm this Work has not been accepted for publication elsewhere, is not being considered for publication elsewhere and does not duplicate material already published. I confirm all authors consent to publication of this Work and authorise the granting of this licence.

For peer review only

Title: Interventions to Improve Discharge from Acute Adult Mental Health Inpatient Care to the Community: Development of a Core Outcome Set.

Authors:

Corresponding Author: Natasha Tyler (Greater Manchester Patient Safety Translational Research Centre, University of Manchester, Manchester, UK, Natasha.tyler@manchester.ac.uk, 0161 275 7620)

Nicola Wright (University of Nottingham, Nottingham, UK)

Justin Waring (Health Services Management Centre, University of Birmingham, Birmingham, UK)

Andrew Grundy (University of Nottingham, Nottingham, UK)

For peer review only

Abstract

Objective

To develop a core set of outcomes to be used in all future studies into discharge from acute mental health services to increase homogeneity of outcome reporting.

Design

We used a cross-sectional online survey with qualitative responses to derive a comprehensive list of outcomes, followed by two online Delphi rounds and a face-to-face consensus meeting.

Setting

The setting the core outcome set applies to is acute adult mental health.

Participants

Ninety-three participants in total completed the questionnaire, 69 in Delphi round one, 68 in round two, with relatively even representation of groups. Eleven participants attended the consensus meeting. Participants were from five stakeholder groups: service-users, families and carers, researchers, healthcare professionals and policy makers.

Interventions

The core outcome set is intended for all interventions that aim to improve discharge from acute mental health services to the community.

Results

Service-users, healthcare professionals, researchers, carers/families and end-users of research agreed on a four-item core outcome set: Readmission, Suicide completed, Service-user reported psychological distress and Quality of life.

Conclusion

Implementation of the core outcome set in future trials research will provide a framework to achieve standardisation, facilitate selection of outcome measures, allow between-study comparisons, and ultimately enhance the relevance of trial or research findings to healthcare professionals, researchers, policy makers and service users.

For peer review only

Article Summary

Strengths and Limitations

- This the first initiative to reduce heterogeneity in outcome reporting for interventions that focus on discharge from acute mental health services.
- We achieved a high level of consensus amongst 69 service users, families/carers, healthcare professionals, researchers and policy makers.
- Although the stakeholder group included international researchers, service users and healthcare professionals were from the UK

Funding

This work was funded by the National Institute for Health Research (NIHR) Greater Manchester Patient Safety Translational Research Centre (NIHR Greater Manchester PSTRC). The views expressed are those of the author(s) and not necessarily those of the NHS, the NIHR or the Department of Health and Social Care.

Competing Interests

The authors report no competing interests.

Key Words

- Acute Adult Mental Health Services
- Core Outcome Set
- Mental Health
- Discharge
- Care Transitions

Background

Care transitions (when patient care is transferred from one team, department or organisation to another) are widely recognised as a vulnerable and high-risk stage in the care pathway [1–3]. Safety issues may be intensified in acute mental health services, where care transitions are described as chaotic [3]. For example, suicide risk increases post-discharge from acute mental health services [4,5]. A growing body of research describes these risks either directly in terms of identified 'safety' events or indirectly in terms of broader 'problems', including for example treatment non-adherence, inappropriate readmissions, increased risk of self-injury or suicide attempts [3,6–8].

Internationally, researchers have attempted to find solutions to the problems or threats to safety associated with discharge from acute mental health services by developing interventions that aim to improve different aspects of discharge planning, transitions, continuity of care, and follow-up care. There are various types of discharge intervention presented in the literature [9]. Some interventions aim to improve discharge by introducing new roles, for example a discharge co-ordinator, that co-ordinates care and provides and single point of contact to help navigate the complex system [10]. Others focus on increasing contact between clinical staff and service users, for example using letters, videoconferencing or telephone follow-up [11–13]. Many 'successful' interventions in reducing readmission, bridged the boundaries between ward and community by providing types of ward-based care in the community [14,15] or where community teams lead discharge planning on the wards [16], with a focus on the early development of therapeutic relationships. Other successful interventions aim to solve a particular problem for a smaller group of acute service users, for example those at risk of homelessness, by providing financial and social support [17,18].

There has been little attempt to compare these diverse interventions. Existing reviews have included either a narrow range of studies addressing a single outcome or focus on a specific

For peer review only

time frame in an attempt to synthesise results [8,19]. Comparison and meta-synthesis of effectiveness of interventions has reported limited success. Across the papers included in our systematic review and those by other researchers [1,20], variation in the outcomes reported is substantial. This limits between study comparability and delays advancement in evidence collection. Furthermore, outcomes in these trials were not necessarily representative of the measures that service users would consider important at discharge. Both matters can potentially be addressed with the development of a 'core outcome set', defined as "an agreed, standardised collection of outcomes which should be measured and reported, as a minimum, in all trials for a specific clinical area" [21].

The development and use of 'core outcome sets' has been endorsed as a means to reduce outcome heterogeneity in research, and to increase the relevance of research through the involvement of key stakeholders in its development [22]. There is an emerging body of literature highlighting the difficulties of defining and assessing outcomes in a mental health population [23]. There is also evidence of a lack of agreement amongst key groups about what should be measured and in what capacity and an evident tension between the population health perspective and provision of individualised care [19,23]. One aforementioned previous review identified the need for consensus on outcome definitions in discharge planning interventions [19], similarly a recent Kings Fund report suggested broader consensus upon the outcomes that matter is imperative for advancement [23]. Therefore, generating agreement amongst healthcare professionals, service users, policy makers and researchers is a difficult but imperative task, to enable the useful direction of healthcare services [23]. The difficulties are further exemplified when applied to care transitions, a multi-agency, multi-stage, complex period of the care pathway [3,24]. This paper outlines the development of a core outcome set for research of interventions to improve discharge from acute mental health wards to the community.

Methods

Study overview

The scope of the core outcome set was defined according to the criteria recommended by Core Outcome Measures in Effectiveness Trials (COMET) [25]. The health condition was functional conditions (mental disorders other than dementia, and includes severe mental illness such as schizophrenia). The population was adults aged 18-65, the intervention was any interventions that aimed to improve discharge from an acute mental health setting to the community. The core outcome set was developed using four stages, including service users and healthcare professionals at each stage: (1) a long list of outcomes was generated through systematic review [1] and qualitative survey; (2) the resulting long outcome list was used to populate an online Delphi process; and (3) the results of the Delphi survey were appraised at a consensus meeting and the final core outcome set was established. After the development of the core outcome set a final stage (4), engaged stakeholders to make recommendations for the measurement of the core outcomes. The process included a series of core research team meetings at every stage, the team comprised of a researcher and core outcome set developer, an associate professor in mental health and mental health nurse, a researcher and expert by lived experience of acute services and an expert in patient safety.

Participants

For peer review only - http://bmjopen.bmj.com/site/about/guidelines.xhtml

For the online questionnaires, groups of participants were recruited in various ways in December 2018 to January 2019. Academic researchers were recruited if their research had been included in our systematic review or if they were known researchers in the field identified by the team. End-users of research (policy makers, NGOs, NHS management, commissioners, advocates etc.) were recruited via searching for publicly available contact details or using our team's professional networks or social media. Service users and healthcare professionals were recruited through social media. Twitter was nominated as the primary platform for distribution due to its ability to reach into the specific communities of interest we required: mental health professionals, service users and families/carers. Using social media has been reported as a cost-effective and efficient way to recruit those from potentially stigmatised groups [26]. Further, the peer network structures of social media platforms enable users to recruit other users through sharing links within their networks. In order to reduce attrition in round 3 those who dropped out after the first questionnaire were invited to re-join the panel.

Participants did not fit into distinct homogeneous groups, for example mental health professionals were sometimes also past service users or family members of service users. Similarly researchers had personal experience of inpatient mental health services. Therefore, wherever possible we considered the group as whole and tried not to compare categories.

For the consensus meeting, UK participants were asked in the final round to indicate whether they would be interested in a face-to-face meeting. We invited a random sample of interested participants to attend, that ensured representative of the stakeholder groups. If a participant declined the invite a similarly matched participant was invited.

Stage 1: Gathering information

In addition to the outcomes extracted from the systematic review [1], outcomes of importance to service users, health care professionals, families, researchers and end-users of research were identified through qualitative surveys. Additional file 1, outlines the questions asked to each group, if an individual was associated with numerous stakeholder groups they would be presented with question sets relevant to each group. Informed consent was obtained before questionnaires were answered. Outcomes were identified both indirectly, by extrapolating from service users' experiences (e.g. What would make discharge from an acute mental health ward safe in your opinion?), and directly, by asking specifically about outcomes (e.g. Can you think of any important outcomes to measure in research assessing discharge interventions?). . We used open questions that were developed to elicit potential additional outcomes. The questions were loosely modelled on questions developed for a large scale outcome generation study for a depression core outcome set which were developed with service users and healthcare professionals [27]. The question format was mirrored for a mental health discharge theme and the views of a PPI (patient and public involvement, n=5) group sought to confirm appropriateness of questions and instructions.

Qualitative data was coded to identify outcomes and thematically synthesised [28]. This involved line-by-coding of text and development of descriptive themes, the final stage involved generating analytical themes, which were converted into potential outcomes where applicable. Outcomes from the systematic review [1] and qualitative surveys were combined to generate a long list of outcomes. This list, along with relevant quotes from the qualitative data, was discussed by the core research team in a structured meeting. For each outcome, the group decided whether it should be a stand-alone outcome, combined with other codes of a

For peer review only - http://bmjopen.bmj.com/site/about/guidelines.xhtml

similar thematic nature or removed from the process due to being of limited importance for a core outcome set. For example, we agreed to merge closely related items (e.g. *family relations* and *quality of interpersonal relationships*) and to exclude outcomes considered to be of limited importance (e.g., too specific to a particular group: *Autistic life*; or intervention *Antipsychotic Politherapy*). Unless there was a unanimous decision to merge or remove an outcome, it remained as a stand-alone outcome. The group decisions about each outcome are documented in additional file 2. The final outcome list was used to populate the Delphi questionnaire. The outcomes list and instructions for the questionnaires were reviewed for face validity, understanding, and acceptability by a PPI group and modified according to feedback. The round was open for 6 weeks beginning December 10th 2018.

Stage 2: Delphi survey

We ran the Delphi survey manually using Qualtrics: a secure online hosting platform [29]. Only participants that responded to the questionnaire in stage 1, were invited to take part in first round of the Delphi (stage 2). The Delphi process was conducted across two rounds. In each round, participants were asked whether the items should become part of a core outcome set on a 1–7 scale described as: Strongly Agree (1), Agree (2), Slightly Agree (3), Neither Agree nor disagree (4), Slightly Disagree (5), Disagree (6), Strongly Disagree (7). There was a free-text comments box and participants were encouraged to provide comments that would be fed-back anonymously to the group. Participants could suggest additional outcomes at the end of round one, which were reviewed by the core research team. Any outcome not already represented was added to round two. A link to the survey was sent via email. Each round remained open for 14 days and participants received two follow-up reminder emails. Round one was open from late February 2019 to early March 2019, round two was late March to early April.

In round two median group scores for each outcome and anonymous comments for and against from the previous round were presented and participants were asked to reflect on the information presented and score each outcome again. The percentage of participant agreement with each outcome on a scale of 1–7 was calculated from the scores obtained during round one and again in round two.

Literature suggests that consensus levels should be set a priori at a minimum of 70 percent [25,30]. We unanimously chose a 75% consensus level, slightly higher than the minimum to increase sensitivity, but to still allow for a varied pool of applicable outcomes given the tension in the literature around disagreement between service-user, health professional and policy-makers opinions of mental health outcomes [23]. Consensus criteria were defined a priori: outcomes scored as Agree or Strongly Agree (1-2) by 75% or more of the group reached consensus for inclusion and were included in the provisional core outcome set. Outcomes scored as Disagree or Strongly Disagree (6–7) by 75% or more were defined as having reached consensus for exclusion and were excluded. Outcomes not fulfilling criteria for consensus inclusion or exclusion were defined as not having reached consensus and were re-presented in round two.

As no outcomes met the original criteria for having reached consensus for exclusion after round one, it was agreed by the research team to redefine the criteria for having reached consensus for exclusion if 50% or less of participants scored the item as Strongly Agree or Agree (6-7). Reducing exclusion criteria after round one has been used effectively in past core outcome set research [30].

For peer review only

Stage 3: Consensus meeting

The results of the Delphi survey were presented at a consensus meeting, the main goal of the consensus meeting was to decide which items will be included in the final core outcome set. The format of the consensus meeting comprised of a short overview of the study, a summary of the results of how each stakeholder group had scored each outcome, beginning with the outcomes that met consensus [31]. This was chaired by an independent researcher, with expertise in consensus methodology, and who was not a member of the core research team. Participants were sampled to achieve a balanced representation of service users, health-care professionals, researchers and end-users of research. We aimed to have a small representative group of between 9 and 12 to enable meaningful small group discussions, similar to consensus meetings chaired by the facilitator in other fields [32,33] . International participation was restricted because of budgetary constraints. Outcomes identified in round one and two of the Delphi as having reached consensus for inclusion were presented and participants were asked if there were any fundamental reasons why these should not be included in the core outcome set. Divergent views were actively sought and the chair ensured everyone had opportunity to participate in discussions before voting commenced. Outcomes from the preliminary core outcome set were discussed in terms of feasibility and voted upon. Voting was conducted anonymously using cards in an envelope with bivariate response options (include/exclude). Voting and consensus criteria followed the same format as in the Delphi (75% for inclusion). Results were presented after the voting of all outcomes had finished. Outcomes deemed to be having reached consensus for exclusion or with no consensus in the Delphi were reviewed and participants were asked if there were any fundamental reasons why these should be included in the core outcome set, with individual outcomes being discussed and voted on, only if proposed as being important by a meeting participant. Outcomes meeting criteria for consensus were included in the core outcome set; all other items excluded. The meeting finished with the presentation and ratification (a final review and discussion) of the four-item core outcome set.

Stage 4: Preliminary Measurement Recommendations

After the core outcome set was agreed in the consensus meeting, we invited all participants from the three rounds to recommend measures and time markers in a final online questionnaire. Participants were invited to participate if they had been involved in any of the previous online rounds. The invitation made it clear that the questionnaire is most relevant to researchers, but that other groups with an opinion or interest are welcome to contribute. This was due to the specific knowledge of instruments required to complete this round.

In this questionnaire participants were presented with the four core outcomes. For each core outcome they were presented with any measures used to assess that outcome in our systematic review studies [1] and any additional measures that had been recommended to the team during the process. Participants were asked to choose the one most appropriate, (don't know, other, new instrument, no instrument were also options). A second question also asked which time markers would be recommended, with options to select all applicable. These options were also developed based on time markers used in the systematic review [1].

Ethics and registration

For peer review only

Our findings are reported in line with the Core Outcome Set-Standards for Reporting (COS-STAR) guidance [31]. The study was prospectively registered with the COMET initiative. The study was approved by the University of Nottingham Business School ethics committee.

Role of the funding source

The funding source was not involved in study design; in the collection, analysis, and interpretation of data; in the writing of the report; and in the decision to submit the paper for publication

Results

Stage 1: Information gathering

Our systematic review has been described in detail elsewhere [1]. In summary 69 outcome categories were identified from 45 studies. Ninety-three participants from 12 countries completed the information gathering questionnaire, including 27 identified as service users, 17 family/carers, 39 health-care professionals, 15 end-user of research and 37 researchers. As noted above, many chose multiple categories. Additional file 3 presents participants demographics. Qualitative questionnaires revealed an additional 45 outcomes that were not identified in the literature (for example, outcomes concerning involvement in discharge planning, see additional file 2). After discussion within the research team, 82 standardised outcome terms were taken forward into the Delphi process; 19 outcomes were combined/collapsed and 13 were removed, see additional file 2.

Stage 2: Delphi process

Sixty-nine participants completed round one of the Delphi (22 service users, families and carers, 26 researchers and 21 healthcare professionals and decision makers) and 68 participants completed round two (30 researchers, 18 service users and families and 20 healthcare professionals and decision makers). Whilst 5 participants dropped out after round one, 4 new participants joined the panel in round two. Attrition rate from first questionnaire to round one of the Delphi was 25.8%, there was 1.4% attrition between round one and two of the Delphi. Seven additional outcomes were proposed during round one, of which two were added into round two after a core team discussion. The full list of Delphi items is available in additional file 4.

After round one, 14 outcomes met the criteria for consensus inclusion, these were: Service user involvement in discharge planning; Functioning; Mental health and illness; Personal Recovery; Service user understanding of discharge plan; Quality of life; Suicide Completed; Readmission; Service user involvement in decision making; Service user satisfaction with information provision at discharge; Service user knowledge of how to access community support; Recurrence; Suicide Attempted; Discharge to appropriate accommodation (see table 1). Twenty outcomes met the revised criteria for having reached consensus for exclusion. Forty-eight outcomes did not meet consensus criteria and were re-presented to the group in round two. Therefore, 50 outcomes were presented in round two, only one outcome met the criteria for consensus after this round: meaningful activity. No outcomes met criteria for exclusion and 49 did not meet consensus. Additional file 4 shows consensus levels for each outcome in each round.

For peer review only

Table 1: The Preliminary Core Outcome Set at the end of the online Delphi.

	Percentage agreement	Percentage Disagreement	Median	Researchers	Service users and families	HCPs and DMs
Service user involvement in discharge planning (inc. feeling listened to)	87%	4%	7	65%	100%	95%
Functioning (health, social, etc.)	83%	3%	6	69%	100%	81%
Mental health and illness (symptom/psychological distress)	83%	3%	6	73%	91%	86%
Personal Recovery	82%	1%	6	75%	86%	86%
Service user understanding of discharge plan	81%	3%	6	65%	91%	86%
Quality of life	81%	1%	6.5	65%	90%	86%
Suicide Completed	80%	4%	7	80%	90%	68%
Readmission	80%	6%	6	77%	77%	86%
Service user involvement in decision making (shared decision making)	77%	4%	7	50%	95%	86%
Service user satisfaction with information provision at discharge (e.g. regarding medication, risk, crisis planning)	77%	6%	6	65%	86%	81%
Service user knowledge of how to access community support (i.e. in an emergency)	77%	3%	6	58%	91%	86%
Recurrence (i.e. relapse)	75%	1%	6	58%	91%	76%
Suicide Attempted	75%	4%	6	62%	86%	81%

For peer review only

Discharge to appropriate accommodation	75%	3%	6	69%	91%	67%
Meaningful Activity (included in Round 2)				73%	80%	79%

Stage 3: Consensus Meeting

Eleven participants attended the consensus meeting, as in previous rounds these categories were not exclusive, six participants were researchers, three identified as service users, three as healthcare professionals and three end-users of research, see table 2. Table 3 shows the quantitative results of the meeting.

The preliminary 15-item core outcome set was considered individually and discussions indicated that many of the outcomes were elements of an ideal discharge, and process outcomes/variables, but probably not measurable outcomes that should be included in a core outcome set. After these discussions and independent and anonymous voting, five items no longer met consensus criteria for inclusion. First, 'service user involvement' in discharge planning, and the associated items 'Service user understanding of discharge plan', 'Service user involvement in decision making', 'Service user satisfaction with information provision at discharge'; and 'Service user knowledge of how to access community support'. There was a tremendous agreement amongst the group that these are very important elements of a successful discharge, but not core outcomes due to issues surrounding validity and meaning. In the discussion we present these five outcomes as a potential additional important self-reported measure of service user satisfaction and involvement.

'Mental Health and illness' was initially close to consensus with 73% consensus to include, however those that chose to exclude found it to be too vague, and articulated that they were most interested in measuring acute psychological distress, rather than mental health and illness. The service user representatives in the group interpreted "recovery" to mean a complete amelioration of symptoms and even when in "recovery" individuals described continuing to experience distress and difficulties with their mental health. We chose to therefore separate the broader mental health and illness outcome into self-reported psychological distress and clinician reported mental health. The granular outcome of self-reported psychological distress resulted in 100% consensus to include. On the contrary clinician reported mental health did not meet consensus criteria (45%). Similar discussions happened around the recurrence (relapse) outcome, whereby its inclusion in a core outcome set, would ultimately necessitate buy-in to criteria model, which suggested that mental health problems could and should be completely resolved.

Discussions around the 'suicide attempted' outcome indicated that participants felt that suicide attempts or self-harm had diverse motivations and definitions and they discussed the issues of delineating the boundaries of self-harm and suicide attempts and how this is documented. After the consensus meeting this outcome no-longer meet consensus criteria to include. Discussions surrounding personal recovery, functioning and meaningful activity indicated that participants considered these outcomes too vague and subjective to be a component of a core outcome set. There was consensus to exclude meaningful activity and recovery, and no consensus to include personal recovery. There was consensus to exclude

For peer review only

discharge to appropriate accommodation, discussion indicated this was primarily because this spanned the health and social care boundaries and may not be applicable to every intervention.

On completion of the meeting, only four outcomes met consensus criteria for inclusion, see figure 1. A core outcome set of four was ratified and participants agreed that the following should be included: readmission, quality of life, suicide completed and service user reported psychological distress. Readmission was the most frequently used outcome in past research, and despite limitations, participants felt it was one of the only proxy measures of appropriate discharge. Quality of life and psychological distress were considered important ways of quantitatively assessing the psycho-social elements of discharge; which are of primary importance. Suicide completed was considered rare but imperative data to capture given the research highlighting the relationship between acute mental health discharge and suicide highlighted by a growing body of literature [5,34]. Figure 2 shows the process undertaken to reach the core outcome set.

<Insert Figure 1>

Table 2: Participants that attended consensus meeting

PP Number	Researcher	Service User	Healthcare Professional	End-user of Research
1	X			
2	X			
3	X			
4	X		x	
5	x	X		
6	x			X
7			x	
8			x	x
9		X		
10		X		
11				x
Total	6	3	3	3

Table 3: Outcomes of consensus meeting, levels of consensus in anonymous voting

	Include	Exclude	Percentage
Readmission	10	1	91%
Service user reported psychological distress	11	0	100%
Suicide completed	9	2	82%
QoL	9	2	82%
Reoccurrence	4	7	36%

For peer review only

Mental Health and Illness	8	3	73%
Service user involvement in decision making	7	4	64%
Personal Recovery	6	5	55%
Meaningful Activity	1	10	9%
Functioning	1	10	9%
Clinician Reported Mental Health	5	6	45%
Service User Satisfaction with information provision at discharge	3	8	27%
Service user understanding of the discharge plan	3	8	27%
Suicide Attempted	3	8	27%
Service User Involvement in Discharge Planning	6	5	55%
Knowledge of how to access support in a crisis	5	6	45%
Discharge to appropriate accommodation	0	11	0%

<insert figure 2>

Stage 4: Preliminary Measurement Recommendations

Forty-three of the 93 invited participants responded in the final round (15 service users, 8 family members/carers, 23 researchers, 10 healthcare professionals, 3 end users of research), although as in previous rounds these were not distinct categories. Fifty-three percent of the respondents were researchers, this was expected as in the email we suggested that this stage may be more meaningful or of interest to this group, but as a team we chose not to exclude other groups with opinions on measurement instruments. Twenty-three percent of participants were international researchers (from USA, Switzerland, Canada, and Australia). Table 4 shows the preliminary minimum measure recommendations and time markers, additional file 5 shows the results upon which the recommendations were based.

Table 4: Summary of Measurement Recommendations

Core Outcome	Instrument/Measure	Time Marker
Readmission	Retrospective review of administrative data	Within 28 days of discharge
Suicide Completed	Retrospective review of administrative data	Within 28 days of discharge

For peer review only

Psychological Distress	Kessler Psychological Distress (K10)	One month post-discharge
Quality of Life	ReQoL-10	One month post-discharge

1. Readmission

A minimum recommendation of using retrospective review of administrative data for readmissions within a defined time period, the most agreed was 28 days. Participants indicated that routine data collection might cover slightly different time periods. Twenty-six of the 43 participants recommended a measure of around a month (1 month, 30 days or 28 days) with 28 days being this most popular. However, they also advise that this should be supplemented either by cross-checking with service-users, case managers or carers, where possible to improve quality of data. Those looking for more comprehensive data may also like to record 7 days, 3 months and 6 months as these were also popular recommendations.

2. Quality of Life

The participants recommended that researchers use the Recovering Quality of Life (ReQoL-10) at one month post-discharge [35]. This was the most recommended instrument by the group. However, many participants also voted for ReQoL 20, a large proportion of the group suggested this outcome. As this is a quality of life measure specific to mental health recovery they felt this is most appropriate. The one month time marker is in-keeping with the other COS time frames, making a more comprehensive and accessible core outcome set. Those using within-participant measures of quality of life may like to also measure a pre-discharge baseline. Researchers looking for more thorough assessment of quality of life may like to also measure at 7 days post-discharge and 3 months, as these were also highly recommended time markers or use the ReQoL 20 and report both scores for comparability.

3. Suicide Completed

The participants recommended retrospective review of administrative data, for suicide completed within 28 days of discharge. Retrospective review is in line with other outcomes and was marginally the highest suggestion. We chose within 28 days for consistency with readmission data. Researchers looking for more comprehensive data may want to use 7 days and 3 months as these were highly recommended also. They may also want to cross-check this information against other sources (carers/case managers) to ensure it is correct and reported, particularly as participants mentioned the impact of incorrect coroner's reports on such data.

4. Psychological Distress

The participants recommended Kessler Psychological Distress (K10) one month post-discharge [36] . For consistency with other outcomes we recommend measure at one month. Seven days and 3 months are also highly recommended, so we would recommend these for research that is more robust. Although there were very few votes for instruments for psychological distress and qualitative comments revealed that participants felt this is not measurable. The same amount of people who voted for K10 also voted for interviews or other measures and a similar amount recommended the development of a new measure, CORE-10 was similarly close [37]. Whilst we make this recommendation, we also

For peer review only

suggest that future researchers may look to develop something specific for Psychological Distress in this core outcome set. Interviews would not effectively facilitate the between study comparison, the key purpose of a COS.

Discussion

This study provides the first international consensus on outcomes for intervention studies concerning discharge from an acute adult mental health inpatient setting. We could not identify any other published core outcome sets for interventions concerning discharge from acute mental health services. Moreover, there are very few core outcome sets for mental health, despite recommendations for consensus in the literature [20,23]. All the included outcomes were identified as critically important by more than 75% of a group of relatively equally-represented service-users and family/carers, health-care professionals, researchers and end-user of research using consensus methods. We recommend that all future research studies evaluating interventions for discharge from acute adult mental health settings use this core outcome set as a framework for outcome selection, to compliment, rather than replace any other outcomes that are relevant to their research question. We suggest that researchers can and should chose other outcomes related to their own research question in addition to these four items. As discharge from acute services is a particularly challenging period for those experiencing mental health problems [3,34], it's important to understand what interventions work and more specifically which elements of an intervention improve which particular outcomes. This core outcome set provides a framework for between-study comparison, ultimately enabling researchers to articulate the theory of change that underpins interventions.

In our systematic review [1], we identified 22 studies that reported readmission rates as an outcome, yet almost all of them captured this in different ways: some used self-report data, some clinical case notes or some retrospective administrative data, others used case manager's reports. In addition the time markers were variable, some used country specific time markers in line with policy such as 28 days in the UK [38], whilst others chose a series of time markers such as within 1 month, 3 month and 6 months, but the time markers were rarely directly comparable. Similarly, six studies measured quality of life but, only two used the same measurement instrument (Lehman's Quality of Life) [16,39]. In the current study, we have developed consensus that Quality of Life and Readmission are important and feasible to measure, but we also make recommendations about how to measure them to improve heterogeneity of outcome reporting.

There were some unexpected exclusions in the core outcome set, for example mental health symptoms and treatment adherence were quite frequently used in past research [1], but not included in the core outcome set. At the beginning of the paper we described the recent Kings Fund report that suggested generating agreement amongst healthcare professionals, service users, policy makers and researchers is a difficult but imperative task [23]. We found this reflected here and feel that the small four-item core outcome set represents the only outcomes that are unanimously agreed upon as essential, despite so many outcomes being of upmost importance to service-users and families. This research has further highlighted the importance of shared decision making and service-user and family involvement to all stakeholder groups [40].This consensus study indicates a desire from all groups to monitor levels of service-users satisfaction and involvement in the process. Whilst such outcomes, were excluded in later stages, primarily on the basis of being process variables/outcomes, it does not reduce their

For peer review only

prospective importance in discharge interventions or provision of care at discharge. The five most agreed upon elements of service-user involvement and satisfaction in discharge were: Service user involvement in discharge planning; Service user understanding of discharge plan; Service user involvement in decision making; Service user satisfaction with information provision at discharge; Service user knowledge of how to access community support. We recommend that future policy makers and healthcare management consider incorporating all of these elements into local level initiatives as overriding principles of care rather than interventions/measures, if they are ever missing from current provision. Furthermore, work highlighting the importance of involving service users in mental health care planning is beginning to emerge, along with measures of such activity. Therefore, we suggest that future research wherever possible should include a service user reported outcome measure of involvement alongside the 4-item core outcome set and any other chosen measures. This could be measured in an existing instrument of service-user involvement care planning in mental health, such as the EQUIP PROM (Patient Reported Outcome Measure) [40], or use the 6 outcomes presented above to develop a self-reported Likert measure of service user involvement specifically in discharge planning, as these 6 items are developed from synthesis of academic literature, qualitative questionnaires and met criteria for consensus amongst experts, so from a psychometric perspective would arguably meet initial face and content validity criteria [41], see additional file 6.

The difficulties of developing a mental health core outcome set was further epitomised when applied to care transitions: a service-level (rather than specific clinical population) multi-agency, multi-stage, complex period of the care pathway [3,24]. Generating a set of meaningful applicable outcomes that span primary and secondary care, across multiple physical locations, that are relevant for every service user was imperative. For example, a great deal of past literature focuses on housing interventions [42–44], and whilst housing is a significant safety issue at discharge, it's not necessarily relevant to all service users. This multi-agency, multi-morbidity complexity was arguably one factor that resulted in the small set of generic outcomes, that arguably differs from narrowly defined clinical core outcome set reported in past literature of many more outcomes [45,46].

This study had several strengths. Our method is based on recommendations from an international panel of experts [25]. Inclusion of service-users and health-care professionals at every stage ensured that outcomes in the final core set embody shared priorities. The comprehensive and laborious long-list process ensured all potential outcomes were considered in the course of the consensus process. However, there were some limitations to our study. The research was only conducted in English, due to budgetary constraints, although our online rounds included participants from 12 countries. The attrition rate between the first questionnaire and the first Delphi round could be considered relatively high, however there was little attrition between consensus rounds. The group with the highest level of attrition after round one were service users and families and their replacements in round two were primarily researchers; which could have impacted on the results in round two. Furthermore, in many consensus meetings additional outcomes are often added, the method infrequently serves as means of reducing the number of outcomes included in the preliminary core outcome set from the Delphi [30]. However, in our case we found that the group did not agree with many of the outcomes and it was reduced to a very small COS of 4 items. This is beneficial in some ways, as we hope it is easier for researchers to operationalise a four-item core outcome set. However, to ensure that the spectrum of perceptions of safety by each group is addressed we recommend using the COS alongside an additional service user

For peer review only

reported outcome measure (PROM) that encompasses the other outcomes that had high consensus in the Delphi.

We have made preliminary recommendations of measures and time markers for the four core outcomes, however we recommend that future research tests the feasibility and effectiveness of these measures, with an expectation that they may be revised. The recommendations may be UK-centric, given that only 23% of the panel were international researchers and 28 day readmission rate is in line with UK policy [38]. We therefore invite international researchers to test or comment on the feasibility of this core outcome set outside of the UK. There was also no concrete consensus on any measure or time-marker, therefore we have made recommendations informed by the predominant opinions of experts to ensure that the core outcome set can be operationalised immediately. We acknowledge that it will often be necessary for outcomes and measures to be adapted or augmented in future research to include additional or complementary items, but feel the necessity for homogeneity in outcome reporting is imminent.

The use of outcomes in mental health research and service, is becoming more contested in terms of what is meaningful and effective, it could be argued that core outcomes sets are less applicable to mental health populations than general health populations, given the complexity of mental health problems and the subjectivity of measuring it. However, as core outcomes sets are relatively uncommon in mental health, we believe (similar to other clinical populations) a small, agreed, feasible set of core outcomes will facilitate between study comparability and advancement in evidence collection [21,25].

Future Directions

Development of this core outcome set involved participation of stakeholders from 12 different countries; (primarily researchers) however, we recommend that further work should be undertaken to validate this core outcome set more widely, particularly in non-English speaking populations. The two of the final four outcomes and many of the preliminary 15 outcomes to emerge from the Delphi, are not necessarily specific to mental health care transitions. Some outcomes are comparable to a similar core outcome set for care transitions of adolescents and young adults with special healthcare needs [47]. Future research may consider a 'transitions of care' core outcome set, to reduce the number of similar core outcome sets.

Conclusion

The four outcomes included in our outcome set represent the consensus opinion of a group of service-users, health-care professionals, and international researchers and addresses an unmet necessity: assisting researchers in the design, implementation and reporting of interventions that aim to improve discharge from acute mental health settings. Ultimately, application of this core outcome set will enhance the relevance of future interventions to health-care professionals, the research community and service-users. If used, the core outcome set could provide more evidenced-based interventions, underpinned by theory of change outlining the relationships between the component of the intervention and the outcome it should improve[1,48]; which should increase service-user safety at this distressing time period.

For peer review only

Patients' consent and permission to publish

All participants gave informed consent to be involved in the study ahead of data collection.

Author Contributions

NT conceived the design of the study; conducted the literature search, meta-synthesis and Delphi; analysed the data; and drafted the majority of the manuscript. JW and NW contributed equally and provided oversight of the study design, analysed and synthesised the data and contributed significantly to the drafting of the manuscript. AG analysed and synthesised data, provided an expert by lived experience opinion on decisions made in regards to wording, study deisgn, PPI involvement, also an contributed adaptations to the manuscript.

Conflicts of Interest

The authors report no conflicts of interest

Ethics Committee Approval

This research received a favourable ethics opinion from Nottingham University Business School Ethics Committee.

Role of Funding Source

This research is funded by the NIHR Greater Manchester Patient Safety Translational Research Centre, they had no role in the writing of manuscript or decision to submit.

Data Statement

Data is available upon reasonable request

Word Count: 5858

References

[1] Tyler N, Wright N, Waring J. Interventions to Improve Discharge from Acute Adult Mental Health Inpatient Care to the Community: Systematic Review and Narrative Synthesis. PRE-PRINT (Version 1) n.d.
[2] Waring J, McDonald R, Harrison S. Safety and complexity: Inter-departmental relationships as a threat to patient safety in the operating department. J Heal Organ Manag 2006. doi:10.1108/14777260610662753.
[3] Wright N, Rowley E, Chopra A, Gregoriou K, Waring J. From admission to discharge in mental health services: a qualitative analysis of service user involvement. Heal Expect 2016;19:367–76. doi:10.1111/hex.12361.
[4] Walter F, Carr MJ, Mok PLH, Astrup A, Antonsen S, Pedersen CB, et al. Premature Mortality Among Patients Recently Discharged From Their First Inpatient Psychiatric

For peer review only

Treatment. JAMA PSYCHIATRY 2017;74:485–92. doi:10.1001/jamapsychiatry.2017.0071.

[5] Burns J, Flynn S, Lowe R, Turnbull P, Baird A, Stones P, et al. The National Confidential Inquiry into Suicide and Homicide by People with Mental Illness. 2017.

[6] O. A, M.N. M, A.L. B, J. M, B.A. N. Assessing need for pharmacist involvement to improve care coordination for patients on LAI antipsychotics transitioning from hospital to home: A work system approach. Res Social Adm Pharm 2017;13:1004–13. doi:http://dx.doi.org/10.1016/j.sapharm.2017.02.006.

[7] Exbrayat S, Coudrot C, Gourdon X, Gay A, Sevos J, Pellet J, et al. Effect of telephone follow-up on repeated suicide attempt in patients discharged from an emergency psychiatry department: a controlled study. BMC Psychiatry 2017;17. doi:10.1186/s12888-017-1258-6.

[8] Vigod SN, Kurdyak PA, Dennis C-L, Leszcz T, Taylor VH, Blumberger DM, et al. Transitional interventions to reduce early psychiatric readmissions in adults: systematic review. Br J Psychiatry 2013;202:187–94. doi:10.1192/bjp.bp.112.115030.

[9] Waring J, Bishop S, Marshall F, Tyler N, Vickers R. An ethnographic study comparing approaches to inter-professional knowledge sharing and learning in discharge planning and care transitions. J Heal Organ Manag n.d.

[10] Walker SA, Naji SA, Howie FL, Andrew J, Cameron IM, Eagles JM. The effectiveness of psychiatric discharge coordinators. Prim Care Psychiatry 2000. doi:10.1185/135525700543370.

[11] Bennewith O, Evans J, Donovan J, Paramasivan S, Owen-Smith A, Hollingworth W, et al. A Contact-Based Intervention for People Recently Discharged from Inpatient Psychiatric Care: A Pilot Study. Arch Suicide Res 2014. doi:10.1080/13811118.2013.838196.

[12] D'souza R. Improving Treatment Adherence and Longitudinal Outcomes in Patients with a Serious Mental Illness by Using Telemedicine. J Telemed Telecare 2002. doi:10.1177/1357633X020080S251.

[13] Exbrayat S, Coudrot C, Gourdon X, Gay A, Sevos J, Pellet J, et al. Effect of telephone follow-up on repeated suicide attempt in patients discharged from an emergency psychiatry department: A controlled study. BMC Psychiatry 2017. doi:10.1186/s12888-017-1258-6.

[14] Forchuk C, Martin ML, Chan YL, Jensen E. Therapeutic relationships: From psychiatric hospital to community. J Psychiatr Ment Health Nurs 2005. doi:10.1111/j.1365-2850.2005.00873.x.

[15] Juven-Wetzler A, Bar-Ziv D, Cwikel-Hamzany S, Abudy A, Peri N, Zohar J. A pilot study of the " Continuation of Care" model in " revolving-door" patients. Eur Psychiatry 2012. doi:10.1016/j.eurpsy.2011.05.003.

[16] Jensen E, Chapman P, Davis A, Forchuk C, Seymour B, Witcher P, et al. An Evaluation of Community-Based Discharge Planning in Acute Mental Health Care. Can J Community Ment Heal 2010;29:111–24. doi:10.7870/cjcmh-2010-0038.

[17] Shaffer SL, Hutchison SL, Ayers AM, Goldberg RW, Herman D, Duch DA, et al. Brief critical time intervention to reduce psychiatric rehospitalization. Psychiatr Serv 2015;66:1155–61. doi:http://dx.doi.org/10.1176/appi.ps.201400362.

[18] Tomita A, Herman DB. The Impact of Critical Time Intervention in Reducing Psychiatric Rehospitalization After Hospital Discharge. Psychiatr Serv 2012. doi:10.1176/appi.ps.201100468.

[19] Steffen S, Kösters M, Becker T, Puschner B. Discharge planning in mental health care: A systematic review of the recent literature. Acta Psychiatr Scand 2009;120:1–9. doi:10.1111/j.1600-0447.2009.01373.x.

For peer review only

[20] S. S, M. K, T. B. Discharge planning in mental health care: A systematic review of the recent literature. Acta Psychiatr Scand 2009;120:1–9. doi:http://dx.doi.org/10.1111/j.1600-0447.2009.01373.x.
[21] Williamson PR, Altman DG, Bagley H, Barnes KL, Blazeby JM, Brookes ST, et al. The COMET Handbook: Version 1.0. Trials 2017. doi:10.1186/s13063-017-1978-4.
[22] Williamson PR, Altman DG, Blazeby JM, Clarke M, Devane D, Gargon E, et al. Developing core outcome sets for clinical trials: Issues to consider. Trials 2012. doi:10.1186/1745-6215-13-132.
[23] Lang K. Outcomes for mental health services: what really matters? | HSR UK. 2019.
[24] Waring J, Marshall F, Bishop S, Sahota O, Walker M, Currie G, et al. An ethnographic study of knowledge sharing across the boundaries between care processes, services and organisations: the contributions to 'safe' hospital discharge. Heal Serv Deliv Res 2014. doi:10.3310/hsdr02290.
[25] Kirkham JJ, Davis K, Altman DG, Blazeby JM, Clarke M, Tunis S, et al. Core Outcome Set-STAndards for Development: The COS-STAD recommendations. PLoS Med 2017. doi:10.1371/journal.pmed.1002447.
[26] Pedersen ER, Helmuth ED, Marshall GN, Schell TL, PunKay M, Kurz J. Using Facebook to Recruit Young Adult Veterans: Online Mental Health Research. JMIR Res Protoc 2015. doi:10.2196/resprot.3996.
[27] CONTRIBUTED TALKS. J Evid Based Med 2019;12:24–7. doi:10.1111/jebm.12336.
[28] Thomas J, Harden A. Methods for the thematic synthesis of qualitative research in systematic reviews. BMC Med Res Methodol 2008;8:45. doi:10.1186/1471-2288-8-45.
[29] Qualtrics. Qualtrics: The World's Leading Research & Insights Platform. Qualtrics 2016. doi:10.3109/03639045.2013.790903.
[30] Hsu C, Sandford B. The delphi technique: making sense of consensus. Pract Assessment, Res Eval 2007;12:1–8. doi:10.1016/S0169-2070(99)00018-7.
[31] Kirkham JJ, Gorst S, Altman DG, Blazeby J, Clarke M, Devane D, et al. COS-STAR: A reporting guideline for studies developing core outcome sets (protocol). Trials 2015. doi:10.1186/s13063-015-0913-9.
[32] Bell BG, Spencer R, Avery AJ, Campbell SM. Tools for measuring patient safety in primary care settings using the RAND/UCLA appropriateness method. BMC Fam Pract 2014;15:110. doi:10.1186/1471-2296-15-110.
[33] Avery AJ, Dex GM, Mulvaney C, Serumaga B, Spencer R, Lester HE, et al. Development of prescribing-safety indicators for GPs using the RAND Appropriateness Method. Br J Gen Pract 2011;61:e526–36. doi:10.3399/bjgp11X588501.
[34] Walter F, Carr MJ, Mok PLH, Antonsen S, Pedersen CB, Shaw J, et al. Suicide Methods and Specific Types of Accidental Death and Fatal Poisoning Among Discharged Psychiatric Patients: A National Cohort Study. J Clin Psychiatry 2018;79. doi:10.4088/JCP.17m11809.
[35] Keetharuth AD, Brazier J, Connell J, Bjorner JB, Carlton J, Buck ET, et al. Recovering Quality of Life (ReQoL): A new generic self-reported outcome measure for use with people experiencing mental health difficulties. Br J Psychiatry 2018. doi:10.1192/bjp.2017.10.
[36] Andrews G, Slade T. Interpreting scores on the Kessler Psychological Distress Scale (K10). Aust N Z J Public Health 2001. doi:10.1111/j.1467-842X.2001.tb00310.x.
[37] Barkham M, Bewick B, Mullin T, Gilbody S, Connell J, Cahill J, et al. The CORE-10: A short measure of psychological distress for routine use in the psychological therapies. Couns Psychother Res 2013. doi:10.1080/14733145.2012.729069.
[38] Ali AM, Bottle A. How do we interpret readmission rates? BMJ 2018.

For peer review only

doi:10.1136/bmj.k3104.
[39] Tomita A, Lukens EP, Herman DB. Mediation analysis of critical time intervention for persons living with serious mental illnesses: assessing the role of family relations in reducing psychiatric rehospitalization. Psychiatr Rehabil J 2014;37:4–10. doi:10.1037/prj0000015.
[40] Lovell K, Bee P, Brooks H, Cahoon P, Callaghan P, Carter L-A, et al. Embedding shared decision-making in the care of patients with severe and enduring mental health problems: The EQUIP pragmatic cluster randomised trial. PLoS One 2018;13:e0201533. doi:10.1371/journal.pone.0201533.
[41] Falzarano M, health GZ-J of allied, 2013 undefined. Seeking consensus through the use of the Delphi technique in health sciences research. IngentaconnectCom n.d.
[42] C. F, G. R, S. K-M, K. T. From psychiatric ward to the streets and shelters. J Psychiatr Ment Health Nurs 2006;13:301–8. doi:http://dx.doi.org/10.1111/j.1365-2850.2006.00954.x.
[43] Forchuk C, Godin M, Hoch JS, Kingston-MacClure S, Jeng M (Mo) S, Puddy L, et al. Preventing Homelessness After Discharge from Psychiatric Wards: Perspectives of Consumers and Staff. J Psychosoc Nurs Ment Health Serv 2013;51:24–31. doi:10.3928/02793695-20130130-02.
[44] Tomita A, Herman DB. The Role of a Critical Time Intervention on the Experience of Continuity of Care Among Persons With Severe Mental Illness After Hospital Discharge. J Nerv Ment Dis 2015;203:65–70. doi:10.1097/NMD.0000000000000224.
[45] Fish R, Sanders C, Adams R, Brewer J, Brookes ST, DeNardo J, et al. A core outcome set for clinical trials of chemoradiotherapy interventions for anal cancer (CORMAC): a patient and health-care professional consensus. Lancet Gastroenterol Hepatol 2018. doi:10.1016/S2468-1253(18)30264-4.
[46] Boers M, Brooks P, Simon LS, Strand V, Tugwell P. OMERACT: An international initiative to improve outcome measurement in rheumatology. Clin Exp Rheumatol 2005;23. doi:10.1186/1745-6215-8-38.
[47] Fair C, Cuttance J, Sharma N, Maslow G, Wiener L, Betz C, et al. International and Interdisciplinary Identification of Health Care Transition Outcomes. JAMA Pediatr 2016;170:205. doi:10.1001/jamapediatrics.2015.3168.
[48] Davidoff F, Dixon-Woods M, Leviton L, Michie S. Demystifying theory and its use in improvement. BMJ Qual Saf 2015;24:228–38. doi:10.1136/bmjqs-2014-003627.

For peer review only

Formatted: Font: (Default) Times New Roman

Core Outcome Set

1. Readmission
2. Quality of Life
3. Suicide Completed
4. Service User Reported Psychological Distress

For peer review only

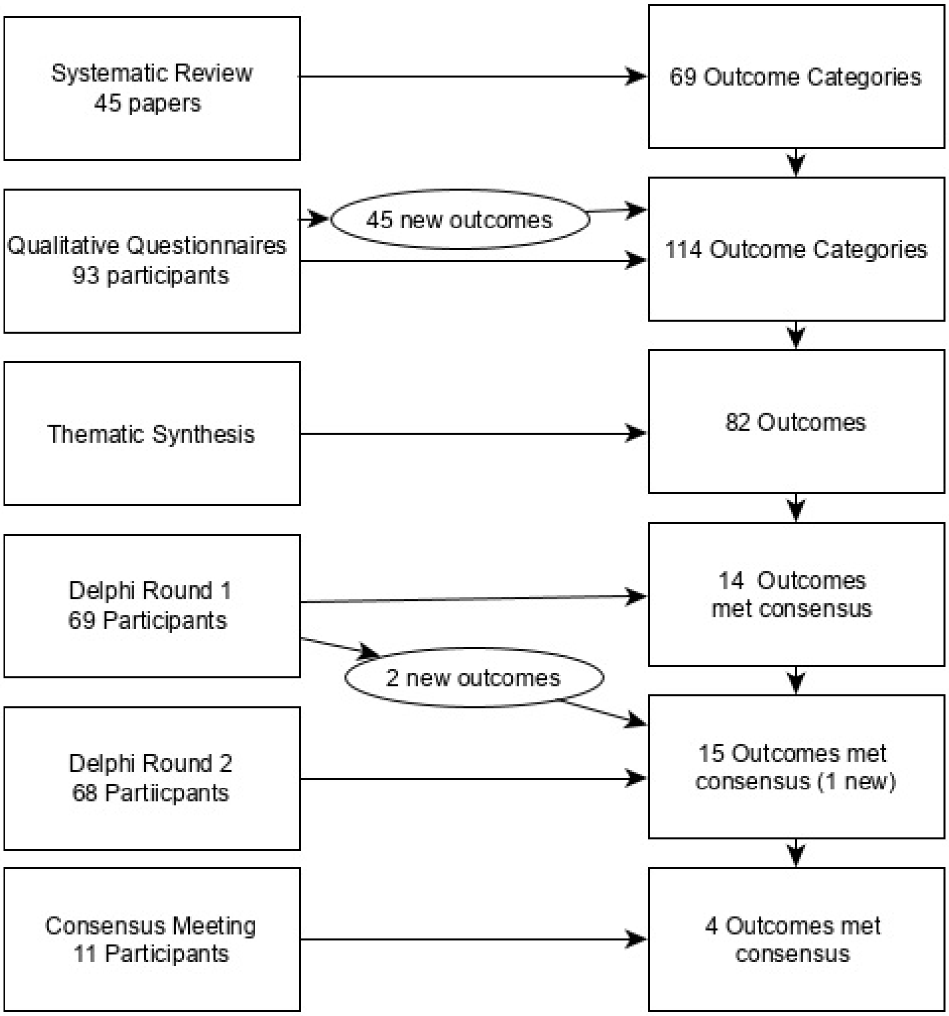

Process of reducing the outcomes to a set of four core outcomes

133x142mm (96 x 96 DPI)

1

Supplementary Material

File 1: Table of questions asked in qualitative questionnaire

Group	Question
All Participants	What would make discharge from an acute mental health ward **safe** in your opinion?
All Participants	What would make discharge from an acute mental health ward **effective** in your opinion?
All Participants	What would you like to see prioritised for research into discharge from an acute ward?
All Participants	Can you think of any important outcomes to measure in research assessing discharge interventions?
Service User	What do you think is the most difficult aspect of discharge from a mental health acute ward?
Service User	What might improve discharge from a mental health ward?
Service User	Was there any one person/group/intervention which really made a difference for you? If so what was it? What made it so powerful?
Service User	Do you have any other feelings or concerns not addressed in previous questions?
Family member/carer	What do you consider to be the most difficult components of discharge for your family member/friend from an acute mental health ward?
Family member/carer	What types of improvement would you expect to see in your family member/friend following a successful discharge from an acute mental health ward?
Mental health professional	What do you consider to be the most difficult aspects of discharge from an acute unit for your service-users?
Mental health professional	Are there any changes/behaviours you would expect to see in a service-user during or following a safe and/or effective discharge?
Researchers	If applicable, what outcomes did you measure in past research (of discharge interventions)?
Researchers	Are there any concepts that you think are important to measure, but chose not to, due to not having a suitable measurement instrument?
End users of research	If you were looking to use research to inform changes to the discharge procedure within your professional role, what outcomes would you like to see reported?
End users of research	In your opinion, what measurements would persuade you that a discharge intervention is effective?

For peer review only

File 2: Results of Meta-synthesis of Outcomes and data from questionnaire

Clinical

From Review	Additional from Meta-synthesis
Reoccurrence (i.e. Relapse)	Stability
Clinical Recovery	Physical Health and Wellbeing
Personal Recovery	Iatrogenic harm (patient safety incidents- harm caused by the care system)
Substance Misuse (inc. alcohol)	Serious accidental injury
Self-injury (i.e. harm to self)	Mortality
Suicide Risk	Mental health and illness (symptoms/psychological distress)
Suicidal Ideation	
Suicide Attempted	
Suicide Completed	
Medication Knowledge	
Medication Adherence	
Medication Side Effects	
Medication Management	
Global Functioning	

Service

From Review	Additional from Meta-synthesis
Emergency department visits post discharge	Police intervention post discharge
Readmission	Engagement with psychological intervention
Length of stay pre-discharge	Engagement with community services
Length of stay post-discharge (i.e. subsequent readmissions)	Availability of appropriate community support (i.e. allocated key worker)
Outpatient appointment/visit adherence	Length of time before follow up/aftercare
Service use	Leaving the hospital against medical advice
	Service user involvement in decision making (shared decision making)
	Serious incidents (i.e. serious incidents reported formally)

Satisfaction

From Review	Additional from Meta-synthesis
Service user expectations of care	Professionals/care teams satisfaction with information provision at discharge
Service user satisfaction with discharge	Service user satisfaction with information provision at discharge (e.g. regarding medication, risk, crisis planning)
Service user experience of discharge	
Service user satisfaction with treatment	
Carer/family/other satisfaction with discharge	
Staff satisfaction with discharge	

Personal

From Review	Additional from Meta-synthesis
Hopelessness	Feelings of safety
Boredom	Feelings of support (from various groups- i.e. family, professionals and friends)
Loneliness	Experience of stigma
Isolation	Quality of interpersonal relationships (friends and family)
Coping skills	Self-management
Concern/anxiety about discharge	
Autonomy (e.g. independence, autonomous decision making)	

For peer review only - http://bmjopen.bmj.com/site/about/guidelines.xhtml

Housing Stability	
Discharge to appropriate accommodation	
Service user knowledge of own condition	
Quality of life	
Community participation	
Violence	
Aggression	
Victim of crime	
Stable relationships with health professionals	
Employment	

Discharge Planning

From Review	Additional from Meta-synthesis
	Service user involvement in discharge planning
	Family/carer involvement in discharge planning
	Advocate involvement in discharge planning
	Completion of planned care
	Continuity of contact
	Service user readiness for discharge (incl. preparation)
	Clinical readiness for discharge
	Experience of coercion at discharge (e.g. feeling forced to leave)
	Provision of financial support (e.g. benefits advice, debt advice)
	Delays in expected discharge
	Service availability (e.g. availability of community services, CPN, follow-up, social worker, bed in residential service)
	Family/carer/other readiness for discharge
	Staff understanding of safe and effective discharge
	Plan for community engagement
	Is there a plan for care post discharge created
	Has the plan for care post-discharge been communicated with patient
	Has information about the discharge been reported to other relevant services

Changes/Combinations

Outcomes from Review	Changes/Combinations
Social Recovery	Combined into quality of interpersonal relationships
Relapse	PPI suggestion change to reoccurrence
Self-harm	PPI suggestion change to self-injury
Depression	Combined into symptoms
Anxiety	Combined into symptoms
Illegal drug use	Combined into substance misuse
Alochol use	Combined into substance misuse
Addiction severity	Combined into substance misuse
Crisis planning	Combined into information provision
Risk communication	Combined into information provision
Allocated worker	Combined into availability of appropriate community support
Better knowledge transfer	Combined into information provision (professional)
Therapeutic alliance	Combined into stable relationships with HPs
Contact with ambulatory care	Combined into emergency visits
Treatment adherence	Combined into medication adherence and engagement with psychological services
7 day follow up	Changed to length of time before follow up for global audience
Psychological distress	Combined into symptoms
Family relations	Combined into quality of interpersonal relationships
Community integration and functioning	Combined into Community participation

Outcomes Removed from Process

Outcomes from Review	Reason
Antipsychotic Politherapy	Too specific
Autistic life	Too specific

For peer review only

Medication Problems	Too vague
Functional Recovery	Meaning not explicit
Satisfaction with service in community	Not relevant for this COS
Global satisfaction with discharge	Meaning not explicit
Quality of care	Too vague
Treatment decision making	Not relevant for this COS
Financial cost to provider	Impossible to measure
Number of beds	Too vague
Staff workload	Too vague
Freedom	Too vague
Self-activity	Too vague

Carer outcomes

**Outcomes from Review**	**Decision**
Caregiver Burden	Yes
Caregiver Health Status	No (too specific)
Caregiver Knowledge about illness	Yes
**From Synthesis**	
Carer support	No
Relationship between family and SU	No (probably a variable)

For peer review only

File 3: Participant Demographics

**Categories**											
**Group**	**Service Users**	**Families and Carers**	**Healthcare Professionals**	**Researchers**	**End-users of Research**						
**n**	27	17	39	37	15						
**Gender**	**Male**	**Female**	**Other**								
**n**	28	63	0								
**Age**	**18-24**	**25-34**	**35-44**	**45-54**	**55-64**	**65-74**	**75-84**				
**n**	4	20	30	24	10	4	1				
**Location**	**International**	**East of England**	**East Midlands**	**London**	**North West**	**North East**	**South East**	**South West**	**Wales**	**West Midlands**	**Yorkshire and the Humber**
**n**	24	2	11	7	16	1	6	5	0	13	7

Locations for International participants

**Country**	**N**
Australia	4
Canada	2
China	1
France	1
Germany	1
Iran	1
Italy	1
Northern Ireland	1
South Africa	1
Switzerland	4
USA	7

For peer review only

File 4: Full list of Delphi outcomes, consensus levels, and round of inclusion/exclusion

	R1 Percentage agreement	R1 Percentage Disagreement	Median	Researchers	Sus and carers	HCPs and DMs	Round 2 Status	Round 2	Researcher	Sus and carers	HCP and DMs	Median
Service user involvement in discharge planning (inc. feeling listened to)	87%	4%	7	65%	100%	95%	Include					
Functioning (health, social, etc.)	83%	3%	6	69%	100%	81%	Include					
Mental health and illness (symptom/psychological distress)	83%	3%	6	73%	91%	86%	Include					
Personal Recovery	82%	1%	6	75%	86%	86%	Include					
service user understanding of discharge plan	81%	3%	6	65%	91%	86%	Include					
Quality of life	81%	1%	6.5	65%	90%	86%	Include					
suicide Completed	80%	4%	7	80%	90%	68%	Include					
Readmission	80%	6%	6	77%	77%	86%	Include					
service user involvement in decision making (shared decision making)	77%	4%	7	50%	95%	86%	Include					
service user satisfaction with information provision at discharge (e.g. regarding medication, risk, crisis planning)	77%	6%	6	65%	86%	81%	Include					
service user knowledge of how to access community support (i.e. in an emergency)	77%	3%	6	58%	91%	86%	Include					
Recurrence (i.e. relapse)	75%	1%	6	58%	91%	76%	Include					
Suicide Attempted	75%	4%	6	62%	86%	81%	Include					
discharge to appropriate accommodation	75%	3%	6	69%	91%	67%	Include					
service user satisfaction with treatment	74%	7%	6	58%	91%	71%	Re-present	59%	43%	67%	70%	6
service Availability (e.g. availability of community service, CPN, follow-up, social worker,	74%	7%	6	46%	86%	95%	Re-present	45%	27%	67%	50%	5

bed in residential service)												
Existence of a plan for community engagement/ care post discharge	74%	4%	6	58%	77%	90%	Re-present	72%	57%	83%	90%	6
Feeling of support (from various group i.e. family, professional, friends)	72%	4%	6	65%	77%	71%	Re-present	43%	33%	50%	50%	5
Suicide Risk	71%	3%	6	58%	77%	81%	Re-present	42%	43%	56%	25%	5
Family/carer/other involvement in discharge planning (inc. feeling listened too)	71%	6%	6	65%	77%	75%	Re-present	58%	50%	67%	65%	6
service user readiness for discharge (incl. preparation)	71%	6%	6	58%	77%	81%	Re-present	35%	27%	50%	30%	5
Feeling of safety	70%	7%	6	65%	73%	71%	Re-present	36%	37%	56%	25%	5
service user satisfaction with discharge	69%	4%	6	50%	76%	86%	Re-present	45%	40%	50%	50%	5
Physical Health and Wellbeing	68%	3%	6	50%	77%	81%	Re-present	49%	43%	61%	45%	5
service user experience of discharge	68%	10%	6	46%	86%	76%	Re-present	29%	23%	44%	30%	5
Housing stability	68%	3%	6	62%	77%	67%	Re-present	41%	37%	39%	40%	5
Coping skills	68%	6%	6	50%	77%	80%	Re-present	39%	40%	50%	35%	5
Continuity of contact	68%	6%	6	64%	77%	62%	Re-present	65%	63%	72%	60%	6
Mortality	67%	7%	6	68%	76%	57%	Re-present	42%	43%	50%	30%	5
self management	67%	4%	6	46%	86%	71%	Re-present	29%	23%	39%	20%	5
Length of time before follow up/aftercare	65%	6%	6	46%	77%	76%	Re-present	58%	43%	83%	65%	6
Isolation	65%	9%	6	46%	73%	81%	Re-present	55%	50%	72%	45%	6
Availability of appropriate community support (i.e. allocated key worker)	64%	9%	6	38%	82%	76%	Re-present	52%	27%	72%	70%	6
service user knowledge of own condition	64%	6%	6	42%	91%	62%	Re-present	36%	30%	50%	35%	4
service user expectation of care	63%	3%	6	35%	76%	81%	Re-present	28%	20%	28%	40%	4
Hopelessness	63%	9%	6	54%	67%	71%	Re-present	46%	47%	44%	45%	5
suicidal Ideation	62%	7%	6	46%	77%	67%	Re-present	51%	50%	61%	45%	6

For peer review only

Medication Adherence	62%	4%	6	50%	82%	52%	Re-present	45%	47%	44%	40%	5
Loneliness	62%	10%	6	42%	68%	81%	Re-present	41%	37%	56%	30%	5
Information about the discharge reported to other relevant service	62%	9%	6	50%	73%	71%	Re-present	48%	37%	78%	40%	5
service user experience of coercion at discharge	61%	6%	6	46%	77%	63%	Re-present	43%	40%	67%	35%	5
Clinical readiness for discharge	60%	6%	6	48%	67%	67%	Re-present	36%	30%	39%	40%	5
Medication side Effects	59%	7%	6	38%	77%	67%	Re-present	41%	30%	61%	35%	5
Emergency department visit post discharge	59%	6%	6	58%	68%	52%	Re-present	62%	63%	67%	60%	6
Quality of interpersonal relationship (friend and family)	59%	7%	6	50%	77%	52%	Re-present	46%	40%	72%	35%	5
Iatrogenic harm (patient safety incident or harm caused by the care system)	59%	4%	6	40%	64%	71%	Re-present	51%	37%	72%	50%	6
Completion of planned care	59%	7%	6	50%	76%	52%	Re-present	39%	40%	56%	30%	5
Concern/anxiety about discharge	58%	9%	6	31%	73%	76%	Re-present	26%	17%	33%	35%	4
Autonomy (e.g. independence, autonomous decision making)	57%	3%	6	35%	73%	70%	Re-present	36%	27%	50%	30%	5
staff understanding of safe and effective discharge	57%	10%	6	40%	68%	67%	Re-present	41%	30%	67%	35%	4
self injury (i.e. harm to self)	57%	6%	6	50%	64%	52%	Re-present	49%	53%	61%	35%	5
Financial support (e.g. benefit advice, debt advice)	57%	9%	6	38%	82%	57%	Re-present	46%	40%	72%	30%	5
stable relationship with health professional	55%	4%	6	38%	71%	58%	Re-present	33%	37%	50%	20%	5
Medication Management	54%	3%	6	42%	71%	52%	Re-present	42%	37%	61%	40%	5
Carer/family/other satisfaction with discharge	54%	9%	6	35%	68%	57%	Re-present	36%	27%	61%	35%	4
Clinical Recovery	53%	7%	6	60%	55%	43%	Re-present	36%	33%	50%	30%	5
service use	52%	4%	6	46%	50%	63%	Re-present	30%	33%	39%	15%	4
Violence	52%	9%	6	46%	60%	48%	Re-present	29%	33%	33%	15%	4
Victim of crime	52%	12%	6	38%	68%	48%	Re-present	28%	30%	44%	10%	4
Experience of stigma	52%	7%	6	42%	59%	57%	Re-present	30%	20%	50%	25%	4

For peer review only

Engagement with psychological intervention	51%	4%	6	35%	45%	76%	Re-present	33%	30%	67%	5%	4
Cancellation or change to follow up meeting	51%	12%	6	19%	82%	57%	Re-present	42%	23%	67%	40%	5
serious incident (i.e. serious incident reported formally)	50%	6%	5.5	44%	45%	62%	Exclude					
Employment	50%	7%	5.5	58%	48%	38%	Exclude					
Medication Knowledge	49%	9%	5	35%	64%	52%	Exclude					
Engagement with community service	49%	4%	5	35%	50%	71%	Exclude					
Length of stay pre-discharge	46%	10%	5	38%	55%	48%	Exclude					
Family/carer/other readiness for discharge	46%	9%	5	42%	55%	43%	Exclude					
Leaving the hospital against medical advice	45%	14%	5	46%	55%	33%	Exclude					
Outpatient appointment/visit adherence	44%	4%	5	54%	57%	24%	Exclude					
Aggression	44%	10%	5	42%	43%	48%	Exclude					
Delay in expected discharge	43%	13%	5	12%	67%	57%	Exclude					
substance Misuse (incl. alcohol)	41%	9%	5	28%	50%	48%	Exclude					
serious accidental injury	40%	12%	5	25%	36%	62%	Exclude					
Community participation	39%	6%	5	35%	36%	48%	Exclude					
stability	38%	4%	5	13%	59%	47%	Exclude					
Advocate involvement in discharge planning	37%	12%	5	24%	45%	45%	Exclude					
Professional/care team satisfaction with information provision at discharge	36%	13%	5	19%	45%	48%	Exclude					
Primary Care/Community service/Nongovernmental or Charity service satisfaction	35%	16%	5	15%	50%	48%	Exclude					
Police intervention post discharge	34%	16%	5	35%	32%	35%	Exclude					
staff satisfaction with discharge	28%	16%	5	19%	36%	29%	Exclude					
Boredom	26%	25%	4	12%	36%	33%	Exclude					
Meaningful Activity (i.e. employment, studying, volunteering)	New outcome proposed in round 1						Include	77%	73%	83.33%	80%	6
Resilience	New outcome proposed in round 1				Do not present							
Dual harm (i.e. a person harms themselves and others)	New outcome proposed in round 1						Exclude	32%	23%	55.56%	30%	4

For peer review only

Annual admissions	New outcome proposed in round 1	Do not present							
Harm to others (violence, criminal or risk-taking behaviour)	New outcome proposed in round 1	Do not present							
Levels of patient confidentiality	New outcome proposed in round 1	Do not present							
Personal learning	New outcome proposed in round 1	Do not present							

For peer review only

File 5: Outcome Measure Recommendation Results

Outcome 1: Readmission

Measure	Number of votes	Important comments
Interviews with SUs	12	• In some countries… there is no easily accessible data on readmission rates…in our experience self-reported in the most reliable way
Retrospective review of administrative data	13	• Might not show people who need admission but don't because there's no bed
Extracted from case-managers notes and cross-checked with hospital records	10	• Might be easier to gather administrative data, but worth cross-checking to improve quality of information
Self-reported questionnaire	1	
Other- carer interview	2	
Total	38	

Conclusion: A minimum recommendation of using retrospective review of administrative data. This will allow for various studies with diverse time and financial limits to use the COS. However, we also advise that this should supplemented either by cross-checking with service-users, case managers or carers, where possible to improve quality of data.

Time Markers

Time marker	Number of Votes
Within 2 days	3
Within 3 days	2
Within 7 days	17
Within 28 days	12
Within 30 days	7
Within 1 month	7
Within 6 weeks	4
Within 12 weeks	7
Within 3 months	11
Within 6 months	14
Other (1yr and 3yrs)	1
Around 3 days	5
Around a month	26
Around 3 months	18

Conclusion: The minimum recommendation to record readmission within 28 days. 26 of the 43 participants recommended a measure of around a month (1 month, 30 days or 28 days) with 28 being this most popular. Those looking for higher quality or more comprehensive data may also like to record 7 days, 3 months and 6 months as these were also popular recommendations.

Outcome 2: Quality of Life

Instrument	**Number of votes**	**Comments**
ReQol- 10	9	'I think ReQol would be best for patients in MH services as I understand it was validated for CMHT patients but if someone didn't have contact with MH services before and isn't under a CMHT afterwards another measure might be better.'
ReQol- 20	3	
Quality of Life Brief Version (Lehman)	2	
WHO Quality of Life Scale	5	
Manchester Short Assessment Quality of Life (MANSA)	1	

For peer review only

Satisfaction with Life Scale (SWLS)	1	
SF12	2	
EQ5D	0	
AQoL-8D	0	
Other (ICECAP)	1	
Develop a new tool	2	
No instrument (interviews etc. instead)	7	I think the use of tools should be complemented with interviews with service users and carers.
Total	33	
ReQoL combined (10+20)	12	

Conclusion: We recommend that researchers use ReQoL-10. This was the most voted for instrument. If we also combine the scores with those who voted for ReQoL 20, a large proportion of the group suggested this outcome. As this is a quality of life measure specific to mental health recovery we feel this is most appropriate.

Time markers

Time Point	Number of votes
Within 2 days	3
Within 3 days	2
Within 7 days	16
Within 28 days	11
Within 30 days	6
Within 1 month	9
Within 6 weeks	3
Within 12 weeks	5
Within 3 months	21
Within 6 months	11
Other (within 9 months, 12 months and 3 years)	2
Pre-discharge	9
Around 3 days	5
Around 1 month	26
Around 3 months	26

Conclusion: We recommend a minimum measure of QoL at one month post-discharge in RCTs. This is in keeping with the readmission time frames, making a more comprehensive and accessible core outcome set. Those using within-participant measures of quality of life may like to also measure a pre-discharge baseline. Those looking for more thorough assessment of QoL may like to also measure at 7 days post-discharge and 3 months, as these were also highly recommended time markers.

Core Outcome 3: Suicide completed

Measure	Number of votes
Retrospective review of administrative data	18
Extracted from case managers and cross-checked with hospital records	17
Extracted from clinical case notes	14
Other<br>Extracted from serious incident reporting<br>From family and friends	4
Total	53

Conclusion: Retrospective review of administrative data. To keep in line with other outcomes and was marginally highest measure.

Time Marker	Number of votes
Within 2 days	2
Within 3 days	2
Within 7 days	20
Within 28 days	12

For peer review only

Within 30 days	4
Within 1 month	9
Within 6 weeks	0
Within 12 weeks	4
Within 3 months	14
Within 6 months	13
Other	2
Around 3 days	4
Around 1 month	25
Around 3 months	18

Conclusion. Within 28 days for consistency with other outcomes. Other researchers may want to use 7 days and 3 months as these were highly recommended also.

Outcome 4: Psychological Distress

Instrument	Number of votes
Kessler Psychological Distress (K10)	6
CORE-10	5
Brief Psychiatric Rating Scale (BPRS)	3
Brief Symptom Inventory (BSI)	1
The positive and negative symptom scale	1
The Discharge List (DL) â€“ 20 item scale	2
Clinical Global Impression to Assess illness severity	0
ASI (Psychiatric problem subscale)	0
PSYRAT (Psychological Distress subscale)	1
Develop a new self-assessment tool for psychological distress	5
Other	1
No instrument (interview or other method instead)	6
Total	31

Conclusion: We recommend Kessler Psychological Distress (K10). Although there were very few votes for measures. The same amount of people who voted for K10 also voted for interviews or other measures and a similar amount recommended the development of a new measure, CORE-10 was similarly close. Whilst we make this recommendation, we also suggest that future researchers may look to develop something specific for this core outcome set. Interviews would not allow for easy comparison of scores so would not be relevant for a core outcome set.

Time Markers

Time Marker	Number of votes
Within 2 days	2
Within 3 days	4
Within 7 days	22
Within 28 days	8
Within 30 days	4
Within 1 month	14
Within 6 weeks	3
Within 12 weeks	4
Within 3 months	15
Within 6 months	14
Other	3
Pre-discharge baseline measure at ...	5
Around 3 days	6
Around 1 month	26
Around 3 months	19

For peer review only

Conclusion: The minimum recommendation is one month post-discharge. For consistency with other outcomes we recommend measure at one month. 7 days and 3 months are also highly recommended, so we would recommend these for more thorough research.

File 6: Proposed potential service user discharge experience PROM that includes items of most importance to stakeholders in addition to the core outcome set

Item	Strongly Agree	Agree	Neither Agree nor Disagree	Disagree	Strongly Disagree
1. I felt involved in my discharge planning					
2. I understood my discharge plan					
3. I know how to access support in the community					
4. I was frequently involved in decisions about my care at discharge					
5. I was discharged to appropriate accommodation					
6. I was satisfied with the information provided to me at discharge					

For peer review only

For peer review only

For peer review only

BMJ Open

Developing a Core Outcome Set for Interventions to Improve Discharge from Acute Mental Health Inpatient Services: A Survey, Delphi and Consensus Meeting with Key Stakeholder Groups.

Journal:	*BMJ Open*
Manuscript ID	bmjopen-2019-034215.R1
Article Type:	Original research
Date Submitted by the Author:	06-Jan-2020
Complete List of Authors:	Tyler, Natasha; The University of Manchester, NIHR Greater Manchester Patient Safety Translational Research Centre<br>Wright, Nicola; University of Nottingham<br>Grundy, Andrew; University of Nottingham<br>Waring, Justin ; University of Birmingham
**Primary Subject Heading**:	Mental health
Secondary Subject Heading:	Health services research
Keywords:	MENTAL HEALTH, Core Outcome Set, Discharge, Care Transitions, Acute Adult Mental Health Services

SCHOLARONE™
Manuscripts

I, the Submitting Author has the right to grant and does grant on behalf of all authors of the Work (as defined in the below author licence), an exclusive licence and/or a non-exclusive licence for contributions from authors who are: i) UK Crown employees; ii) where BMJ has agreed a CC-BY licence shall apply, and/or iii) in accordance with the terms applicable for US Federal Government officers or employees acting as part of their official duties; on a worldwide, perpetual, irrevocable, royalty-free basis to BMJ Publishing Group Ltd ("BMJ") its licensees and where the relevant Journal is co-owned by BMJ to the co-owners of the Journal, to publish the Work in this journal and any other BMJ products and to exploit all rights, as set out in our licence.

The Submitting Author accepts and understands that any supply made under these terms is made by BMJ to the Submitting Author unless you are acting as an employee on behalf of your employer or a postgraduate student of an affiliated institution which is paying any applicable article publishing charge ("APC") for Open Access articles. Where the Submitting Author wishes to make the Work available on an Open Access basis (and intends to pay the relevant APC), the terms of reuse of such Open Access shall be governed by a Creative Commons licence – details of these licences and which Creative Commons licence will apply to this Work are set out in our licence referred to above.

Other than as permitted in any relevant BMJ Author's Self Archiving Policies, I confirm this Work has not been accepted for publication elsewhere, is not being considered for publication elsewhere and does not duplicate material already published. I confirm all authors consent to publication of this Work and authorise the granting of this licence.

For peer review only

Title: Developing a Core Outcome Set for Interventions to Improve Discharge from Acute Mental Health Inpatient Services: A Survey, Delphi and Consensus Meeting with Key Stakeholder Groups.

Authors:

Corresponding Author: Natasha Tyler (Greater Manchester Patient Safety Translational Research Centre, University of Manchester, Manchester, UK, Natasha.tyler@manchester.ac.uk, 0161 275 7620)

Nicola Wright (University of Nottingham, Nottingham, UK)

Andrew Grundy (University of Nottingham, Nottingham, UK)

Justin Waring (Health Services Management Centre, University of Birmingham, Birmingham, UK)

For peer review only

Abstract

Objective

To develop a core set of outcomes to be used in all future studies into discharge from acute mental health services to increase homogeneity of outcome reporting.

Design

We used a cross-sectional online survey with qualitative responses to derive a comprehensive list of outcomes, followed by two online Delphi rounds and a face-to-face consensus meeting.

Setting

The setting the core outcome set applies to is acute adult mental health.

Participants

Participants were from recruited from five stakeholder groups: service-users, families and carers, researchers, healthcare professionals and policy makers.

Interventions

The core outcome set is intended for all interventions that aim to improve discharge from acute mental health services to the community.

Results

Ninety-three participants in total completed the questionnaire, 69 in Delphi round 1, and 68 in round 2, with relatively even representation of groups. Eleven participants attended the consensus meeting. Service-users, healthcare professionals, researchers, carers/families and end-users of research agreed on a four-item core outcome set: Readmission, Suicide completed, Service-user reported psychological distress and Quality of life.

Conclusion

Implementation of the core outcome set in future trials research will provide a framework to achieve standardisation, facilitate selection of outcome measures, allow between-study comparisons, and ultimately enhance the relevance of trial or research findings to healthcare professionals, researchers, policy makers and service users.

Article Summary

Strengths and Limitations

- This is the first initiative to reduce heterogeneity in outcome reporting for interventions that improve discharge from acute mental health services.
- We achieved a high level of consensus amongst 69 service users, families/carers, healthcare professionals, researchers and policy makers.
- Although the stakeholder group included international researchers, service users and healthcare professionals were from the UK

For peer review only

Funding

This work was funded by the National Institute for Health Research (NIHR) Greater Manchester Patient Safety Translational Research Centre (NIHR Greater Manchester PSTRC). The views expressed are those of the author(s) and not necessarily those of the NIHR or the Department of Health and Social Care.

Competing Interests

The authors report no competing interests.

Key Words

- Acute Adult Mental Health Services
- Core Outcome Set
- Mental Health
- Discharge
- Care Transitions

For peer review only

Background

Care transitions (when patient care is transferred from one team, department or organisation to another) are widely recognised as a vulnerable and high-risk stage in the care pathway [1–3]. Safety issues may be intensified in acute mental health services, where care transitions are described as chaotic [3]. For example, suicide risk increases post-discharge from acute mental health services [4,5]. A growing body of research describes these risks either directly in terms of identified 'safety' events or indirectly in terms of broader 'problems', including for example treatment non-adherence, inappropriate readmissions, increased risk of self-injury or suicide attempts [3,6–8].

Internationally, researchers have attempted to find solutions to the problems or threats to safety associated with discharge from acute mental health services by developing interventions that aim to improve different aspects of discharge planning, transitions, continuity of care, and follow-up care. There are various types of discharge intervention presented in the literature [9]. Some interventions aim to improve discharge by introducing new roles, for example a discharge co-ordinator, that co-ordinates care and provides and single point of contact to help navigate the complex system [10]. Others focus on increasing contact between clinical staff and service users, for example using letters, videoconferencing or telephone follow-up [11–13]. Many 'successful' interventions in reducing readmission, bridged the boundaries between ward and community by providing types of ward-based care in the community [14,15] or where community teams lead discharge planning on the wards [16], with a focus on the early development of therapeutic relationships. Other successful interventions aim to solve a particular problem for a smaller group of acute service users, for example those at risk of homelessness, by providing financial and social support [17,18].

There has been little attempt to compare these diverse interventions. Existing reviews have included either a narrow range of studies addressing a single outcome or focus on a specific time frame in an attempt to synthesise results [8,19]. Comparison and meta-synthesis of effectiveness of interventions has reported limited success. Across the papers included in our systematic review and those by other researchers [1,20], variation in the outcomes reported is substantial. This limits between study comparability and delays advancement in evidence collection. Furthermore, outcomes in these trials were not necessarily representative of the measures that service users would consider important at discharge. Both matters can potentially be addressed with the development of a 'core outcome set', defined as "an agreed, standardised collection of outcomes which should be measured and reported, as a minimum, in all trials for a specific clinical area" [21].

The development and use of 'core outcome sets' has been endorsed as a means to reduce outcome heterogeneity in research, and to increase the relevance of research through the involvement of key stakeholders in its development [22]. There is an emerging body of literature highlighting the difficulties of defining and assessing outcomes in a mental health population [23]. There is also evidence of a lack of agreement amongst key groups about what should be measured and in what capacity and an evident tension between the population health perspective and provision of individualised care [19,23]. One aforementioned previous review identified the need for consensus on outcome definitions in discharge planning interventions [19], similarly a recent Kings Fund report suggested broader consensus upon the outcomes that matter is imperative for advancement [23]. Therefore, generating agreement amongst healthcare professionals, service users, policy makers and researchers is a difficult but imperative task, to enable the useful direction of healthcare services [23]. The difficulties are further exemplified when applied to care transitions, a multi-agency, multi-stage, complex period of the care pathway [3,24]. This paper outlines the development of a core outcome set for research of interventions to improve discharge from acute mental health wards to the community.

Methods

Study overview

The scope of the core outcome set was defined according to the criteria recommended by Core Outcome Measures in Effectiveness Trials (COMET) [25]. The health condition was functional conditions (mental disorders other than dementia, and includes severe mental illness such as schizophrenia). The population was adults aged 18-65, the intervention was any interventions that aimed to improve discharge from an acute mental health setting to the community. The core outcome set was developed using four stages, including service users and healthcare professionals at each stage: (1) a long list of outcomes was generated through a systematic review [1] and qualitative survey; (2) the resulting long outcome list was used to populate an online Delphi process (2 rounds); and (3) the results of the Delphi survey were appraised at a consensus meeting and the final core outcome set was established. After the development of the core outcome set a final stage (4), engaged stakeholders to make recommendations for the measurement of the core outcomes. The process included a series of core research team meetings at every stage, the team comprised of a researcher and core outcome set developer, an associate professor in mental health and mental health nurse, a researcher and expert by lived experience of acute services and an expert in patient safety. Participants did not fit into distinct homogeneous groups, for example mental health professionals were sometimes also past service users or family members of service users.

For peer review only

Similarly researchers had personal experience of inpatient mental health services. Therefore, wherever possible we considered the group as whole and tried not to compare categories.

Participants

Participants were recruited in a number of ways in December 2018 to January 2019. Academic researchers were recruited if their research had been included in our systematic review or if they were known researchers in the field identified by the team. End-users of research (policy makers, NGOs, NHS management, commissioners, advocates etc.) were recruited via searching for publicly available contact details or using our team's professional networks or social media. Service users and healthcare professionals were recruited through social media. Twitter was nominated as the primary platform for recruitment due to its ability to reach into the specific communities of interest we required: mental health professionals, service users and families/carers. Using social media has been reported as a cost-effective and efficient way to recruit those from potentially stigmatised groups [26]. Further, the peer network structures of social media platforms enable users to recruit other users through sharing links within their networks.

The same participant group was used throughout the iterative research process, therefore, in order to reduce attrition, those who dropped out in early rounds were invited to re-join the panel in subsequent rounds. Participants were recruited for the consensus meeting during the Delphi, UK participants were asked to indicate whether they would be interested in a face-to-face meeting. We invited a random sample of interested participants to attend, that ensured representative of the stakeholder groups. If a participant declined the invite a similarly matched participant was invited from the Delphi panel principally, or the teams wider network.

Stage 1: Gathering information

In addition to the outcomes extracted from the systematic review [1], outcomes of importance to each stakeholder group were identified through qualitative surveys. For the main body of the questionnaire, we used open questions that were developed to elicit potential additional outcomes. The questions were loosely modelled on questions developed for a large scale outcome generation study for a depression core outcome set which were developed with service users and healthcare professionals [27]. The question format was mirrored, but adapted for a mental health discharge theme. The views of a PPI (patient and public involvement), group sought to confirm appropriateness of questions and instructions (n=5).

After reading a participant information sheet and giving informed consent (by ticking a box), participants selected their stakeholder group (s) and watched a video that describes core outcome sets to non-experts. All participants were then presented with four open-ended questions relating to safe and effective discharge (see additional file 1). Participants were later presented with 3-5 questions specifically developed for their stakeholder group, additional file 1 outlines all of the questions. If a participant were a member of more than one group, they answered questions relevant to multiple groups. Participants also answered a number of demographic questions: years of experience, country of residence, area of UK (if applicable), gender, age and email address for follow-up. The round was open for 6 weeks beginning December 10th 2018.

Qualitative data was coded to identify outcomes and thematically synthesised [28]. This involved line-by-line coding of text and development of descriptive themes, the final stage involved generating analytical themes, which were converted into potential outcomes where applicable. Outcomes two were identified both indirectly, by extrapolating from service users' experiences (e.g. What would make discharge from an acute mental health ward safe in your opinion?), and directly, by asking specifically about outcomes (e.g. Can you think of any important outcomes to measure in research assessing discharge interventions?).

Outcomes from the systematic review [1] and qualitative surveys were combined to generate a long list of outcomes. This list, along with relevant quotes from the qualitative data, was discussed by the core research team in a structured meeting. Each outcome was considered in turn and each member had the opportunity to present arguments for or against inclusion. For each outcome, the group decided whether it should be a stand-alone outcome, combined with other codes of a similar thematic nature or removed from the process due to being of limited importance for a core outcome set. For example, we agreed to merge closely related items (e.g. *family relations* and *quality of interpersonal relationships*) and to exclude outcomes considered to be of limited importance (e.g., specific to a specialised area of care: *Autistic life*; or intervention *Antipsychotic Politherapy*). Unless there was a unanimous decision to merge or remove an outcome, it remained as a stand-alone outcome. The group decisions about each outcome are documented in additional file 2.

Stage 2: Delphi survey

The Delphi technique is a research method aimed at generating consensus. It solicits opinions from stakeholders groups in an iterative process of answering questions. After each round the responses are summarised and redistributed for discussion in the next round. We chose to have two rounds of Delphi in this study. The final outcome list that was decided upon after the group discussion in stage 1 was used to develop the first Delphi questionnaire. Any outcomes without consensus after the first round, were re-presented in round 2. The outcome list and instructions for the questionnaires were reviewed for face validity, understanding, and acceptability by a PPI group (n=5) and modified according to feedback.

A link to the survey was sent via email. Each round remained open for 14 days and participants received two follow-up reminder emails. Round 1 was open from late February 2019 to early March 2019, round 2 was late March 2019 to early April 2019. We ran the Delphi survey manually using Qualtrics: a secure online hosting platform [29]. In each round, participants were asked whether the items should become part of a core outcome set. A 7-point Likert scale was used, described as: Strongly Agree (1), Agree (2), Slightly Agree (3), Neither Agree nor disagree (4), Slightly Disagree (5), Disagree (6), Strongly Disagree (7). There is no definitive research indicating the optimal number of points to have on a Likert scale but scales between 5 and 9 points have been suggested as having the best reliability, so we chose a 7 point scale [30]. There was a free-text comments box and participants were encouraged to provide comments that would be fed-back anonymously to the group. Participants could suggest additional outcomes at the end of round 1, which were reviewed by the core research team. Any outcome not already represented was added to round 2.

For peer review only

In round 2 median group scores for each outcome and anonymous comments for and against from the previous round were presented and participants were asked to reflect on the information presented and score each outcome again. The percentage of participant agreement with each outcome on a scale of 1–7 was calculated from the scores obtained during round 1 and again in round 2.

Literature suggests that consensus levels should be set a priori at a minimum of 70 percent [25,31]. We unanimously chose a 75% consensus level, slightly higher than the minimum to increase sensitivity, but to still allow for a varied pool of applicable outcomes given the tension in the literature around disagreement between service-user, health professional and policy-makers opinions of mental health outcomes [23]. Consensus criteria were defined a priori: outcomes scored as Agree or Strongly Agree (6-7) by 75% or more of the group reached consensus for inclusion and were included in the provisional core outcome set. Outcomes scored as Disagree or Strongly Disagree (1-2) by 75% or more were defined as having reached consensus for exclusion and were excluded. Outcomes not fulfilling criteria for consensus inclusion or exclusion were defined as not having reached consensus and were re-presented in round 2.

As no outcomes met the original criteria for having reached consensus for exclusion after round 1, it was agreed by the research team to redefine the criteria for having reached consensus for exclusion if 50% or less of participants scored the item as Strongly Agree or Agree (6-7). Reducing exclusion criteria after round 1 has been used effectively in past core outcome set research [30].

Stage 3: Consensus meeting

The results of the Delphi survey were presented at a consensus meeting. The main goal of the consensus meeting was to decide which items will be included in the final core outcome set. This was chaired by an independent researcher with expertise in consensus methodology, and who was not a member of the core research team. Participants were sampled to achieve a balanced representation of service users, health-care professionals, researchers and end-users of research. We aimed to have a small representative group of between 9 and 12 to enable meaningful small group discussions, similar to consensus meetings chaired by the facilitator in other fields [32,33]. International participation was restricted because of budgetary constraints.

The format of the consensus meeting comprised of a) a short overview of the study and b) a summary of the Delphi results sorted by stakeholder group, beginning with the outcomes that met consensus [34]. Outcomes identified in round 1 and 2 of the Delphi as having reached consensus for inclusion were presented first. Participants were asked if there were any fundamental reasons why these should not be included in the core outcome set. Divergent views were actively sought and the chair ensured everyone had opportunity to participate in discussions before voting commenced. Outcomes from the preliminary core outcome set were discussed in terms of feasibility and voted upon. Voting was conducted anonymously using cards in an envelope with bivariate response options (include/exclude). Voting and consensus criteria followed the same format as in the Delphi (75% for inclusion). Results were presented after the voting of all outcomes had finished. Outcomes deemed to be having reached consensus for exclusion or with no consensus in the Delphi were reviewed and participants were asked if there were any fundamental reasons why these should be included

For peer review only - http://bmjopen.bmj.com/site/about/guidelines.xhtml

in the core outcome set. Individual outcomes were discussed only if proposed as being important by a meeting participant. Outcomes meeting criteria for consensus were included in the core outcome set; all other items excluded. The meeting finished with the presentation and a final review and discussion of the core outcome set.

Patient and Public Involvement

Five patient representatives worked with researchers to develop the online questionnaires. Patients were represented alongside professionals and researchers in the consensus panel. One member of the research team (and co-author) is an expert by lived experience and was involved in all design and analysis decisions.

Ethics and registration

Our findings are reported in line with the Core Outcome Set-Standards for Reporting (COS-STAR) guidance [34]. The study was prospectively registered with the COMET initiative. The study was approved by the University of Nottingham Business School ethics committee.

Role of the funding source

The funding source was not involved in study design; in the collection, analysis, and interpretation of data; in the writing of the report; and in the decision to submit the paper for publication

Results

Stage 1: Information gathering

Our systematic review has been described in detail elsewhere [1]. In summary 69 outcome categories were identified from 45 studies. Ninety-three participants in total, from 12 countries completed the information gathering questionnaire. However, as aforementioned, many identified with more than one stakeholder group, therefore we do not have absolute homogenous stakeholder group numbers, 27 identified as service users, 17 family/carers, 39 health-care professionals, 15 end-user of research and 37 researchers. Additional file 3 presents participants demographics. Qualitative questionnaires revealed an additional 45 outcomes that were not identified in the literature (for example, outcomes concerning involvement in discharge planning, see additional file 2). After discussion within the research team, 82 standardised outcome terms were taken forward into the Delphi process; 19 outcomes were combined/collapsed and 13 were removed, see additional file 2.

Stage 2: Delphi process

Sixty-nine participants completed round 1 of the Delphi (22 service users, families and carers, 26 researchers and 21 healthcare professionals and decision makers) and 68 participants completed round 2 (30 researchers, 18 service users and families and 20 healthcare professionals and decision makers). Whilst 5 participants dropped out after round 1, 4 participants joined the panel in round 2 (this individuals participated the qualitative questionnaire but not round 1). There was a 1.4% attrition between round 1 and 2 of the Delphi. Seven additional outcomes were proposed by participants during round 1, of which

For peer review only - http://bmjopen.bmj.com/site/about/guidelines.xhtml

two were added into round 2 after a core team discussion. The full list of Delphi items is available in additional file 4.

After round 1, 14 outcomes met the criteria for consensus inclusion (75% or more agreed/strongly agree with that outcome), these were: Service user involvement in discharge

	Percentage agreement	Percentage Disagreement	Median	Researchers	Service users and familiess	HCPs and DMs
Service user involvement in discharge planning (inc. feeling listened to)	87%	4%	7	65%	100%	95%
Functioning (health, social, etc.)	83%	3%	6	69%	100%	81%
Mental health and illness (symptom/psychological distress)	83%	3%	6	73%	91%	86%
Personal Recovery	82%	1%	6	75%	86%	86%
Service user understanding of discharge plan	81%	3%	6	65%	91%	86%
Quality of life	81%	1%	6.5	65%	90%	86%
Suicide Completed	80%	4%	7	80%	90%	68%
Readmission	80%	6%	6	77%	77%	86%
Service user involvement in decision making (shared decision making)	77%	4%	7	50%	95%	86%

planning; Functioning; Mental health and illness; Personal Recovery; Service user understanding of discharge plan; Quality of life; Suicide Completed; Readmission; Service user involvement in decision making; Service user satisfaction with information provision at discharge; Service user knowledge of how to access community support; Recurrence; Suicide Attempted; Discharge to appropriate accommodation (see table 1). Twenty outcomes met the revised criteria for having reached consensus for exclusion (50% or less of participants agreed/strongly agreed with that outcome). Forty-eight outcomes did not meet consensus criteria for inclusion or exclusion and were re-presented to the group in round 2. Therefore, 50 outcomes were presented in round 2, only one outcome met the criteria for consensus after this round: meaningful activity. No outcomes met criteria for exclusion and 49 did not meet consensus. Additional file 4 shows consensus levels for each outcome in each round.

Table 1: The Preliminary Core Outcome Set at the end of the online Delphi.

For peer review only

Service user satisfaction with information provision at discharge (e.g. regarding medication, risk, crisis planning)	77%	6%	6	65%	86%	81%
Service user knowledge of how to access community support (i.e. in an emergency)	77%	3%	6	58%	91%	86%
Recurrence (i.e. relapse)	75%	1%	6	58%	91%	76%
Suicide Attempted	75%	4%	6	62%	86%	81%
Discharge to appropriate accommodation	75%	3%	6	69%	91%	67%
Meaningful Activity (included in Round 2)				73%	80%	79%

Stage 3: Consensus Meeting

Eleven participants attended the consensus meeting, as in previous rounds these categories were not exclusive, six participants were researchers, three identified as service users, three as healthcare professionals and three end-users of research, see table 3. Table 4 shows the quantitative results of the meeting.

The preliminary 15-item core outcome set was considered individually and discussions indicated that many of the outcomes were elements of an ideal discharge, and process outcomes/variables, but probably not measurable outcomes that should be included in a core outcome set. After these discussions and independent and anonymous voting, five items no longer met consensus criteria for inclusion. First, 'service user involvement' in discharge planning, and the associated items 'Service user understanding of discharge plan', 'Service user involvement in decision making', 'Service user satisfaction with information provision at discharge'; and 'Service user knowledge of how to access community support'. There was a discussion that these are very important elements of a successful discharge, but not core outcomes due to issues surrounding validity and meaning. In the discussion of this paper, we present these five outcomes as a potential self-reported measure of service user satisfaction and involvement, which could be used in addition to the core outcome set.

'Mental health and illness' was initially close to consensus with 73% consensus to include, however those that chose to exclude found it to be too vague, and articulated that they were most interested in measuring acute psychological distress, rather than mental health and illness. The service user representatives in the group interpreted "recovery" to mean a complete amelioration of symptoms and even when in "recovery" individuals described continuing to experience distress and difficulties with their mental health. We chose to therefore separate the broader mental health and illness outcome into self-reported

psychological distress and clinician reported mental health. The granular outcome of self-reported psychological distress resulted in 100% consensus to include. On the contrary clinician reported mental health did not meet consensus criteria (45%). Similar discussions happened around the recurrence (relapse) outcome, whereby its inclusion in a core outcome set, would ultimately necessitate buy-in to criteria model, which suggested that mental health problems could and should be completely resolved.

Discussions around the ‘suicide attempted’ outcome indicated that participants felt that suicide attempts or self-harm had diverse motivations and definitions and they discussed the issues of delineating the boundaries of self-harm and suicide attempts and how this is documented. After the consensus meeting this outcome no-longer meet consensus criteria to include. Discussions surrounding personal recovery, functioning and meaningful activity indicated that participants considered these outcomes too vague and subjective to be a component of a core outcome set. There was consensus to exclude meaningful activity and recovery, and no consensus to include personal recovery. There was consensus to exclude discharge to appropriate accommodation, discussion indicated this was primarily because this spanned the health and social care boundaries and may not be applicable to every intervention.

On completion of the meeting, only four outcomes met consensus criteria for inclusion, see Table 2. A core outcome set of four was agreed, participants agreed that the following should be included: readmission, quality of life, suicide completed and service user reported psychological distress. Readmission was the most frequently used outcome in past research, and despite limitations, participants felt it was one of the only proxy measures of appropriate discharge. Quality of life and psychological distress were considered important ways of quantitatively assessing the psycho-social elements of discharge; which are of primary importance. Suicide completed was considered rare but imperative data to capture given the research highlighting the relationship between acute mental health discharge and suicide highlighted by a growing body of literature [5,35]. Figure 1 shows the process undertaken to reach the core outcome set.

Table 2: The final core outcome set

Final Core Outcome Set	
1	Readmission
2	Quality of Life
3	Suicide Completed
4	Service User Reported Psychological Distress

Table 3: Participants that attended consensus meeting

PP Number	Researcher	Service User	Healthcare Professional	End-user of Research
1	X			
2	X			

For peer review only

3	X			
4	X		X	
5	X	X		
6	X			X
7			X	
8			X	X
9		X		
10		X		
11				X
Total	6	3	3	3

Table 4: Outcomes of consensus meeting, levels of consensus in anonymous voting

	Include	Exclude	Percentage
Readmission	10	1	91%
Service user reported psychological distress	11	0	100%
Suicide completed	9	2	82%
QoL	9	2	82%
Reoccurrence	4	7	36%
Mental Health and Illness	8	3	73%
Service user involvement in decision making	7	4	64%
Personal Recovery	6	5	55%
Meaningful Activity	1	10	9%
Functioning	1	10	9%
Clinician Reported Mental Health	5	6	45%
Service User Satisfaction with information provision at discharge	3	8	27%
Service user understanding of the discharge plan	3	8	27%
Suicide Attempted	3	8	27%
Service User Involvement in Discharge Planning	6	5	55%
Knowledge of how to access support in a crisis	5	6	45%

For peer review only

Discharge to appropriate accommodation	0	11	0%

<insert figure 1>

Discussion

This study provides the first international consensus on outcomes for intervention studies concerning discharge from an acute adult mental health inpatient setting. We could not identify any other published core outcome sets for interventions concerning discharge from acute mental health services. Moreover, there are very few core outcome sets for mental health, despite recommendations for consensus in the literature [20,23]. All the included outcomes were agreed upon by more than 75% of a group of relatively equally-represented service-users and family/carers, health-care professionals, researchers and end-user of research using consensus methods. We recommend that all future research studies evaluating interventions for discharge from acute adult mental health settings use this core outcome set as a framework for outcome selection, to compliment, rather than replace any other outcomes that are relevant to their research question. As discharge from acute services is a particularly challenging period for those experiencing mental health problems [3,35], it's important to understand what interventions work and more specifically which elements of an intervention improve which particular outcomes. This core outcome set provides a framework for between-study comparison, ultimately enabling researchers to articulate the theory of change that underpins interventions.

In our systematic review [1], we identified 22 studies that reported readmission rates as an outcome, yet almost all of them captured this in different ways: some used self-report data, some clinical case notes or some retrospective administrative data, others used case manager's reports. In addition the time markers were variable, some used country specific time markers in line with policy such as 28 days in the UK [36], whilst others chose a series of time markers such as within 1 month, 3 month and 6 months, but the time markers were rarely directly comparable. Similarly, six studies measured quality of life but, only two used the same measurement instrument (Lehman's Quality of Life) [16,37]. In the current study, we have developed consensus that Quality of Life and Readmission are important and feasible to measure, but we also make recommendations about how to measure them to improve heterogeneity of outcome reporting.

There were some unexpected exclusions in the core outcome set, for example mental health symptoms and treatment adherence were quite frequently used in past research [1], but not included in the core outcome set. At the beginning of the paper we described the recent Kings Fund report that suggested generating agreement amongst healthcare professionals, service users, policy makers and researchers is a difficult but imperative task [23]. We also found this, and feel that the small four-item core outcome set represents the only outcomes that are unanimously agreed upon, despite so many outcomes being of upmost importance to service-users and families. This research has further highlighted the importance of shared decision making and service-user and family involvement to all stakeholder groups [38].This consensus study indicates a desire from all groups to monitor levels of service-users satisfaction and involvement in the process. Whilst such outcomes, were excluded in later stages, primarily on the basis of being process variables/outcomes, it does not reduce their

For peer review only

prospective importance in discharge interventions or provision of care at discharge. The five most agreed upon elements of service-user involvement and satisfaction in discharge were: Service user involvement in discharge planning; Service user understanding of discharge plan; Service user involvement in decision making; Service user satisfaction with information provision at discharge; Service user knowledge of how to access community support. We recommend that future policy makers and healthcare management consider incorporating all of these elements into local level initiatives as overriding principles of care rather than interventions/measures, if they are ever missing from current provision. Furthermore, work highlighting the importance of involving service users in mental health care planning is beginning to emerge, along with measures of such activity. Therefore, we suggest that future research wherever possible should include a service user reported outcome measure of involvement alongside the 4-item core outcome set and any other chosen measures. This could be measured in an existing instrument of service-user involvement care planning in mental health, such as the EQUIP PROM (Patient Reported Outcome Measure) [38], or use the 6 outcomes presented above to develop a self-reported Likert measure of service user involvement specifically in discharge planning, as these 6 items are developed from synthesis of academic literature, qualitative questionnaires and met criteria for consensus amongst experts, so from a psychometric perspective would arguably meet initial face and content validity criteria [39], see additional file 5.

The difficulties of developing a mental health core outcome set was further epitomised when applied to care transitions: a service-level (rather than specific clinical population) multi-agency, multi-stage, complex period of the care pathway [3,24]. Generating a set of meaningful applicable outcomes that span primary and secondary care, across multiple physical locations, that are relevant for every service user was imperative. For example, a great deal of past literature focuses on housing interventions [40–42], and whilst housing is a significant safety issue at discharge, it's not necessarily relevant to all service users. This multi-agency, multi-morbidity complexity was arguably one factor that resulted in the small set of generic outcomes, that arguably differs from narrowly defined clinical core outcome set reported in past literature of many more outcomes [43,44].

This study had several strengths. Our method is based on recommendations from an international panel of experts [25]. Inclusion of service-users and health-care professionals at every stage ensured that outcomes in the final core set embody shared priorities. The comprehensive and laborious long-list process ensured all potential outcomes were considered in the course of the consensus process. However, there were some limitations to our study. The research was only conducted in English, due to budgetary constraints, although our online rounds included participants from 12 countries. Furthermore, in many consensus meetings additional outcomes are often added, the method infrequently serves as means of reducing the number of outcomes included in the preliminary core outcome set from the Delphi [30]. However, in our case we found that the group did not agree with many of the outcomes and it was reduced to a very small COS of 4 items. This is beneficial in some ways, as we hope it is easier for researchers to operationalise a four-item core outcome set. However, to ensure that the spectrum of perceptions of safety by each group is addressed we recommend using the COS alongside an additional service user reported outcome measure (PROM) that encompasses the other outcomes that had high consensus in the Delphi.

The use of outcomes in mental health research and service, is becoming more contested in terms of what is meaningful and effective, it could be argued that core outcomes sets are less

applicable to mental health populations than general health populations, given the complexity of mental health problems and the subjectivity of measuring it. However, as core outcomes sets are relatively uncommon in mental health, we believe (similar to other clinical populations) a small, agreed, feasible set of core outcomes will facilitate between study comparability and advancement in evidence collection [21,25].

Future Directions

Development of this core outcome set involved participation of stakeholders from 12 different countries; (primarily researchers) however, we recommend that further work should be undertaken to validate this core outcome set more widely, particularly in non-English speaking populations. The two of the final four outcomes and many of the preliminary 15 outcomes to emerge from the Delphi, are not necessarily specific to mental health care transitions. Some outcomes are comparable to a similar core outcome set for care transitions of adolescents and young adults with special healthcare needs [45]. Future research may consider a 'transitions of care' core outcome set, to reduce the number of similar core outcome sets.

Another key priority to make this core outcome set operationalised is to agree upon measurement criteria using the COSMIN guidelines [46]. We conducted some preliminary questionnaires with the Delphi panel to produce preliminary measurement recommendations, however there was very little agreement amongst panellists (see additional file 6). Another key priority to make this core outcome set operationalised is to agree upon measurement criteria using the COSMIN guidelines [46]. We conducted some preliminary questionnaires with the Delphi panel to produce preliminary measurement recommendations, however there was very little agreement amongst panellists (see additional file 6). The recommended measures by the panel were Kessler Psychological Distress (K10) and Recovery Quality of Life (ReQoL) within one month of discharge [47,48]. Readmission and suicide completed rates were recommended to be captured within 28 days of discharge using retrospective review of administrative data. However, these are only preliminary recommendations and we highly recommend a future study following COSMIN guidelines.

Conclusion

The four outcomes included in our outcome set represent the consensus opinion of a group of service-users, health-care professionals, and international researchers and addresses an unmet necessity: assisting researchers in the design, implementation and reporting of interventions that aim to improve discharge from acute mental health settings. Ultimately, application of this core outcome set will enhance the relevance of future interventions to health-care professionals, the research community and service-users. If used, the core outcome set could provide more evidenced-based interventions, underpinned by theory of change outlining the relationships between the component of the intervention and the outcome it should improve[1,49]; which should increase service-user safety at this distressing time period.

Patients' consent and permission to publish

All participants gave informed consent to be involved in the study ahead of data collection.

Author Contributions

For peer review only

NT conceived the design of the study; conducted the literature search, meta-synthesis and Delphi; analysed the data; and drafted the majority of the manuscript. JW and NW contributed equally and provided oversight of the study design, analysed and synthesised the data and contributed significantly to the drafting of the manuscript. AG analysed and synthesised data, provided an expert by lived experience opinion on decisions made in regards to wording, study design, PPI involvement, and also contributed adaptations to the manuscript.

Conflicts of Interest

The authors report no conflicts of interest

Ethics Committee Approval

This research received a favourable ethics opinion from Nottingham University Business School Ethics Committee.

Role of Funding Source

This research is funded by the NIHR Greater Manchester Patient Safety Translational Research Centre, they had no role in the writing of manuscript or decision to submit.

Data Statement

Data is available upon reasonable request

Acknowledgements

We would like to thank the patient and public involvement group for their help in designing the questionnaires.

Figure 1 Caption: Process of reducing the outcomes to a set of four core outcomes

Word Count: 5250

References

[1] Tyler N, Wright N, Waring J. Interventions to improve discharge from acute adult mental health inpatient care to the community: systematic review and narrative synthesis. BMC Health Serv Res 2019;19:883. https://doi.org/10.1186/s12913-019-4658-0.

[2] Waring J, McDonald R, Harrison S. Safety and complexity: Inter-departmental relationships as a threat to patient safety in the operating department. J Heal Organ Manag 2006. https://doi.org/10.1108/14777260610662753.

[3] Wright N, Rowley E, Chopra A, Gregoriou K, Waring J. From admission to discharge in mental health services: a qualitative analysis of service user involvement. Heal Expect 2016;19:367–76. https://doi.org/10.1111/hex.12361.

[4] Walter F, Carr MJ, Mok PLH, Astrup A, Antonsen S, Pedersen CB, et al. Premature Mortality Among Patients Recently Discharged From Their First Inpatient Psychiatric

For peer review only

Treatment. JAMA Psychiatry 2017;74:485–92. https://doi.org/10.1001/jamapsychiatry.2017.0071.
[5] Burns J, Flynn S, Lowe R, Turnbull P, Baird A, Stones P, et al. The National Confidential Inquiry into Suicide and Homicide by People with Mental Illness. 2017.
[6] Abraham O, Myers MN, Brothers AL, Montgomery J, Norman BA, Fabian T. Assessing need for pharmacist involvement to improve care coordination for patients on LAI antipsychotics transitioning from hospital to home: A work system approach. Res Social Adm Pharm 2017;13:1004–13. https://doi.org/https://dx.doi.org/10.1016/j.sapharm.2017.02.006.
[7] Exbrayat S, Coudrot C, Gourdon X, Gay A, Sevos J, Pellet J, et al. Effect of telephone follow-up on repeated suicide attempt in patients discharged from an emergency psychiatry department: a controlled study. BMC Psychiatry 2017;17. https://doi.org/10.1186/s12888-017-1258-6.
[8] Vigod SN, Kurdyak PA, Dennis C-L, Leszcz T, Taylor VH, Blumberger DM, et al. Transitional interventions to reduce early psychiatric readmissions in adults: systematic review. Br J Psychiatry 2013;202:187–94. https://doi.org/10.1192/bjp.bp.112.115030.
[9] Waring J, Bishop S, Marshall F, Tyler N, Vickers R. An ethnographic study comparing approaches to inter-professional knowledge sharing and learning in discharge planning and care transitions. J Heal Organ Manag 2019.
[10] Walker SA, Naji SA, Howie FL, Andrew J, Cameron IM, Eagles JM. The effectiveness of psychiatric discharge coordinators. Prim Care Psychiatry 2000. https://doi.org/10.1185/135525700543370.
[11] Bennewith O, Evans J, Donovan J, Paramasivan S, Owen-Smith A, Hollingworth W, et al. A Contact-Based Intervention for People Recently Discharged from Inpatient Psychiatric Care: A Pilot Study. Arch Suicide Res 2014. https://doi.org/10.1080/13811118.2013.838196.
[12] D'souza R. Improving Treatment Adherence and Longitudinal Outcomes in Patients with a Serious Mental Illness by Using Telemedicine. J Telemed Telecare 2002. https://doi.org/10.1177/1357633X020080S251.
[13] Exbrayat S, Coudrot C, Gourdon X, Gay A, Sevos J, Pellet J, et al. Effect of telephone follow-up on repeated suicide attempt in patients discharged from an emergency psychiatry department: A controlled study. BMC Psychiatry 2017. https://doi.org/10.1186/s12888-017-1258-6.
[14] Forchuk C, Martin ML, Chan YL, Jensen E. Therapeutic relationships: From psychiatric hospital to community. J Psychiatr Ment Health Nurs 2005;12:556–64. https://doi.org/10.1111/j.1365-2850.2005.00873.x.
[15] Juven-Wetzler A, Bar-Ziv D, Cwikel-Hamzany S, Abudy A, Peri N, Zohar J. A pilot study of the " Continuation of Care" model in " revolving-door" patients. Eur Psychiatry 2012;27:229–33. https://doi.org/10.1016/j.eurpsy.2011.05.003.
[16] Jensen E, Chapman P, Davis A, Forchuk C, Seymour B, Witcher P, et al. An Evaluation of Community-Based Discharge Planning in Acute Mental Health Care. Can J Community Ment Heal 2010;29:111–24. https://doi.org/10.7870/cjcmh-2010-0038.
[17] Shaffer SL, Hutchison SL, Ayers AM, Goldberg RW, Herman D, Duch DA, et al. Brief critical time intervention to reduce psychiatric rehospitalization. Psychiatr Serv 2015;66:1155–61. https://doi.org/http://dx.doi.org/10.1176/appi.ps.201400362.
[18] Tomita A, Herman DB. The Impact of Critical Time Intervention in Reducing Psychiatric Rehospitalization After Hospital Discharge. Psychiatr Serv 2012. https://doi.org/10.1176/appi.ps.201100468.

For peer review only - http://bmjopen.bmj.com/site/about/guidelines.xhtml

[19] Steffen S, Kösters M, Becker T, Puschner B. Discharge planning in mental health care: A systematic review of the recent literature. Acta Psychiatr Scand 2009;120:1–9. https://doi.org/10.1111/j.1600-0447.2009.01373.x.
[20] Steffen S, Kösters M, Becker T, Puschner B. Discharge planning in mental health care: A systematic review of the recent literature. Acta Psychiatr Scand 2009;120:1–9. https://doi.org/http://dx.doi.org/10.1111/j.1600-0447.2009.01373.x.
[21] Williamson PR, Altman DG, Bagley H, Barnes KL, Blazeby JM, Brookes ST, et al. The COMET Handbook: Version 1.0. Trials 2017. https://doi.org/10.1186/s13063-017-1978-4.
[22] Williamson PR, Altman DG, Blazeby JM, Clarke M, Devane D, Gargon E, et al. Developing core outcome sets for clinical trials: Issues to consider. Trials 2012. https://doi.org/10.1186/1745-6215-13-132.
[23] Lang K. Outcomes for mental health services: what really matters? | HSR UK. 2019.
[24] Waring J, Marshall F, Bishop S, Sahota O, Walker M, Currie G, et al. An ethnographic study of knowledge sharing across the boundaries between care processes, services and organisations: the contributions to 'safe' hospital discharge. Heal Serv Deliv Res 2014. https://doi.org/10.3310/hsdr02290.
[25] Kirkham JJ, Davis K, Altman DG, Blazeby JM, Clarke M, Tunis S, et al. Core Outcome Set-STAndards for Development: The COS-STAD recommendations. PLoS Med 2017. https://doi.org/10.1371/journal.pmed.1002447.
[26] Pedersen ER, Helmuth ED, Marshall GN, Schell TL, PunKay M, Kurz J. Using Facebook to Recruit Young Adult Veterans: Online Mental Health Research. JMIR Res Protoc 2015. https://doi.org/10.2196/resprot.3996.
[27] Contributed Talks. J Evid Based Med 2019;12:24–7. https://doi.org/10.1111/jebm.12336.
[28] Thomas J, Harden A. Methods for the thematic synthesis of qualitative research in systematic reviews. BMC Med Res Methodol 2008;8:45. https://doi.org/10.1186/1471-2288-8-45.
[29] Qualtrics. Qualtrics: The World's Leading Research & Insights Platform. Qualtrics 2016. https://doi.org/10.3109/03639045.2013.790903.
[30] Preston CC, Colman AM. Optimal number of response categories in rating scales: Reliability, validity, discriminating power, and respondent preferences. Acta Psychol (Amst) 2000;104:1–15. https://doi.org/10.1016/S0001-6918(99)00050-5.
[31] Hsu C, Sandford B. The delphi technique: making sense of consensus. Pract Assessment, Res Eval 2007;12:1–8. https://doi.org/10.1016/S0169-2070(99)00018-7.
[32] Bell BG, Spencer R, Avery AJ, Campbell SM. Tools for measuring patient safety in primary care settings using the RAND/UCLA appropriateness method. BMC Fam Pract 2014;15:110. https://doi.org/10.1186/1471-2296-15-110.
[33] Avery AJ, Dex GM, Mulvaney C, Serumaga B, Spencer R, Lester HE, et al. Development of prescribing-safety indicators for GPs using the RAND Appropriateness Method. Br J Gen Pract 2011;61:e526–36. https://doi.org/10.3399/bjgp11X588501.
[34] Kirkham JJ, Gorst S, Altman DG, Blazeby J, Clarke M, Devane D, et al. COS-STAR: A reporting guideline for studies developing core outcome sets (protocol). Trials 2015. https://doi.org/10.1186/s13063-015-0913-9.
[35] Walter F, Carr MJ, Mok PLH, Antonsen S, Pedersen CB, Shaw J, et al. Suicide Methods and Specific Types of Accidental Death and Fatal Poisoning Among Discharged Psychiatric Patients: A National Cohort Study. J Clin Psychiatry 2018;79. https://doi.org/10.4088/JCP.17m11809.
[36] Ali AM, Bottle A. How do we interpret readmission rates? BMJ 2018.

For peer review only

https://doi.org/10.1136/bmj.k3104.
[37] Tomita A, Lukens EP, Herman DB. Mediation analysis of critical time intervention for persons living with serious mental illnesses: assessing the role of family relations in reducing psychiatric rehospitalization. Psychiatr Rehabil J 2014;37:4–10. https://doi.org/10.1037/prj0000015.
[38] Lovell K, Bee P, Brooks H, Cahoon P, Callaghan P, Carter L-A, et al. Embedding shared decision-making in the care of patients with severe and enduring mental health problems: The EQUIP pragmatic cluster randomised trial. PLoS One 2018;13:e0201533. https://doi.org/10.1371/journal.pone.0201533.
[39] Falzarano M, health GZ-J of allied, 2013 undefined. Seeking consensus through the use of the Delphi technique in health sciences research. IngentaconnectCom n.d.
[40] Forchuk C, Russell G, Kingston-Macclure S, Turner K, Dill S. From psychiatric ward to the streets and shelters. J Psychiatr Ment Health Nurs 2006;13:301–8. https://doi.org/http://dx.doi.org/10.1111/j.1365-2850.2006.00954.x.
[41] Forchuk C, Godin M, Hoch JS, Kingston-MacClure S, Jeng M (Mo) S, Puddy L, et al. Preventing Homelessness After Discharge from Psychiatric Wards: Perspectives of Consumers and Staff. J Psychosoc Nurs Ment Health Serv 2013;51:24–31. https://doi.org/10.3928/02793695-20130130-02.
[42] Tomita A, Herman DB. The Role of a Critical Time Intervention on the Experience of Continuity of Care Among Persons With Severe Mental Illness After Hospital Discharge. J Nerv Ment Dis 2015;203:65–70. https://doi.org/10.1097/NMD.0000000000000224.
[43] Fish R, Sanders C, Adams R, Brewer J, Brookes ST, DeNardo J, et al. A core outcome set for clinical trials of chemoradiotherapy interventions for anal cancer (CORMAC): a patient and health-care professional consensus. Lancet Gastroenterol Hepatol 2018. https://doi.org/10.1016/S2468-1253(18)30264-4.
[44] Boers M, Brooks P, Simon LS, Strand V, Tugwell P. OMERACT: An international initiative to improve outcome measurement in rheumatology. Clin Exp Rheumatol 2005;23. https://doi.org/10.1186/1745-6215-8-38.
[45] Fair C, Cuttance J, Sharma N, Maslow G, Wiener L, Betz C, et al. International and Interdisciplinary Identification of Health Care Transition Outcomes. JAMA Pediatr 2016;170:205. https://doi.org/10.1001/jamapediatrics.2015.3168.
[46] Mokkink LB, Terwee CB, Knol DL, Stratford PW, Alonso J, Patrick DL, et al. The COSMIN checklist for evaluating the methodological quality of studies on measurement properties: A clarification of its content. BMC Med Res Methodol 2010;10. https://doi.org/10.1186/1471-2288-10-22.
[47] Keetharuth AD, Brazier J, Connell J, Bjorner JB, Carlton J, Buck ET, et al. Recovering Quality of Life (ReQoL): A new generic self-reported outcome measure for use with people experiencing mental health difficulties. Br J Psychiatry 2018. https://doi.org/10.1192/bjp.2017.10.
[48] Andrews G, Slade T. Interpreting scores on the Kessler Psychological Distress Scale (K10). Aust N Z J Public Health 2001. https://doi.org/10.1111/j.1467-842X.2001.tb00310.x.
[49] Davidoff F, Dixon-Woods M, Leviton L, Michie S. Demystifying theory and its use in improvement. BMJ Qual Saf 2015;24:228–38. https://doi.org/10.1136/bmjqs-2014-003627.

For peer review only

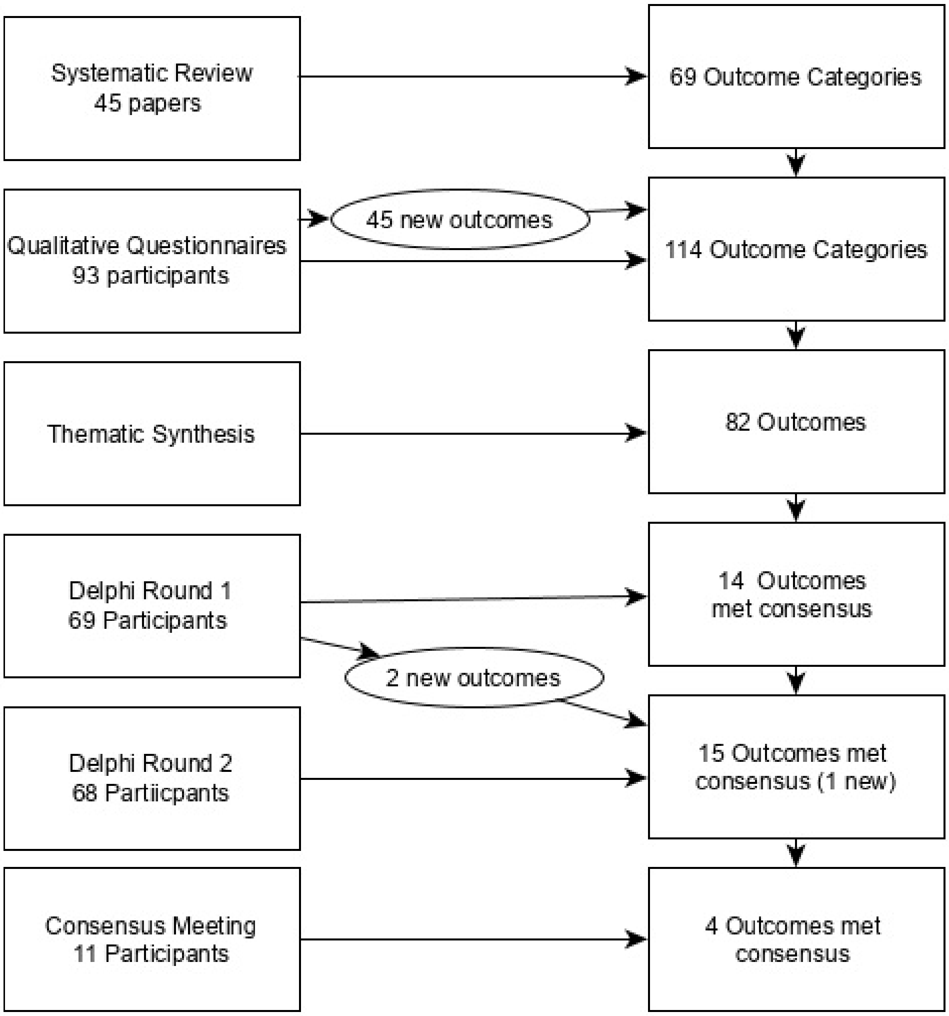

Process of reducing the outcomes to a set of four core outcomes

133x142mm (96 x 96 DPI)

Supplementary Material

File 1: Table of questions asked in qualitative questionnaire

Group	Question
All Participants	What would make discharge from an acute mental health ward **safe** in your opinion?
All Participants	What would make discharge from an acute mental health ward **effective** in your opinion?
All Participants	What would you like to see prioritised for research into discharge from an acute ward?
All Participants	Can you think of any important outcomes to measure in research assessing discharge interventions?
Service User	What do you think is the most difficult aspect of discharge from a mental health acute ward?
Service User	What might improve discharge from a mental health ward?
Service User	Was there any one person/group/intervention which really made a difference for you? If so what was it? What made it so powerful?
Service User	Do you have any other feelings or concerns not addressed in previous questions?
Family member/carer	What do you consider to be the most difficult components of discharge for your family member/friend from an acute mental health ward?
Family member/carer	What types of improvement would you expect to see in your family member/friend following a successful discharge from an acute mental health ward?
Mental health professional	What do you consider to be the most difficult aspects of discharge from an acute unit for your service-users?
Mental health professional	Are there any changes/behaviours you would expect to see in a service-user during or following a safe and/or effective discharge?
Researchers	If applicable, what outcomes did you measure in past research (of discharge interventions)?
Researchers	Are there any concepts that you think are important to measure, but chose not to, due to not having a suitable measurement instrument?
End users of research	If you were looking to use research to inform changes to the discharge procedure within your professional role, what outcomes would you like to see reported?
End users of research	In your opinion, what measurements would persuade you that a discharge intervention is effective?

For peer review only

File 2: Results of Meta-synthesis of Outcomes and data from questionnaire

Clinical

From Review	Additional from Meta-synthesis
Reoccurrence (i.e. Relapse)	Stability
Clinical Recovery	Physical Health and Wellbeing
Personal Recovery	Iatrogenic harm (patient safety incidents- harm caused by the care system)
Substance Misuse (inc. alcohol)	Serious accidental injury
Self-injury (i.e. harm to self)	Mortality
Suicide Risk	Mental health and illness (symptoms/psychological distress)
Suicidal Ideation	
Suicide Attempted	
Suicide Completed	
Medication Knowledge	
Medication Adherence	
Medication Side Effects	
Medication Management	
Global Functioning	

Service

From Review	Additional from Meta-synthesis
Emergency department visits post discharge	Police intervention post discharge
Readmission	Engagement with psychological intervention
Length of stay pre-discharge	Engagement with community services
Length of stay post-discharge (i.e. subsequent readmissions)	Availability of appropriate community support (i.e. allocated key worker)
Outpatient appointment/visit adherence	Length of time before follow up/aftercare
Service use	Leaving the hospital against medical advice
	Service user involvement in decision making (shared decision making)
	Serious incidents (i.e. serious incidents reported formally)

Satisfaction

From Review	Additional from Meta-synthesis
Service user expectations of care	Professionals/care teams satisfaction with information provision at discharge
Service user satisfaction with discharge	Service user satisfaction with information provision at discharge (e.g. regarding medication, risk, crisis planning)
Service user experience of discharge	
Service user satisfaction with treatment	
Carer/family/other satisfaction with discharge	
Staff satisfaction with discharge	

Personal

From Review	Additional from Meta-synthesis
Hopelessness	Feelings of safety
Boredom	Feelings of support (from various groups- i.e. family, professionals and friends)
Loneliness	Experience of stigma
Isolation	Quality of interpersonal relationships (friends and family)
Coping skills	Self-management
Concern/anxiety about discharge	
Autonomy (e.g. independence, autonomous decision making)	

For peer review only

Housing Stability	
Discharge to appropriate accommodation	
Service user knowledge of own condition	
Quality of life	
Community participation	
Violence	
Aggression	
Victim of crime	
Stable relationships with health professionals	
Employment	

Discharge Planning

From Review	Additional from Meta-synthesis
	Service user involvement in discharge planning
	Family/carer involvement in discharge planning
	Advocate involvement in discharge planning
	Completion of planned care
	Continuity of contact
	Service user readiness for discharge (incl. preparation)
	Clinical readiness for discharge
	Experience of coercion at discharge (e.g. feeling forced to leave)
	Provision of financial support (e.g. benefits advice, debt advice)
	Delays in expected discharge
	Service availability (e.g. availability of community services, CPN, follow-up, social worker, bed in residential service)
	Family/carer/other readiness for discharge
	Staff understanding of safe and effective discharge
	Plan for community engagement
	Is there a plan for care post discharge created
	Has the plan for care post-discharge been communicated with patient
	Has information about the discharge been reported to other relevant services

Changes/Combinations

Outcomes from Review	Changes/Combinations
Social Recovery	Combined into quality of interpersonal relationships
Relapse	PPI suggestion change to reoccurrence
Self-harm	PPI suggestion change to self-injury
Depression	Combined into symptoms
Anxiety	Combined into symptoms
Illegal drug use	Combined into substance misuse
Alochol use	Combined into substance misuse
Addiction severity	Combined into substance misuse
Crisis planning	Combined into information provision
Risk communication	Combined into information provision
Allocated worker	Combined into availability of appropriate community support
Better knowledge transfer	Combined into information provision (professional)
Therapeutic alliance	Combined into stable relationships with HPs
Contact with ambulatory care	Combined into emergency visits
Treatment adherence	Combined into medication adherence and engagement with psychological services
7 day follow up	Changed to length of time before follow up for global audience
Psychological distress	Combined into symptoms
Family relations	Combined into quality of interpersonal relationships
Community integration and functioning	Combined into Community participation

Outcomes Removed from Process

Outcomes from Review	Reason
Antipsychotic Politherapy	Too specific
Autistic life	Too specific

For peer review only

Medication Problems	Too vague
Functional Recovery	Meaning not explicit
Satisfaction with service in community	Not relevant for this COS
Global satisfaction with discharge	Meaning not explicit
Quality of care	Too vague
Treatment decision making	Not relevant for this COS
Financial cost to provider	Impossible to measure
Number of beds	Too vague
Staff workload	Too vague
Freedom	Too vague
Self-activity	Too vague

Carer outcomes

**Outcomes from Review**	**Decision**
Caregiver Burden	Yes
Caregiver Health Status	No (too specific)
Caregiver Knowledge about illness	Yes
**From Synthesis**	
Carer support	No
Relationship between family and SU	No (probably a variable)

For peer review only

File 3: Participant Demographics

**Categories**											
**Group**	**Service Users**	**Families and Carers**	**Healthcare Professionals**	**Researchers**	**End-users of Research**						
**n**	27	17	39	37	15						
**Gender**	**Male**	**Female**	**Other**								
**n**	28	63	0								
**Age**	**18-24**	**25-34**	**35-44**	**45-54**	**55-64**	**65-74**	**75-84**				
**n**	4	20	30	24	10	4	1				
**Location**	**International**	**East of England**	**East Midlands**	**London**	**North West**	**North East**	**South East**	**South West**	**Wales**	**West Midlands**	**Yorkshire and the Humber**
**n**	24	2	11	7	16	1	6	5	0	13	7

Locations for International participants

**Country**	**N**
Australia	4
Canada	2
China	1
France	1
Germany	1
Iran	1
Italy	1
Northern Ireland	1
South Africa	1
Switzerland	4
USA	7

For peer review only

File 4: Full list of Delphi outcomes, consensus levels, and round of inclusion/exclusion

	R1 Percentage agreement	R1 Percentage Disagreement	Median	Researchers	Sus and carers	HCPs and DMs	Round 2 Status	Round 2	Researcher	Sus and carers	HCP and DMs	Median
Service user involvement in discharge planning (inc. feeling listened to)	87%	4%	7	65%	100%	95%	Include					
Functioning (health, social, etc.)	83%	3%	6	69%	100%	81%	Include					
Mental health and illness (symptom/psychological distress)	83%	3%	6	73%	91%	86%	Include					
Personal Recovery	82%	1%	6	75%	86%	86%	Include					
service user understanding of discharge plan	81%	3%	6	65%	91%	86%	Include					
Quality of life	81%	1%	6.5	65%	90%	86%	Include					
suicide Completed	80%	4%	7	80%	90%	68%	Include					
Readmission	80%	6%	6	77%	77%	86%	Include					
service user involvement in decision making (shared decision making)	77%	4%	7	50%	95%	86%	Include					
service user satisfaction with information provision at discharge (e.g. regarding medication, risk, crisis planning)	77%	6%	6	65%	86%	81%	Include					
service user knowledge of how to access community support (i.e. in an emergency)	77%	3%	6	58%	91%	86%	Include					
Recurrence (i.e. relapse)	75%	1%	6	58%	91%	76%	Include					
Suicide Attempted	75%	4%	6	62%	86%	81%	Include					
discharge to appropriate accommodation	75%	3%	6	69%	91%	67%	Include					
service user satisfaction with treatment	74%	7%	6	58%	91%	71%	Re-present	59%	43%	67%	70%	6
service Availability (e.g. availability of community service, CPN, follow-up, social worker,	74%	7%	6	46%	86%	95%	Re-present	45%	27%	67%	50%	5

For peer review only

bed in residential service)												
Existence of a plan for community engagement/ care post discharge	74%	4%	6	58%	77%	90%	Re-present	72%	57%	83%	90%	6
Feeling of support (from various group i.e. family, professional, friends)	72%	4%	6	65%	77%	71%	Re-present	43%	33%	50%	50%	5
Suicide Risk	71%	3%	6	58%	77%	81%	Re-present	42%	43%	56%	25%	5
Family/carer/other involvement in discharge planning (inc. feeling listened too)	71%	6%	6	65%	77%	75%	Re-present	58%	50%	67%	65%	6
service user readiness for discharge (incl. preparation)	71%	6%	6	58%	77%	81%	Re-present	35%	27%	50%	30%	5
Feeling of safety	70%	7%	6	65%	73%	71%	Re-present	36%	37%	56%	25%	5
service user satisfaction with discharge	69%	4%	6	50%	76%	86%	Re-present	45%	40%	50%	50%	5
Physical Health and Wellbeing	68%	3%	6	50%	77%	81%	Re-present	49%	43%	61%	45%	5
service user experience of discharge	68%	10%	6	46%	86%	76%	Re-present	29%	23%	44%	30%	5
Housing stability	68%	3%	6	62%	77%	67%	Re-present	41%	37%	39%	40%	5
Coping skills	68%	6%	6	50%	77%	80%	Re-present	39%	40%	50%	35%	5
Continuity of contact	68%	6%	6	64%	77%	62%	Re-present	65%	63%	72%	60%	6
Mortality	67%	7%	6	68%	76%	57%	Re-present	42%	43%	50%	30%	5
self management	67%	4%	6	46%	86%	71%	Re-present	29%	23%	39%	20%	5
Length of time before follow up/aftercare	65%	6%	6	46%	77%	76%	Re-present	58%	43%	83%	65%	6
Isolation	65%	9%	6	46%	73%	81%	Re-present	55%	50%	72%	45%	6
Availability of appropriate community support (i.e. allocated key worker)	64%	9%	6	38%	82%	76%	Re-present	52%	27%	72%	70%	6
service user knowledge of own condition	64%	6%	6	42%	91%	62%	Re-present	36%	30%	50%	35%	4
service user expectation of care	63%	3%	6	35%	76%	81%	Re-present	28%	20%	28%	40%	4
Hopelessness	63%	9%	6	54%	67%	71%	Re-present	46%	47%	44%	45%	5
suicidal Ideation	62%	7%	6	46%	77%	67%	Re-present	51%	50%	61%	45%	6

For peer review only

Medication Adherence	62%	4%	6	50%	82%	52%	Re-present	45%	47%	44%	40%	5
Loneliness	62%	10%	6	42%	68%	81%	Re-present	41%	37%	56%	30%	5
Information about the discharge reported to other relevant service	62%	9%	6	50%	73%	71%	Re-present	48%	37%	78%	40%	5
service user experience of coercion at discharge	61%	6%	6	46%	77%	63%	Re-present	43%	40%	67%	35%	5
Clinical readiness for discharge	60%	6%	6	48%	67%	67%	Re-present	36%	30%	39%	40%	5
Medication side Effects	59%	7%	6	38%	77%	67%	Re-present	41%	30%	61%	35%	5
Emergency department visit post discharge	59%	6%	6	58%	68%	52%	Re-present	62%	63%	67%	60%	6
Quality of interpersonal relationship (friend and family)	59%	7%	6	50%	77%	52%	Re-present	46%	40%	72%	35%	5
Iatrogenic harm (patient safety incident or harm caused by the care system)	59%	4%	6	40%	64%	71%	Re-present	51%	37%	72%	50%	6
Completion of planned care	59%	7%	6	50%	76%	52%	Re-present	39%	40%	56%	30%	5
Concern/anxiety about discharge	58%	9%	6	31%	73%	76%	Re-present	26%	17%	33%	35%	4
Autonomy (e.g. independence, autonomous decision making)	57%	3%	6	35%	73%	70%	Re-present	36%	27%	50%	30%	5
staff understanding of safe and effective discharge	57%	10%	6	40%	68%	67%	Re-present	41%	30%	67%	35%	4
self injury (i.e. harm to self)	57%	6%	6	50%	64%	52%	Re-present	49%	53%	61%	35%	5
Financial support (e.g. benefit advice, debt advice)	57%	9%	6	38%	82%	57%	Re-present	46%	40%	72%	30%	5
stable relationship with health professional	55%	4%	6	38%	71%	58%	Re-present	33%	37%	50%	20%	5
Medication Management	54%	3%	6	42%	71%	52%	Re-present	42%	37%	61%	40%	5
Carer/family/other satisfaction with discharge	54%	9%	6	35%	68%	57%	Re-present	36%	27%	61%	35%	4
Clinical Recovery	53%	7%	6	60%	55%	43%	Re-present	36%	33%	50%	30%	5
service use	52%	4%	6	46%	50%	63%	Re-present	30%	33%	39%	15%	4
Violence	52%	9%	6	46%	60%	48%	Re-present	29%	33%	33%	15%	4
Victim of crime	52%	12%	6	38%	68%	48%	Re-present	28%	30%	44%	10%	4
Experience of stigma	52%	7%	6	42%	59%	57%	Re-present	30%	20%	50%	25%	4

For peer review only

Engagement with psychological intervention	51%	4%	6	35%	45%	76%	Re-present	33%	30%	67%	5%	4
Cancellation or change to follow up meeting	51%	12%	6	19%	82%	57%	Re-present	42%	23%	67%	40%	5
serious incident (i.e. serious incident reported formally)	50%	6%	5.5	44%	45%	62%	Exclude					
Employment	50%	7%	5.5	58%	48%	38%	Exclude					
Medication Knowledge	49%	9%	5	35%	64%	52%	Exclude					
Engagement with community service	49%	4%	5	35%	50%	71%	Exclude					
Length of stay pre-discharge	46%	10%	5	38%	55%	48%	Exclude					
Family/carer/other readiness for discharge	46%	9%	5	42%	55%	43%	Exclude					
Leaving the hospital against medical advice	45%	14%	5	46%	55%	33%	Exclude					
Outpatient appointment/visit adherence	44%	4%	5	54%	57%	24%	Exclude					
Aggression	44%	10%	5	42%	43%	48%	Exclude					
Delay in expected discharge	43%	13%	5	12%	67%	57%	Exclude					
substance Misuse (incl. alcohol)	41%	9%	5	28%	50%	48%	Exclude					
serious accidental injury	40%	12%	5	25%	36%	62%	Exclude					
Community participation	39%	6%	5	35%	36%	48%	Exclude					
stability	38%	4%	5	13%	59%	47%	Exclude					
Advocate involvement in discharge planning	37%	12%	5	24%	45%	45%	Exclude					
Professional/care team satisfaction with information provision at discharge	36%	13%	5	19%	45%	48%	Exclude					
Primary Care/Community service/Nongovernmental or Charity service satisfaction	35%	16%	5	15%	50%	48%	Exclude					
Police intervention post discharge	34%	16%	5	35%	32%	35%	Exclude					
staff satisfaction with discharge	28%	16%	5	19%	36%	29%	Exclude					
Boredom	26%	25%	4	12%	36%	33%	Exclude					
Meaningful Activity (i.e. employment, studying, volunteering)	New outcome proposed in round 1						Include	77%	73%	83.33%	80%	6
Resilience	New outcome proposed in round 1				Do not present							
Dual harm (i.e. a person harms themselves and others)	New outcome proposed in round 1						Exclude	32%	23%	55.56%	30%	4

For peer review only

Annual admissions	New outcome proposed in round 1	Do not present							
Harm to others (violence, criminal or risk-taking behaviour)	New outcome proposed in round 1	Do not present							
Levels of patient confidentiality	New outcome proposed in round 1	Do not present							
Personal learning	New outcome proposed in round 1	Do not present							

For peer review only

File 5: Proposed potential service user discharge experience PROM that includes items of most importance to stakeholders in addition to the core outcome set

Item	Strongly Agree	Agree	Neither Agree nor Disagree	Disagree	Strongly Disagree
1. I felt involved in my discharge planning					
2. I understood my discharge plan					
3. I know how to access support in the community					
4. I was frequently involved in decisions about my care at discharge					
5. I was discharged to appropriate accommodation					
6. I was satisfied with the information provided to me at discharge					

File 6: Outcome Measure Recommendation Results

Methods

After the core outcome set was agreed in the consensus meeting, we invited all participants from the earlier stages of the project to recommend measures and time markers in a final online questionnaire. Participants were invited to participate if they had been involved in any of the previous online rounds. The invitation made it clear that the questionnaire is most relevant to researchers, but that other groups with an opinion or interest are welcome to contribute. This was due to the specific knowledge of instruments required to complete this round.

In this questionnaire participants were presented with the four core outcomes. For each core outcome they were presented with any measures used to assess that outcome in our systematic review studies [1] and any additional measures that had been recommended to the team during the process. Participants were asked to choose the one most appropriate, (don't know, other, new instrument, no instrument were also options). A second question also asked which time markers would be recommended, with options to select all applicable. These options were also developed based on time markers used in the systematic review [1].

Results

Forty-three of the 93 invited participants responded (15 service users, 8 family members/carers, 23 researchers, 10 healthcare professionals, 3 end users of research), although as in previous rounds these were not distinct categories. Fifty-three percent of the respondents were researchers, this was expected as in the email we suggested that this stage may be more meaningful or of interest to this group, but as a team we chose not to exclude other groups with opinions on measurement instruments. Twenty-three percent of participants were international

For peer review only

researchers (from USA, Switzerland, Canada, and Australia). Table 4 shows the preliminary minimum measure recommendations and time markers, additional file 5 shows the results upon which the recommendations were based.

Table 1: Summary of Measurement Recommendations

Core Outcome	Instrument/Measure	Time Marker
Readmission	Retrospective review of administrative data	Within 28 days of discharge
Suicide Completed	Retrospective review of administrative data	Within 28 days of discharge
Psychological Distress	Kessler Psychological Distress (K10)	One month post-discharge
Quality of Life	ReQoL-10	One month post-discharge

1. Readmission

A minimum recommendation of using retrospective review of administrative data for readmissions within a defined time period, the most agreed was 28 days. Participants indicated that routine data collection might cover slightly different time periods. Twenty-six of the 43 participants recommended a measure of around a month (1 month, 30 days or 28 days) with 28 days being this most popular. However, they also advise that this should be supplemented either by cross-checking with service-users, case managers or carers, where possible to improve quality of data. Those looking for more comprehensive data may also like to record 7 days, 3 months and 6 months as these were also popular recommendations.

2. Quality of Life

The participants recommended that researchers use the Recovering Quality of Life (ReQoL-10) at one month post-discharge [35]. This was the most recommended instrument by the group. However, many participants also voted for ReQoL 20, a large proportion of the group suggested this outcome. As this is a quality of life measure specific to mental health recovery they felt this is most appropriate. The one month time marker is in-keeping with the other COS time frames, making a more comprehensive and accessible core outcome set. Those using within-participant measures of quality of life may like to also measure a pre-discharge baseline. Researchers looking for more thorough assessment of quality of life may like to also measure at 7 days post-discharge and 3 months, as these were also highly recommended time markers or use the ReQoL 20 and report both scores for comparability.

3. Suicide Completed

The participants recommended retrospective review of administrative data, for suicide completed within 28 days of discharge. Retrospective review is in line with other outcomes and was marginally the highest suggestion. We chose within 28 days for consistency with readmission data. Researchers looking for more comprehensive data may want to use 7 days and 3 months as these were highly recommended also. They may also want to cross-check this information against other sources (carers/case managers) to ensure it is correct and

For peer review only

reported, particularly as participants mentioned the impact of incorrect coroner's reports on such data.

4. Psychological Distress

The participants recommended Kessler Psychological Distress (K10) one month post-discharge [36] . For consistency with other outcomes we recommend measure at one month. Seven days and 3 months are also highly recommended, so we would recommend these for research that is more robust. Although there were very few votes for instruments for psychological distress and qualitative comments revealed that participants felt this is not measurable. The same amount of people who voted for K10 also voted for interviews or other measures and a similar amount recommended the development of a new measure, CORE-10 was similarly close [37]. Whilst we make this recommendation, we also suggest that future researchers may look to develop something specific for Psychological Distress in this core outcome set. Interviews would not effectively facilitate the between study comparison, the key purpose of a COS.

Outcome 1: Readmission

Measure	Number of votes	Important comments
Interviews with SUs	12	• In some countries… there is no easily accessible data on readmission rates…in our experience self-reported in the most reliable way
Retrospective review of administrative data	13	• Might not show people who need admission but don't because there's no bed
Extracted from case-managers notes and cross-checked with hospital records	10	• Might be easier to gather administrative data, but worth cross-checking to improve quality of information
Self-reported questionnaire	1	
Other- carer interview	2	
Total	38	

Conclusion: A minimum recommendation of using retrospective review of administrative data. This will allow for various studies with diverse time and financial limits to use the COS. However, we also advise that this should supplemented either by cross-checking with service-users, case managers or carers, where possible to improve quality of data.

Time Markers

Time marker	Number of Votes
Within 2 days	3
Within 3 days	2
Within 7 days	17
Within 28 days	12
Within 30 days	7
Within 1 month	7
Within 6 weeks	4
Within 12 weeks	7
Within 3 months	11
Within 6 months	14
Other (1yr and 3yrs)	1

For peer review only

Around 3 days	5
Around a month	26
Around 3 months	18

Conclusion: The minimum recommendation to record readmission within 28 days. 26 of the 43 participants recommended a measure of around a month (1 month, 30 days or 28 days) with 28 being this most popular. Those looking for higher quality or more comprehensive data may also like to record 7 days, 3 months and 6 months as these were also popular recommendations.

Outcome 2: Quality of Life

Instrument	**Number of votes**	**Comments**
ReQol- 10	9	'I think ReQol would be best for patients in MH services as I understand it was validated for CMHT patients but if someone didn't have contact with MH services before and isn't under a CMHT afterwards another measure might be better.'
ReQol- 20	3	
Quality of Life Brief Version (Lehman)	2	
WHO Quality of Life Scale	5	
Manchester Short Assessment Quality of Life (MANSA)	1	
Satisfaction with Life Scale (SWLS)	1	
SF12	2	
EQ5D	0	
AQoL-8D	0	
Other (ICECAP)	1	
Develop a new tool	2	
No instrument (interviews etc. instead)	7	I think the use of tools should be complemented with interviews with service users and carers.
Total	33	
ReQoL combined (10+20)	12	

Conclusion: We recommend that researchers use ReQoL-10. This was the most voted for instrument. If we also combine the scores with those who voted for ReQoL 20, a large proportion of the group suggested this outcome. As this is a quality of life measure specific to mental health recovery we feel this is most appropriate.

Time markers

**Time Point**	**Number of votes**
Within 2 days	3
Within 3 days	2
Within 7 days	16
Within 28 days	11
Within 30 days	6
Within 1 month	9
Within 6 weeks	3
Within 12 weeks	5
Within 3 months	21
Within 6 months	11
Other (within 9 months, 12 months and 3 years)	2
Pre-discharge	9
Around 3 days	5
Around 1 month	26
Around 3 months	26

Conclusion: We recommend a minimum measure of QoL at one month post-discharge in RCTs. This is in keeping with the readmission time frames, making a more comprehensive and accessible core outcome set. Those using within-participant measures of quality of life may like to

For peer review only

also measure a pre-discharge baseline. Those looking for more thorough assessment of QoL may like to also measure at 7 days post-discharge and 3 months, as these were also highly recommended time markers.

Core Outcome 3: Suicide completed

Measure	Number of votes
Retrospective review of administrative data	18
Extracted from case managers and cross-checked with hospital records	17
Extracted from clinical case notes	14
Other<br>Extracted from serious incident reporting<br>From family and friends	4
Total	53

Conclusion: Retrospective review of administrative data. To keep in line with other outcomes and was marginally highest measure.

Time Marker	Number of votes
Within 2 days	2
Within 3 days	2
Within 7 days	20
Within 28 days	12
Within 30 days	4
Within 1 month	9
Within 6 weeks	0
Within 12 weeks	4
Within 3 months	14
Within 6 months	13
Other	2
Around 3 days	4
Around 1 month	25
Around 3 months	18

Conclusion. Within 28 days for consistency with other outcomes. Other researchers may want to use 7 days and 3 months as these were highly recommended also.

Outcome 4: Psychological Distress

Instrument	Number of votes
Kessler Psychological Distress (K10)	6
CORE-10	5
Brief Psychiatric Rating Scale (BPRS)	3
Brief Symptom Inventory (BSI)	1
The positive and negative symptom scale	1
The Discharge List (DL) â€“ 20 item scale	2
Clinical Global Impression to Assess illness severity	0
ASI (Psychiatric problem subscale)	0
PSYRAT (Psychological Distress subscale)	1
Develop a new self-assessment tool for psychological distress	5
Other	1
No instrument (interview or other method instead)	6
Total	31

Conclusion: We recommend Kessler Psychological Distress (K10). Although there were very few votes for measures. The same amount of people who voted for K10 also voted for interviews or other measures and a similar amount recommended the development of a new measure,

For peer review only

CORE-10 was similarly close. Whilst we make this recommendation, we also suggest that future researchers may look to develop something specific for this core outcome set. Interviews would not allow for easy comparison of scores so would not be relevant for a core outcome set.

Time Markers

**Time Marker**	**Number of votes**
Within 2 days	2
Within 3 days	4
Within 7 days	22
Within 28 days	8
Within 30 days	4
Within 1 month	14
Within 6 weeks	3
Within 12 weeks	4
Within 3 months	15
Within 6 months	14
Other	3
Pre-discharge baseline measure at ...	5
Around 3 days	6
Around 1 month	26
Around 3 months	19

Conclusion: The minimum recommendation is one month post-discharge. For consistency with other outcomes we recommend measure at one month. 7 days and 3 months are also highly recommended, so we would recommend these for more thorough research.

For peer review only

For peer review only

For peer review only

BMJ Open

Developing a Core Outcome Set for Interventions to Improve Discharge from Mental Health Inpatient Services: A Survey, Delphi and Consensus Meeting with Key Stakeholder Groups.

Journal:	*BMJ Open*
Manuscript ID	bmjopen-2019-034215.R2
Article Type:	Original research
Date Submitted by the Author:	25-Feb-2020

Complete List of Authors:	Tyler, Natasha; The University of Manchester, NIHR Greater Manchester Patient Safety Translational Research Centre<br>Wright, Nicola; University of Nottingham<br>Grundy, Andrew; University of Nottingham<br>Waring, Justin ; University of Birmingham

**Primary Subject Heading**:	Mental health
Secondary Subject Heading:	Health services research
Keywords:	MENTAL HEALTH, Core Outcome Set, Discharge, Care Transitions, Acute Adult Mental Health Services

SCHOLARONE™
Manuscripts

I, the Submitting Author has the right to grant and does grant on behalf of all authors of the Work (as defined in the below author licence), an exclusive licence and/or a non-exclusive licence for contributions from authors who are: i) UK Crown employees; ii) where BMJ has agreed a CC-BY licence shall apply, and/or iii) in accordance with the terms applicable for US Federal Government officers or employees acting as part of their official duties; on a worldwide, perpetual, irrevocable, royalty-free basis to BMJ Publishing Group Ltd ("BMJ") its licensees and where the relevant Journal is co-owned by BMJ to the co-owners of the Journal, to publish the Work in this journal and any other BMJ products and to exploit all rights, as set out in our licence.

The Submitting Author accepts and understands that any supply made under these terms is made by BMJ to the Submitting Author unless you are acting as an employee on behalf of your employer or a postgraduate student of an affiliated institution which is paying any applicable article publishing charge ("APC") for Open Access articles. Where the Submitting Author wishes to make the Work available on an Open Access basis (and intends to pay the relevant APC), the terms of reuse of such Open Access shall be governed by a Creative Commons licence – details of these licences and which Creative Commons licence will apply to this Work are set out in our licence referred to above.

Other than as permitted in any relevant BMJ Author's Self Archiving Policies, I confirm this Work has not been accepted for publication elsewhere, is not being considered for publication elsewhere and does not duplicate material already published. I confirm all authors consent to publication of this Work and authorise the granting of this licence.

For peer review only

Title: Developing a Core Outcome Set for Interventions to Improve Discharge from Mental Health Inpatient Services: A Survey, Delphi and Consensus Meeting with Key Stakeholder Groups.

Authors:

Corresponding Author: Natasha Tyler (Greater Manchester Patient Safety Translational Research Centre, University of Manchester, Manchester, UK, Natasha.tyler@manchester.ac.uk, 0161 275 7620)

Nicola Wright (University of Nottingham, Nottingham, UK)

Andrew Grundy (University of Nottingham, Nottingham, UK)

Justin Waring (Health Services Management Centre, University of Birmingham, Birmingham, UK)

For peer review only

Abstract

Objective

To develop a core set of outcomes to be used in all future studies into discharge from acute mental health services to increase homogeneity of outcome reporting.

Design

We used a cross-sectional online survey with qualitative responses to derive a comprehensive list of outcomes, followed by two online Delphi rounds and a face-to-face consensus meeting.

Setting

The setting the core outcome set applies to is acute adult mental health.

Participants

Participants were from recruited from five stakeholder groups: service-users, families and carers, researchers, healthcare professionals and policy makers.

Interventions

The core outcome set is intended for all interventions that aim to improve discharge from acute mental health services to the community.

Results

Ninety-three participants in total completed the questionnaire, 69 in Delphi round 1, and 68 in round 2, with relatively even representation of groups. Eleven participants attended the consensus meeting. Service-users, healthcare professionals, researchers, carers/families and end-users of research agreed on a four-item core outcome set: Readmission, Suicide completed, Service-user reported psychological distress and Quality of life.

Conclusion

Implementation of the core outcome set in future trials research will provide a framework to achieve standardisation, facilitate selection of outcome measures, allow between-study comparisons, and ultimately enhance the relevance of trial or research findings to healthcare professionals, researchers, policy makers and service users.

Article Summary

Strengths and Limitations

- This is the first initiative to reduce heterogeneity in outcome reporting for interventions that improve discharge from acute mental health services.
- A high level of consensus amongst 69 service users, families/carers, healthcare professionals, researchers and policy makers was achieved.
- COS-STAR reporting guidelines were followed.

For peer review only

- Although the stakeholder group included international researchers, service users and healthcare professionals were recruited only from the UK.
- Not all of the participants who contributed online attended the face-to-face meeting, whereby the core outcome set was reduced considerably.

Funding

This work was funded by the National Institute for Health Research (NIHR) Greater Manchester Patient Safety Translational Research Centre (NIHR Greater Manchester PSTRC). The views expressed are those of the author(s) and not necessarily those of the NIHR or the Department of Health and Social Care.

Competing Interests

The authors report no competing interests.

Key Words

- Acute Adult Mental Health Services
- Core Outcome Set
- Mental Health
- Discharge
- Care Transitions

Background

Care transitions (when patient care is transferred from one team, department or organisation to another) are widely recognised as a vulnerable and high-risk stage in the care pathway [1–3]. Safety issues may be intensified in acute mental health services, where care transitions are described as chaotic [3]. For example, suicide risk increases post-discharge from acute mental health services [4,5]. A growing body of research describes these risks either directly in terms of identified 'safety' events or indirectly in terms of broader 'problems', including for example treatment non-adherence, inappropriate readmissions, increased risk of self-injury or suicide attempts [3,6–8].

Internationally, researchers have attempted to find solutions to the problems or threats to safety associated with discharge from acute mental health services by developing interventions that aim to improve different aspects of discharge planning, transitions, continuity of care, and follow-up care [9]. Some interventions aim to improve discharge by introducing new roles, for example a discharge co-ordinator [10]. Others focus on increasing contact between clinical staff and service users, for example using letters or telephone follow-up [11–13]. Many 'successful' interventions in reducing readmission, bridged the boundaries between ward and community by providing types of ward-based care in the community [14,15] or where community teams lead discharge planning on the wards [16],

For peer review only

There has been little attempt to compare these diverse interventions. Existing reviews have included either a narrow range of studies addressing a single outcome or focus on a specific time frame in an attempt to synthesise results [8,17]. Comparison and meta-synthesis of effectiveness of interventions has reported limited success. Across the papers included in our systematic review and those by other researchers [1,18], variation in the outcomes reported is substantial. This limits between study comparability and delays advancement in evidence collection. Furthermore, outcomes in these trials were not necessarily representative of the measures that service users would consider important at discharge. Both matters can potentially be addressed with the development of a 'core outcome set', defined as "an agreed, standardised collection of outcomes which should be measured and reported, as a minimum, in all trials for a specific clinical area" [19].

The development and use of 'core outcome sets' has been endorsed as a means to reduce outcome heterogeneity in research, and to increase the relevance of research through the involvement of key stakeholders in its development [20]. There is an emerging body of literature highlighting the difficulties of defining and assessing outcomes in a mental health population [21]. There is also evidence of a lack of agreement amongst key groups about what should be measured and in what capacity and an evident tension between the population health perspective and provision of individualised care [17,21]. One aforementioned previous review identified the need for consensus on outcome definitions in discharge planning interventions [17]. Similarly a recent Kings Fund report suggested broader consensus upon the outcomes that matter is imperative for advancement [21]. Therefore, generating agreement amongst healthcare professionals, service users, policy makers and researchers is a difficult but imperative task, to enable the useful direction of healthcare services [21]. The difficulties are further exemplified when applied to care transitions, a multi-agency, multi-stage, complex period of the care pathway [3,22]. This paper outlines the development of a core outcome set for research of interventions to improve discharge from acute mental health wards to the community.

The objective of this study was to obtain international consensus on a set of core outcome measures to be reported in all interventions intended to improve discharge from mental health inpatient services.

Methods

Study overview

The scope of the core outcome set was defined according to the criteria recommended by Core Outcome Measures in Effectiveness Trials (COMET) [23]. The study was prospectively registered with the COMET initiative (1276). The health condition was functional conditions (mental disorders other than dementia, and includes severe mental illness such as schizophrenia). The population was adults aged 18-65, the intervention was any interventions that aimed to improve discharge from an acute mental health setting to the community. The core outcome set was developed using four stages, including service users and healthcare professionals at each stage: (1) a long list of outcomes was generated through a systematic review [1] and qualitative survey; (2) the resulting long outcome list was used to populate an online Delphi process (2 rounds); and (3) the results of the Delphi survey were appraised at a consensus meeting and the final core outcome set was established. The process included a series of core research team meetings at every stage, the team comprised of a

For peer review only

researcher and core outcome set developer, an associate professor in mental health and mental health nurse, a researcher and expert by lived experience of acute services and an expert in patient safety. Participants did not fit into distinct homogeneous groups, for example mental health professionals were sometimes also past service users or family members of service users. Similarly researchers had personal experience of inpatient mental health services. Therefore, wherever possible we considered the group as whole and tried not to compare categories.

Participants

Participants were recruited in a number of ways in December 2018 to January 2019. Academic researchers were recruited if their research had been included in our systematic review or if they were known researchers in the field identified by the team. End-users of research (policy makers, NGOs, NHS management, commissioners, advocates etc.) were recruited via searching for publicly available contact details or using our team's professional networks or social media. Service users and healthcare professionals were recruited through social media. Twitter was nominated as the primary platform for recruitment due to its ability to reach into the specific communities of interest we required: mental health professionals, service users and families/carers. Using social media has been reported as a cost-effective and efficient way to recruit those from potentially stigmatised groups [24]. Further, the peer network structures of social media platforms enable users to recruit other users through sharing links within their networks.

The same participant group was used throughout the iterative research process, therefore, in order to reduce attrition, those who dropped out in early rounds were invited to re-join the panel in subsequent rounds. Participants were recruited for the consensus meeting during the Delphi, UK participants were asked to indicate whether they would be interested in a face-to-face meeting. We invited a random sample of interested participants to attend, that ensured representative of the stakeholder groups. If a participant declined the invite a similarly matched participant was invited from the Delphi panel principally, or the teams wider network.

Stage 1: Gathering information

In addition to the outcomes extracted from the systematic review [1], outcomes of importance to each stakeholder group were identified through qualitative surveys. For the main body of the questionnaire, we used open questions that were developed to elicit potential additional outcomes. The questions were loosely modelled on questions developed for a large scale outcome generation study for a depression core outcome set which were developed with service users and healthcare professionals [25]. The question format was mirrored, but adapted for a mental health discharge theme. The views of a PPI (patient and public involvement), group sought to confirm appropriateness of questions and instructions (n=5).

After reading a participant information sheet and giving informed consent (by ticking a box), participants selected their stakeholder group (s) and watched a video that describes core outcome sets to non-experts. All participants were then presented with four open-ended questions relating to safe and effective discharge (see additional file 1). Participants were later presented with 3-5 questions specifically developed for their stakeholder group, additional file 1 outlines all of the questions. If a participant were a member of more than one

For peer review only - http://bmjopen.bmj.com/site/about/guidelines.xhtml

group, they answered questions relevant to multiple groups. Participants also answered a number of demographic questions: years of experience, country of residence, area of UK (if applicable), gender, age and email address for follow-up. The round was open for 6 weeks beginning December 10th 2018.

Qualitative data was coded to identify outcomes and thematically synthesised [26]. This involved line-by-line coding of text and development of descriptive themes, the final stage involved generating analytical themes, which were converted into potential outcomes where applicable. Outcomes were identified both indirectly, by extrapolating from service users' experiences (e.g. What would make discharge from an acute mental health ward safe in your opinion?), and directly, by asking specifically about outcomes (e.g. Can you think of any important outcomes to measure in research assessing discharge interventions?).

Outcomes from the systematic review [1] and qualitative surveys were combined to generate a long list of outcomes. This list, along with relevant quotes from the qualitative data, was discussed by the core research team in a structured meeting. Each outcome was considered in turn and each member had the opportunity to present arguments for or against inclusion. For each outcome, the group decided whether it should be a stand-alone outcome, combined with other codes of a similar thematic nature or removed from the process due to being of limited importance for a core outcome set. For example, we agreed to merge closely related items (e.g. *family relations* and *quality of interpersonal relationships*) and to exclude outcomes considered to be of limited importance (e.g., specific to a specialised area of care: *Autistic life*; or intervention *Antipsychotic Politherapy*). Unless there was a unanimous decision to merge or remove an outcome, it remained as a stand-alone outcome. The group decisions about each outcome are documented in additional file 2.

Stage 2: Delphi survey

The Delphi technique is a research method aimed at generating consensus. It solicits opinions from stakeholders groups in an iterative process of answering questions. After each round the responses are summarised and redistributed for discussion in the next round. We chose to have two rounds of Delphi in this study. The final outcome list that was decided upon after the group discussion in stage 1 was used to develop the first Delphi questionnaire. Any outcomes without consensus after the first round, were re-presented in round 2. The outcome list and instructions for the questionnaires were reviewed for face validity, understanding, and acceptability by a PPI group (n=5) and modified according to feedback.

A link to the survey was sent via email. Each round remained open for 14 days and participants received two follow-up reminder emails. Round 1 was open from late February 2019 to early March 2019, round 2 was late March 2019 to early April 2019. We ran the Delphi survey manually using Qualtrics: a secure online hosting platform [27]. In each round, participants were asked whether the items should become part of a core outcome set. A 7-point Likert scale was used, described as: Strongly Agree (7), Agree (6), Slightly Agree (5), Neither Agree nor disagree (4), Slightly Disagree (3), Disagree (2), Strongly Disagree (1). There is no definitive research indicating the optimal number of points to have on a Likert scale but scales between 5 and 9 points have been suggested as having the best reliability, so we chose a 7 point scale [28]. There was a free-text comments box and participants were encouraged to provide comments that would be fed-back anonymously to

For peer review only

the group. Participants could suggest additional outcomes at the end of round 1, which were reviewed by the core research team. Any outcome not already represented was added to round 2.

In round 2 median group scores for each outcome and anonymous comments for and against from the previous round were presented and participants were asked to reflect on the information presented and score each outcome again. The percentage of participant agreement with each outcome on a scale of 1–7 was calculated from the scores obtained during round 1 and again in round 2.

Literature suggests that consensus levels should be set a priori at a minimum of 70 percent [23,29]. We unanimously chose a 75% consensus level, slightly higher than the minimum to increase sensitivity, but to still allow for a varied pool of applicable outcomes given the tension in the literature around disagreement between service-user, health professional and policy-makers opinions of mental health outcomes [21]. Consensus criteria were defined a priori: outcomes scored as Agree or Strongly Agree (6-7) by 75% or more of the group reached consensus for inclusion and were included in the provisional core outcome set. Outcomes scored as Disagree or Strongly Disagree (1-2) by 75% or more were defined as having reached consensus for exclusion and were excluded. Outcomes not fulfilling criteria for consensus inclusion or exclusion were defined as not having reached consensus and were re-presented in round 2.

As no outcomes met the original criteria for having reached consensus for exclusion after round 1, it was agreed by the research team to redefine the criteria for having reached consensus for exclusion if 50% or less of participants scored the item as Strongly Agree or Agree (6-7). Reducing exclusion criteria after round 1 has been used effectively in past core outcome set research [30].

Stage 3: Consensus meeting

The results of the Delphi survey were presented at a consensus meeting. The main goal of the consensus meeting was to decide which items will be included in the final core outcome set. This was chaired by an independent researcher with expertise in consensus methodology, and who was not a member of the core research team. Participants were sampled to achieve a balanced representation of service users, health-care professionals, researchers and end-users of research. We aimed to have a small representative group of between 9 and 12 to enable meaningful small group discussions, similar to consensus meetings chaired by the facilitator in other fields [30,31]. International participation was restricted because of budgetary constraints.

The format of the consensus meeting comprised of a) a short overview of the study and b) a summary of the Delphi results sorted by stakeholder group, beginning with the outcomes that met consensus [32]. Outcomes identified in round 1 and 2 of the Delphi as having reached consensus for inclusion were presented first. Participants were asked if there were any fundamental reasons why these should not be included in the core outcome set. Divergent views were actively sought and the chair ensured everyone had opportunity to participate in discussions before voting commenced. Outcomes from the preliminary core outcome set were discussed in terms of feasibility and voted upon. Voting was conducted anonymously using cards in an envelope with bivariate response options (include/exclude). Voting and

For peer review only

consensus criteria followed the same format as in the Delphi (75% for inclusion). Results were presented after the voting of all outcomes had finished. Outcomes deemed to be having reached consensus for exclusion or with no consensus in the Delphi were reviewed and participants were asked if there were any fundamental reasons why these should be included in the core outcome set. Individual outcomes were discussed only if proposed as being important by a meeting participant. Outcomes meeting criteria for consensus were included in the core outcome set; all other items excluded. The meeting finished with the presentation and a final review and discussion of the core outcome set.

Patient and Public Involvement

Five patient representatives worked with researchers to develop the online questionnaires. Patients were represented alongside professionals and researchers in the consensus panel. One member of the research team (and co-author) is an expert by lived experience and was involved in all design and analysis decisions.

Ethics and registration

Our findings are reported in line with the Core Outcome Set-Standards for Reporting (COS-STAR) guidance [32]. The study was prospectively registered with the COMET initiative (1276). The study was approved by the University of Nottingham Business School ethics committee and all participants gave informed consent

Results

Stage 1: Information gathering

Our systematic review has been described in detail elsewhere [1]. In summary 69 outcome categories were identified from 45 studies. Ninety-three participants in total, from 12 countries completed the information gathering questionnaire. However, as aforementioned, many identified with more than one stakeholder group, therefore we do not have absolute homogenous stakeholder group numbers, 27 identified as service users, 17 family/carers, 39 health-care professionals, 15 end-user of research and 37 researchers. Additional file 3 presents participants demographics. Qualitative questionnaires revealed an additional 45 outcomes that were not identified in the literature (for example, outcomes concerning involvement in discharge planning, see additional file 2). After discussion within the research team, 82 standardised outcome terms were taken forward into the Delphi process; 19 outcomes were combined/collapsed and 13 were removed, see additional file 2.

Stage 2: Delphi process

Sixty-nine participants completed round 1 of the Delphi (22 service users, families and carers, 26 researchers and 21 healthcare professionals and decision makers) and 68 participants completed round 2 (30 researchers, 18 service users and families and 20 healthcare professionals and decision makers). Whilst 5 participants dropped out after round 1, 4 participants joined the panel in round 2 (these individuals participated the qualitative questionnaire but not round 1). There was a 1.4% attrition between round 1 and 2 of the Delphi. Seven additional outcomes were proposed by participants during round 1, of which

two were added into round 2 after a core team discussion. The full list of Delphi items is available in additional file 4.

After round 1, 14 outcomes met the criteria for consensus inclusion (75% or more agreed/strongly agree with that outcome, see table 1). Twenty outcomes met the revised

	Percentage agreement	Percentage Disagreement	Median	Researchers	Service users and familiess	HCPs and DMs
Service user involvement in discharge planning (inc. feeling listened to)	87%	4%	7	65%	100%	95%
Functioning (health, social, etc.)	83%	3%	6	69%	100%	81%
Mental health and illness (symptom/psychological distress)	83%	3%	6	73%	91%	86%
Personal Recovery	82%	1%	6	75%	86%	86%
Service user understanding of discharge plan	81%	3%	6	65%	91%	86%
Quality of life	81%	1%	6.5	65%	90%	86%
Suicide Completed	80%	4%	7	80%	90%	68%
Readmission	80%	6%	6	77%	77%	86%
Service user involvement in decision making (shared decision making)	77%	4%	7	50%	95%	86%
Service user satisfaction with information provision at discharge (e.g. regarding medication, risk, crisis planning)	77%	6%	6	65%	86%	81%

criteria for having reached consensus for exclusion (50% or less of participants agreed/strongly agreed with that outcome). Forty-eight outcomes did not meet consensus criteria for inclusion or exclusion and were re-presented to the group in round 2. Therefore, 50 outcomes were presented in round 2, only one outcome met the criteria for consensus after this round: meaningful activity. No outcomes met criteria for exclusion and 49 did not meet consensus. Additional file 4 shows consensus levels for each outcome in each round.

Table 1: The Preliminary Core Outcome Set at the end of the online Delphi.

For peer review only

Service user knowledge of how to access community support (i.e. in an emergency)	77%	3%	6	58%	91%	86%
Recurrence (i.e. relapse)	75%	1%	6	58%	91%	76%
Suicide Attempted	75%	4%	6	62%	86%	81%
Discharge to appropriate accommodation	75%	3%	6	69%	91%	67%
Meaningful Activity (included in Round 2)				73%	80%	79%

For peer review only

Stage 3: Consensus Meeting

Eleven participants attended the consensus meeting, as in previous rounds these categories were not exclusive, six participants were researchers, three identified as service users, three as healthcare professionals and three end-users of research, see table 2. Table 3 shows the quantitative results of the meeting.

The preliminary 15-item core outcome set was considered individually and discussions indicated that many of the outcomes were elements of an ideal discharge, and process outcomes/variables, but probably not measurable outcomes that should be included in a core outcome set. After these discussions and independent and anonymous voting, five items no longer met consensus criteria for inclusion. First, 'service user involvement' in discharge planning, and the associated items 'Service user understanding of discharge plan', 'Service user involvement in decision making', 'Service user satisfaction with information provision at discharge'; and 'Service user knowledge of how to access community support'. There was a discussion that these are very important elements of a successful discharge, but not core outcomes due to issues surrounding validity and meaning.

'Mental health and illness' was initially close to consensus with 73% consensus to include, however those that chose to exclude found it to be too vague, and articulated that they were most interested in measuring acute psychological distress, rather than mental health and illness. The service user representatives in the group interpreted "recovery" to mean a complete amelioration of symptoms and even when in "recovery" individuals described continuing to experience distress and difficulties with their mental health. We chose to therefore separate the broader mental health and illness outcome into self-reported psychological distress and clinician reported mental health. The granular outcome of self-reported psychological distress resulted in 100% consensus to include. On the contrary clinician reported mental health did not meet consensus criteria (45%). Similar discussions happened around the recurrence (relapse) outcome, whereby its inclusion in a core outcome set, would ultimately necessitate buy-in to criteria model, which suggested that mental health problems could and should be completely resolved.

Discussions around the 'suicide attempted' outcome indicated that participants felt that suicide attempts or self-harm had diverse motivations and definitions and they discussed the issues of delineating the boundaries of self-harm and suicide attempts and how this is documented. After the consensus meeting this outcome no-longer meet consensus criteria to include. Discussions surrounding personal recovery, functioning and meaningful activity indicated that participants considered these outcomes too vague and subjective to be a component of a core outcome set. There was consensus to exclude meaningful activity and recovery, and no consensus to include personal recovery. There was consensus to exclude discharge to appropriate accommodation, discussion indicated this was primarily because this spanned the health and social care boundaries and may not be applicable to every intervention.

On completion of the meeting, only four outcomes met consensus criteria for inclusion, see Table 4. A core outcome set of four was agreed, participants agreed that the following should be included: readmission, quality of life, suicide completed and service user reported psychological distress. Readmission was the most frequently used outcome in past research, and despite limitations, participants felt it was one of the only proxy measures of appropriate discharge. Quality of life and psychological distress were considered important ways of quantitatively assessing the psycho-social elements of discharge; which are of primary importance. Suicide completed was considered rare but imperative data to capture given the research highlighting the relationship between acute mental health discharge and suicide highlighted by a growing body of literature [5,33]. Figure 1 shows the process undertaken to reach the core outcome set.

Table 2: Participants that attended consensus meeting

PP Number	Researcher	Service User	Healthcare Professional	End-user of Research
1	X			
2	X			
3	X			
4	X		X	
5	X	X		
6	X			X
7			X	
8			X	X
9		X		
10		X		
11				X
Total	6	3	3	3

Table 3: Outcomes of consensus meeting, levels of consensus in anonymous voting

	Include	Exclude	Percentage
Readmission	10	1	91%

For peer review only

Service user reported psychological distress	11	0	100%
Suicide completed	9	2	82%
QoL	9	2	82%
Reoccurrence	4	7	36%
Mental Health and Illness	8	3	73%
Service user involvement in decision making	7	4	64%
Personal Recovery	6	5	55%
Meaningful Activity	1	10	9%
Functioning	1	10	9%
Clinician Reported Mental Health	5	6	45%
Service User Satisfaction with information provision at discharge	3	8	27%
Service user understanding of the discharge plan	3	8	27%
Suicide Attempted	3	8	27%
Service User Involvement in Discharge Planning	6	5	55%
Knowledge of how to access support in a crisis	5	6	45%
Discharge to appropriate accommodation	0	11	0%

For peer review only

Table 4: The final core outcome set

Final Core Outcome Set	
1	Readmission
2	Quality of Life
3	Suicide Completed
4	Service User Reported Psychological Distress

<insert figure 1>

Discussion

This study provides the first international consensus on outcomes for intervention studies concerning discharge from an acute adult mental health inpatient setting. We could not identify any other published core outcome sets for interventions concerning discharge from acute mental health services. Moreover, there are very few core outcome sets for mental health, despite recommendations for consensus in the literature [18,21]. All the included outcomes were agreed upon by more than 75% of a group of relatively equally-represented service-users and family/carers, health-care professionals, researchers and end-user of research using consensus methods. We recommend that all future research studies evaluating interventions for discharge from acute adult mental health settings use this core outcome set as a framework for outcome selection, to compliment, rather than replace any other outcomes that are relevant to their research question. As discharge from acute services is a particularly challenging period for those experiencing mental health problems [3,33], it's important to understand what interventions work and more specifically which elements of an intervention improve which particular outcomes. This core outcome set provides a framework for between-study comparison, ultimately enabling researchers to articulate the theory of change that underpins interventions.

In our systematic review [1], we identified 22 studies that reported readmission rates as an outcome, yet almost all of them captured this in different ways: some used self-report data, some clinical case notes or some retrospective administrative data, others used case manager's reports. In addition the time markers were variable, some used country specific time markers in line with policy such as 28 days in the UK [34], whilst others chose a series of time markers such as within 1 month, 3 month and 6 months, but the time markers were rarely directly comparable. Similarly, six studies measured quality of life but, only two used the same measurement instrument (Lehman's Quality of Life) [16,35]. In the current study, we have developed consensus that Quality of Life and Readmission are important and feasible to measure, robust recommendations of how best to measure these are now needed.

There were some unexpected exclusions in the core outcome set, for example mental health symptoms and treatment adherence were frequently used in past research [1], but not included in the core outcome set. In the background of this paper we described the recent Kings Fund report that suggested generating agreement amongst healthcare professionals, service users, policy makers and researchers is a difficult but imperative task [21]. Our work reiterates this findings, and the small four-item core outcome set represents the only outcomes that are unanimously agreed upon, despite so many outcomes being of upmost importance to service-users and families.
This research has further highlighted the importance of shared decision making and service-user and family involvement to all stakeholder groups [36].

This study indicates an impending desire to assess service-user satisfaction andinvolvement in the process. Whilst such outcomes, were excluded in later stages of this research, it does not reduce their prospective importance in discharge interventions or provision of care at discharge. The five most agreed upon elements of service-user involvement and satisfaction in discharge were: Service user involvement in discharge planning; Service user understanding of discharge plan; Service user involvement in decision making; Service user satisfaction with information provision at discharge; Service user knowledge of how to access community support. Policy makers and healthcare management might consider measuring these five things in local level initiatives as overriding principles of care to ensure they are not missing from care provision.

For peer review only

Research highlighting the importance of involving service users in mental health care planning is emerging, along with measures of such activity. Therefore, we suggest that future research could include a service user reported outcome measure of involvement alongside the 4-item core outcome set and any other chosen measures. This could be measured in an existing instrument of service-user involvement care planning in mental health, such as the EQUIP PROM (Patient Reported Outcome Measure) [36]. The 6 outcomes described above can also be presented as self-reported Likert measure of service user involvement in discharge planning (see additional file 5). These 6 items are developed from synthesis of academic literature, qualitative questionnaires and met criteria for consensus amongst experts in round 2, so from a psychometric perspective would arguably meet initial face and content validity criteria [37].

The difficulties of developing a mental health core outcome set was further epitomised when applied to care transitions: a service-level (rather than specific clinical population) multi-agency, multi-stage, complex period of the care pathway [3,22]. Generating a set of meaningful applicable outcomes that span primary and secondary care, across multiple physical locations, that are relevant for every service user was imperative. For example, a great deal of past literature focuses on housing interventions [38–40], and whilst housing is a significant safety issue at discharge, it's not necessarily relevant to all service users. This multi-agency, multi-morbidity complexity was arguably one factor that resulted in the small set of generic outcomes, that arguably differs from narrowly defined clinical core outcome set reported in past literature of many more outcomes [41,42].

This study had several strengths. Our method is based on recommendations from an international panel of experts [23]. Inclusion of service-users and health-care professionals at every stage ensured that outcomes in the final core set embody shared priorities. The comprehensive and laborious long-list process ensured all potential outcomes were considered in the course of the consensus process. However, there were some limitations to our study. The research was only conducted in English, due to budgetary constraints, although our online rounds included participants from 12 countries. Furthermore, in many consensus meetings additional outcomes are often added, the method infrequently serves as means of reducing the number of outcomes included in the preliminary core outcome set from the Delphi [30]. However, in our case we found that the group did not agree with many of the outcomes and it was reduced to a very small COS of 4 items. This is beneficial in some ways, as we hope it is easier for researchers to operationalise a four-item core outcome set.

The use of outcomes in mental health research and service, is becoming more contested in terms of what is meaningful and effective, it could be argued that core outcomes sets are less applicable to mental health populations than general health populations, given the complexity of mental health problems and the subjectivity of measuring it. However, as core outcomes sets are relatively uncommon in mental health, we believe (similar to other clinical populations) a small, agreed, feasible set of core outcomes will facilitate between study comparability and advancement in evidence collection [19,23].

Future Directions

Development of this core outcome set involved participation of stakeholders from 12 different countries; (primarily researchers) however, we recommend that further work should be undertaken to validate this core outcome set more widely, particularly in non-English

For peer review only

speaking populations. The two of the final four outcomes and many of the preliminary 15 outcomes to emerge from the Delphi, are not necessarily specific to mental health care transitions. Some outcomes are comparable to a similar core outcome set for care transitions of adolescents and young adults with special healthcare needs [43]. Future research may consider a 'transitions of care' core outcome set, to reduce the number of similar core outcome sets.

Another key priority to make this core outcome set operationalised is to agree upon measurement criteria using the COSMIN guidelines [44]. We conducted some preliminary questionnaires with the Delphi panel to produce preliminary measurement recommendations, however there was very little agreement amongst panellists (see additional file 6). Another key priority to make this core outcome set operationalised is to agree upon measurement criteria using the COSMIN guidelines [44]. We conducted some preliminary questionnaires with the Delphi panel to produce preliminary measurement recommendations, however there was very little agreement amongst panellists (see additional file 6). The recommended measures by the panel were Kessler Psychological Distress (K10) and Recovery Quality of Life (ReQoL) within one month of discharge [45,46]. Readmission and suicide completed rates were recommended to be captured within 28 days of discharge using retrospective review of administrative data. However, these are only preliminary recommendations and we highly recommend a future study following COSMIN guidelines.

Conclusion

The four outcomes included in our outcome set represent the consensus opinion of a group of service-users, health-care professionals, and international researchers and addresses an unmet necessity: assisting researchers in the design, implementation and reporting of interventions that aim to improve discharge from acute mental health settings. Ultimately, application of this core outcome set will enhance the relevance of future interventions to health-care professionals, the research community and service-users. If used, the core outcome set could provide more evidenced-based interventions, underpinned by theory of change outlining the relationships between the component of the intervention and the outcome it should improve[1,47]; which should increase service-user safety at this distressing time period.

Patients' consent and permission to publish

All participants gave informed consent to be involved in the study ahead of data collection.

Author Contributions

NT conceived the design of the study; conducted the literature search, meta-synthesis and Delphi; analysed the data; and drafted the majority of the manuscript. JW and NW contributed equally and provided oversight of the study design, analysed and synthesised the data and contributed significantly to the drafting of the manuscript. AG analysed and synthesised data, provided an expert by lived experience opinion on decisions made in regards to wording, study design, PPI involvement, and also contributed adaptations to the manuscript.

Conflicts of Interest

For peer review only

The authors report no conflicts of interest

Ethics Committee Approval

This research received a favourable ethics opinion from Nottingham University Business School Ethics Committee.

Role of Funding Source

This research is funded by the NIHR Greater Manchester Patient Safety Translational Research Centre, they had no role in the writing of manuscript or decision to submit.

Data Statement

Data is available upon reasonable request

Acknowledgements

We would like to thank the patient and public involvement group for their help in designing the questionnaires. The following participants chose to be acknowledged for their participation and the remainder chose to remain anonymous:

Amy Clowes, Andrew Gallivan, Anna Hegedus, Anna Willis, Bernd Puschner, Cara Sturgess, Cassandra Lovelock, Cheryl Forchuk RN PhD, Colette Ramsey, David Cochrane, David Smelson, David Smith, Deb Smith, Dr Leah Quinlivan, Dr Nicola Clibbens, Dr Sarah Markham, Dr. Pucciarelli Gianluca, PhD, E Thomas, Emma Ward, Hameed Khan, Hazel Nash, Helene Provencher, Jan Hutchinson, Jane McNeice, Jean Nicholls, Justin Scanlan, Kyri Gregoriou, Law, Yik Wa, Liz Monaghan, Mark S. Smith, Mark Trewin, Matthew Mckenzie, Michael P. Hengartner, Mohammad Ghadirivasfi, Mrs Z Burns, Philippe Golay, Phyllis Solomon, Ph.D., Prof Roger Webb, Rebecca Stack, Sarah, Sarah Gunn, Sarah Markham, Shaun Thomson, Umar Kankiya, Usha Sambamoorthi, Zoe Swithenbank

Figure 1 Caption: Process of reducing the outcomes to a set of four core outcomes

Word Count: 5250

References

[1] Tyler N, Wright N, Waring J. Interventions to improve discharge from acute adult mental health inpatient care to the community: systematic review and narrative synthesis. BMC Health Serv Res 2019;19:883. https://doi.org/10.1186/s12913-019-4658-0.

[2] Waring J, McDonald R, Harrison S. Safety and complexity: Inter-departmental relationships as a threat to patient safety in the operating department. J Heal Organ Manag 2006. https://doi.org/10.1108/14777260610662753.

[3] Wright N, Rowley E, Chopra A, Gregoriou K, Waring J. From admission to discharge in mental health services: a qualitative analysis of service user involvement. Heal Expect 2016;19:367–76. https://doi.org/10.1111/hex.12361.

For peer review only

[4] Walter F, Carr MJ, Mok PLH, Astrup A, Antonsen S, Pedersen CB, et al. Premature Mortality Among Patients Recently Discharged From Their First Inpatient Psychiatric Treatment. JAMA Psychiatry 2017;74:485–92. https://doi.org/10.1001/jamapsychiatry.2017.0071.
[5] Burns J, Flynn S, Lowe R, Turnbull P, Baird A, Stones P, et al. The National Confidential Inquiry into Suicide and Homicide by People with Mental Illness. 2017.
[6] Abraham O, Myers MN, Brothers AL, Montgomery J, Norman BA, Fabian T. Assessing need for pharmacist involvement to improve care coordination for patients on LAI antipsychotics transitioning from hospital to home: A work system approach. Res Social Adm Pharm 2017;13:1004–13. https://doi.org/https://dx.doi.org/10.1016/j.sapharm.2017.02.006.
[7] Exbrayat S, Coudrot C, Gourdon X, Gay A, Sevos J, Pellet J, et al. Effect of telephone follow-up on repeated suicide attempt in patients discharged from an emergency psychiatry department: a controlled study. BMC Psychiatry 2017;17. https://doi.org/10.1186/s12888-017-1258-6.
[8] Vigod SN, Kurdyak PA, Dennis C-L, Leszcz T, Taylor VH, Blumberger DM, et al. Transitional interventions to reduce early psychiatric readmissions in adults: systematic review. Br J Psychiatry 2013;202:187–94. https://doi.org/10.1192/bjp.bp.112.115030.
[9] Waring J, Bishop S, Marshall F, Tyler N, Vickers R. An ethnographic study comparing approaches to inter-professional knowledge sharing and learning in discharge planning and care transitions. J Heal Organ Manag 2019.
[10] Walker SA, Naji SA, Howie FL, Andrew J, Cameron IM, Eagles JM. The effectiveness of psychiatric discharge coordinators. Prim Care Psychiatry 2000. https://doi.org/10.1185/135525700543370.
[11] Bennewith O, Evans J, Donovan J, Paramasivan S, Owen-Smith A, Hollingworth W, et al. A Contact-Based Intervention for People Recently Discharged from Inpatient Psychiatric Care: A Pilot Study. Arch Suicide Res 2014. https://doi.org/10.1080/13811118.2013.838196.
[12] D'souza R. Improving Treatment Adherence and Longitudinal Outcomes in Patients with a Serious Mental Illness by Using Telemedicine. J Telemed Telecare 2002. https://doi.org/10.1177/1357633X020080S251.
[13] Exbrayat S, Coudrot C, Gourdon X, Gay A, Sevos J, Pellet J, et al. Effect of telephone follow-up on repeated suicide attempt in patients discharged from an emergency psychiatry department: A controlled study. BMC Psychiatry 2017. https://doi.org/10.1186/s12888-017-1258-6.
[14] Forchuk C, Martin ML, Chan YL, Jensen E. Therapeutic relationships: From psychiatric hospital to community. J Psychiatr Ment Health Nurs 2005;12:556–64. https://doi.org/10.1111/j.1365-2850.2005.00873.x.
[15] Juven-Wetzler A, Bar-Ziv D, Cwikel-Hamzany S, Abudy A, Peri N, Zohar J. A pilot study of the " Continuation of Care" model in " revolving-door" patients. Eur Psychiatry 2012;27:229–33. https://doi.org/10.1016/j.eurpsy.2011.05.003.
[16] Jensen E, Chapman P, Davis A, Forchuk C, Seymour B, Witcher P, et al. An Evaluation of Community-Based Discharge Planning in Acute Mental Health Care. Can J Community Ment Heal 2010;29:111–24. https://doi.org/10.7870/cjcmh-2010-0038.
[17] Steffen S, Kösters M, Becker T, Puschner B. Discharge planning in mental health care: A systematic review of the recent literature. Acta Psychiatr Scand 2009;120:1–9. https://doi.org/10.1111/j.1600-0447.2009.01373.x.
[18] Steffen S, Kösters M, Becker T, Puschner B. Discharge planning in mental health care:

For peer review only

A systematic review of the recent literature. Acta Psychiatr Scand 2009;120:1–9. https://doi.org/http://dx.doi.org/10.1111/j.1600-0447.2009.01373.x.

[19] Williamson PR, Altman DG, Bagley H, Barnes KL, Blazeby JM, Brookes ST, et al. The COMET Handbook: Version 1.0. Trials 2017. https://doi.org/10.1186/s13063-017-1978-4.

[20] Williamson PR, Altman DG, Blazeby JM, Clarke M, Devane D, Gargon E, et al. Developing core outcome sets for clinical trials: Issues to consider. Trials 2012. https://doi.org/10.1186/1745-6215-13-132.

[21] Lang K. Outcomes for mental health services: what really matters? | HSR UK. 2019.

[22] Waring J, Marshall F, Bishop S, Sahota O, Walker M, Currie G, et al. An ethnographic study of knowledge sharing across the boundaries between care processes, services and organisations: the contributions to 'safe' hospital discharge. Heal Serv Deliv Res 2014. https://doi.org/10.3310/hsdr02290.

[23] Kirkham JJ, Davis K, Altman DG, Blazeby JM, Clarke M, Tunis S, et al. Core Outcome Set-STAndards for Development: The COS-STAD recommendations. PLoS Med 2017. https://doi.org/10.1371/journal.pmed.1002447.

[24] Pedersen ER, Helmuth ED, Marshall GN, Schell TL, PunKay M, Kurz J. Using Facebook to Recruit Young Adult Veterans: Online Mental Health Research. JMIR Res Protoc 2015. https://doi.org/10.2196/resprot.3996.

[25] Contributed Talks. J Evid Based Med 2019;12:24–7. https://doi.org/10.1111/jebm.12336.

[26] Thomas J, Harden A. Methods for the thematic synthesis of qualitative research in systematic reviews. BMC Med Res Methodol 2008;8:45. https://doi.org/10.1186/1471-2288-8-45.

[27] Qualtrics. Qualtrics: The World's Leading Research & Insights Platform. Qualtrics 2016. https://doi.org/10.3109/03639045.2013.790903.

[28] Preston CC, Colman AM. Optimal number of response categories in rating scales: Reliability, validity, discriminating power, and respondent preferences. Acta Psychol (Amst) 2000;104:1–15. https://doi.org/10.1016/S0001-6918(99)00050-5.

[29] Hsu C, Sandford B. The delphi technique: making sense of consensus. Pract Assessment, Res Eval 2007;12:1–8. https://doi.org/10.1016/S0169-2070(99)00018-7.

[30] Bell BG, Spencer R, Avery AJ, Campbell SM. Tools for measuring patient safety in primary care settings using the RAND/UCLA appropriateness method. BMC Fam Pract 2014;15:110. https://doi.org/10.1186/1471-2296-15-110.

[31] Avery AJ, Dex GM, Mulvaney C, Serumaga B, Spencer R, Lester HE, et al. Development of prescribing-safety indicators for GPs using the RAND Appropriateness Method. Br J Gen Pract 2011;61:e526–36. https://doi.org/10.3399/bjgp11X588501.

[32] Kirkham JJ, Gorst S, Altman DG, Blazeby J, Clarke M, Devane D, et al. COS-STAR: A reporting guideline for studies developing core outcome sets (protocol). Trials 2015. https://doi.org/10.1186/s13063-015-0913-9.

[33] Walter F, Carr MJ, Mok PLH, Antonsen S, Pedersen CB, Shaw J, et al. Suicide Methods and Specific Types of Accidental Death and Fatal Poisoning Among Discharged Psychiatric Patients: A National Cohort Study. J Clin Psychiatry 2018;79. https://doi.org/10.4088/JCP.17m11809.

[34] Ali AM, Bottle A. How do we interpret readmission rates? BMJ 2018. https://doi.org/10.1136/bmj.k3104.

[35] Tomita A, Lukens EP, Herman DB. Mediation analysis of critical time intervention for persons living with serious mental illnesses: assessing the role of family relations in reducing psychiatric rehospitalization. Psychiatr Rehabil J 2014;37:4–10.

For peer review only

https://doi.org/10.1037/prj0000015.
[36] Lovell K, Bee P, Brooks H, Cahoon P, Callaghan P, Carter L-A, et al. Embedding shared decision-making in the care of patients with severe and enduring mental health problems: The EQUIP pragmatic cluster randomised trial. PLoS One 2018;13:e0201533. https://doi.org/10.1371/journal.pone.0201533.
[37] Falzarano M, health GZ-J of allied, 2013 undefined. Seeking consensus through the use of the Delphi technique in health sciences research. IngentaconnectCom n.d.
[38] Forchuk C, Russell G, Kingston-Macclure S, Turner K, Dill S. From psychiatric ward to the streets and shelters. J Psychiatr Ment Health Nurs 2006;13:301–8. https://doi.org/http://dx.doi.org/10.1111/j.1365-2850.2006.00954.x.
[39] Forchuk C, Godin M, Hoch JS, Kingston-MacClure S, Jeng M (Mo) S, Puddy L, et al. Preventing Homelessness After Discharge from Psychiatric Wards: Perspectives of Consumers and Staff. J Psychosoc Nurs Ment Health Serv 2013;51:24–31. https://doi.org/10.3928/02793695-20130130-02.
[40] Tomita A, Herman DB. The Role of a Critical Time Intervention on the Experience of Continuity of Care Among Persons With Severe Mental Illness After Hospital Discharge. J Nerv Ment Dis 2015;203:65–70. https://doi.org/10.1097/NMD.0000000000000224.
[41] Fish R, Sanders C, Adams R, Brewer J, Brookes ST, DeNardo J, et al. A core outcome set for clinical trials of chemoradiotherapy interventions for anal cancer (CORMAC): a patient and health-care professional consensus. Lancet Gastroenterol Hepatol 2018. https://doi.org/10.1016/S2468-1253(18)30264-4.
[42] Boers M, Brooks P, Simon LS, Strand V, Tugwell P. OMERACT: An international initiative to improve outcome measurement in rheumatology. Clin Exp Rheumatol 2005;23. https://doi.org/10.1186/1745-6215-8-38.
[43] Fair C, Cuttance J, Sharma N, Maslow G, Wiener L, Betz C, et al. International and Interdisciplinary Identification of Health Care Transition Outcomes. JAMA Pediatr 2016;170:205. https://doi.org/10.1001/jamapediatrics.2015.3168.
[44] Mokkink LB, Terwee CB, Knol DL, Stratford PW, Alonso J, Patrick DL, et al. The COSMIN checklist for evaluating the methodological quality of studies on measurement properties: A clarification of its content. BMC Med Res Methodol 2010;10. https://doi.org/10.1186/1471-2288-10-22.
[45] Keetharuth AD, Brazier J, Connell J, Bjorner JB, Carlton J, Buck ET, et al. Recovering Quality of Life (ReQoL): A new generic self-reported outcome measure for use with people experiencing mental health difficulties. Br J Psychiatry 2018. https://doi.org/10.1192/bjp.2017.10.
[46] Andrews G, Slade T. Interpreting scores on the Kessler Psychological Distress Scale (K10). Aust N Z J Public Health 2001. https://doi.org/10.1111/j.1467-842X.2001.tb00310.x.
[47] Davidoff F, Dixon-Woods M, Leviton L, Michie S. Demystifying theory and its use in improvement. BMJ Qual Saf 2015;24:228–38. https://doi.org/10.1136/bmjqs-2014-003627.

For peer review only

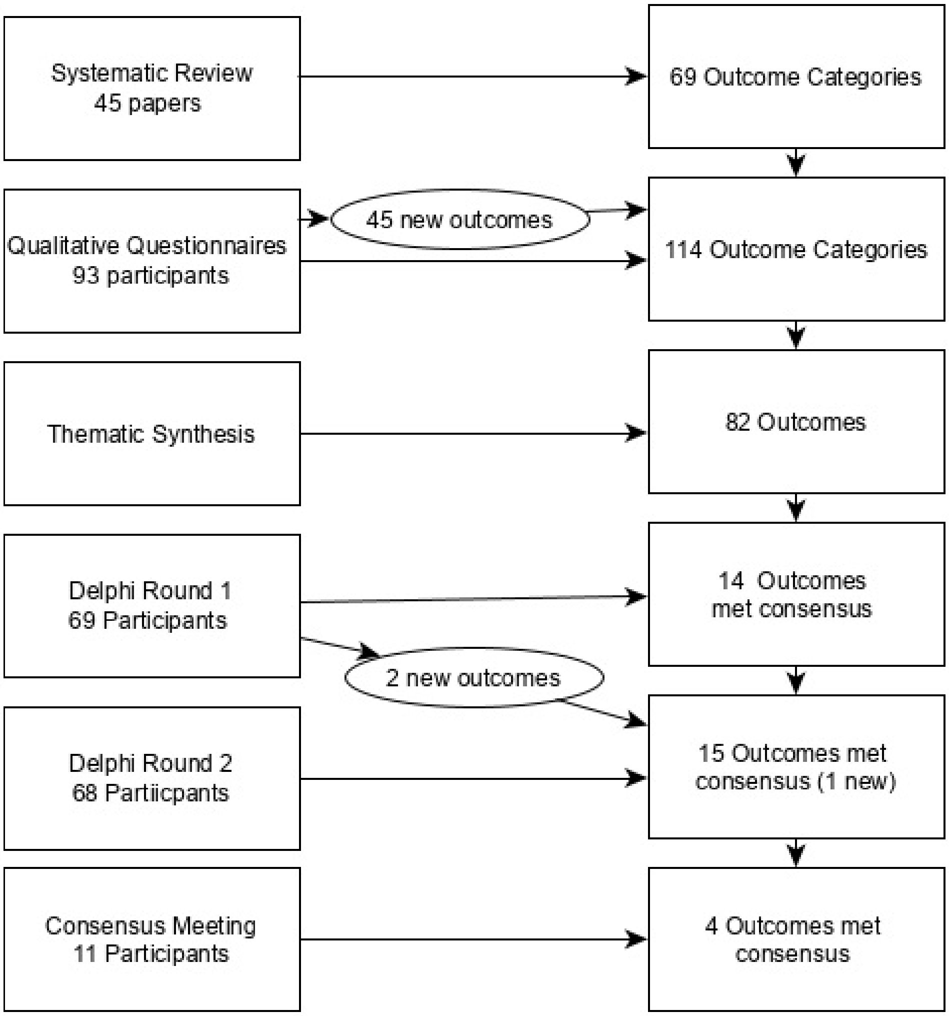

Process of reducing the outcomes to a set of four core outcomes

133x142mm (96 x 96 DPI)

Supplementary Material

File 1: Table of questions asked in qualitative questionnaire

Group	Question
All Participants	What would make discharge from an acute mental health ward **safe** in your opinion?
All Participants	What would make discharge from an acute mental health ward **effective** in your opinion?
All Participants	What would you like to see prioritised for research into discharge from an acute ward?
All Participants	Can you think of any important outcomes to measure in research assessing discharge interventions?
Service User	What do you think is the most difficult aspect of discharge from a mental health acute ward?
Service User	What might improve discharge from a mental health ward?
Service User	Was there any one person/group/intervention which really made a difference for you? If so what was it? What made it so powerful?
Service User	Do you have any other feelings or concerns not addressed in previous questions?
Family member/carer	What do you consider to be the most difficult components of discharge for your family member/friend from an acute mental health ward?
Family member/carer	What types of improvement would you expect to see in your family member/friend following a successful discharge from an acute mental health ward?
Mental health professional	What do you consider to be the most difficult aspects of discharge from an acute unit for your service-users?
Mental health professional	Are there any changes/behaviours you would expect to see in a service-user during or following a safe and/or effective discharge?
Researchers	If applicable, what outcomes did you measure in past research (of discharge interventions)?
Researchers	Are there any concepts that you think are important to measure, but chose not to, due to not having a suitable measurement instrument?
End users of research	If you were looking to use research to inform changes to the discharge procedure within your professional role, what outcomes would you like to see reported?
End users of research	In your opinion, what measurements would persuade you that a discharge intervention is effective?

For peer review only

File 2: Results of Meta-synthesis of Outcomes and data from questionnaire

Clinical

From Review	Additional from Meta-synthesis
Reoccurrence (i.e. Relapse)	Stability
Clinical Recovery	Physical Health and Wellbeing
Personal Recovery	Iatrogenic harm (patient safety incidents- harm caused by the care system)
Substance Misuse (inc. alcohol)	Serious accidental injury
Self-injury (i.e. harm to self)	Mortality
Suicide Risk	Mental health and illness (symptoms/psychological distress)
Suicidal Ideation	
Suicide Attempted	
Suicide Completed	
Medication Knowledge	
Medication Adherence	
Medication Side Effects	
Medication Management	
Global Functioning	

Service

From Review	Additional from Meta-synthesis
Emergency department visits post discharge	Police intervention post discharge
Readmission	Engagement with psychological intervention
Length of stay pre-discharge	Engagement with community services
Length of stay post-discharge (i.e. subsequent readmissions)	Availability of appropriate community support (i.e. allocated key worker)
Outpatient appointment/visit adherence	Length of time before follow up/aftercare
Service use	Leaving the hospital against medical advice
	Service user involvement in decision making (shared decision making)
	Serious incidents (i.e. serious incidents reported formally)

Satisfaction

From Review	Additional from Meta-synthesis
Service user expectations of care	Professionals/care teams satisfaction with information provision at discharge
Service user satisfaction with discharge	Service user satisfaction with information provision at discharge (e.g. regarding medication, risk, crisis planning)
Service user experience of discharge	
Service user satisfaction with treatment	
Carer/family/other satisfaction with discharge	
Staff satisfaction with discharge	

Personal

From Review	Additional from Meta-synthesis
Hopelessness	Feelings of safety
Boredom	Feelings of support (from various groups- i.e. family, professionals and friends)
Loneliness	Experience of stigma
Isolation	Quality of interpersonal relationships (friends and family)
Coping skills	Self-management
Concern/anxiety about discharge	
Autonomy (e.g. independence, autonomous decision making)	

For peer review only

Housing Stability	
Discharge to appropriate accommodation	
Service user knowledge of own condition	
Quality of life	
Community participation	
Violence	
Aggression	
Victim of crime	
Stable relationships with health professionals	
Employment	

Discharge Planning

From Review	Additional from Meta-synthesis
	Service user involvement in discharge planning
	Family/carer involvement in discharge planning
	Advocate involvement in discharge planning
	Completion of planned care
	Continuity of contact
	Service user readiness for discharge (incl. preparation)
	Clinical readiness for discharge
	Experience of coercion at discharge (e.g. feeling forced to leave)
	Provision of financial support (e.g. benefits advice, debt advice)
	Delays in expected discharge
	Service availability (e.g. availability of community services, CPN, follow-up, social worker, bed in residential service)
	Family/carer/other readiness for discharge
	Staff understanding of safe and effective discharge
	Plan for community engagement
	Is there a plan for care post discharge created
	Has the plan for care post-discharge been communicated with patient
	Has information about the discharge been reported to other relevant services

Changes/Combinations

Outcomes from Review	Changes/Combinations
Social Recovery	Combined into quality of interpersonal relationships
Relapse	PPI suggestion change to reoccurrence
Self-harm	PPI suggestion change to self-injury
Depression	Combined into symptoms
Anxiety	Combined into symptoms
Illegal drug use	Combined into substance misuse
Alochol use	Combined into substance misuse
Addiction severity	Combined into substance misuse
Crisis planning	Combined into information provision
Risk communication	Combined into information provision
Allocated worker	Combined into availability of appropriate community support
Better knowledge transfer	Combined into information provision (professional)
Therapeutic alliance	Combined into stable relationships with HPs
Contact with ambulatory care	Combined into emergency visits
Treatment adherence	Combined into medication adherence and engagement with psychological services
7 day follow up	Changed to length of time before follow up for global audience
Psychological distress	Combined into symptoms
Family relations	Combined into quality of interpersonal relationships
Community integration and functioning	Combined into Community participation

Outcomes Removed from Process

Outcomes from Review	Reason
Antipsychotic Politherapy	Too specific
Autistic life	Too specific

Medication Problems	Too vague
Functional Recovery	Meaning not explicit
Satisfaction with service in community	Not relevant for this COS
Global satisfaction with discharge	Meaning not explicit
Quality of care	Too vague
Treatment decision making	Not relevant for this COS
Financial cost to provider	Impossible to measure
Number of beds	Too vague
Staff workload	Too vague
Freedom	Too vague
Self-activity	Too vague

Carer outcomes

**Outcomes from Review**	**Decision**
Caregiver Burden	Yes
Caregiver Health Status	No (too specific)
Caregiver Knowledge about illness	Yes
**From Synthesis**	
Carer support	No
Relationship between family and SU	No (probably a variable)

For peer review only

File 3: Participant Demographics

**Categories**											
**Group**	**Service Users**	**Families and Carers**	**Healthcare Professionals**	**Researchers**	**End-users of Research**						
**n**	27	17	39	37	15						
**Gender**	**Male**	**Female**	**Other**								
**n**	28	63	0								
**Age**	**18-24**	**25-34**	**35-44**	**45-54**	**55-64**	**65-74**	**75-84**				
**n**	4	20	30	24	10	4	1				
**Location**	**International**	**East of England**	**East Midlands**	**London**	**North West**	**North East**	**South East**	**South West**	**Wales**	**West Midlands**	**Yorkshire and the Humber**
**n**	24	2	11	7	16	1	6	5	0	13	7

Locations for International participants

**Country**	**N**
Australia	4
Canada	2
China	1
France	1
Germany	1
Iran	1
Italy	1
Northern Ireland	1
South Africa	1
Switzerland	4
USA	7

For peer review only

File 4: Full list of Delphi outcomes, consensus levels, and round of inclusion/exclusion

	R1 Percentage agreement	R1 Percentage Disagreement	Median	Researchers	Sus and carers	HCPs and DMs	Round 2 Status	Round 2	Researcher	Sus and carers	HCP and DMs	Median
Service user involvement in discharge planning (inc. feeling listened to)	87%	4%	7	65%	100%	95%	Include					
Functioning (health, social, etc.)	83%	3%	6	69%	100%	81%	Include					
Mental health and illness (symptom/psychological distress)	83%	3%	6	73%	91%	86%	Include					
Personal Recovery	82%	1%	6	75%	86%	86%	Include					
service user understanding of discharge plan	81%	3%	6	65%	91%	86%	Include					
Quality of life	81%	1%	6.5	65%	90%	86%	Include					
suicide Completed	80%	4%	7	80%	90%	68%	Include					
Readmission	80%	6%	6	77%	77%	86%	Include					
service user involvement in decision making (shared decision making)	77%	4%	7	50%	95%	86%	Include					
service user satisfaction with information provision at discharge (e.g. regarding medication, risk, crisis planning)	77%	6%	6	65%	86%	81%	Include					
service user knowledge of how to access community support (i.e. in an emergency)	77%	3%	6	58%	91%	86%	Include					
Recurrence (i.e. relapse)	75%	1%	6	58%	91%	76%	Include					
Suicide Attempted	75%	4%	6	62%	86%	81%	Include					
discharge to appropriate accommodation	75%	3%	6	69%	91%	67%	Include					
service user satisfaction with treatment	74%	7%	6	58%	91%	71%	Re-present	59%	43%	67%	70%	6
service Availability (e.g. availability of community service, CPN, follow-up, social worker,	74%	7%	6	46%	86%	95%	Re-present	45%	27%	67%	50%	5

bed in residential service)												
Existence of a plan for community engagement/ care post discharge	74%	4%	6	58%	77%	90%	Re-present	72%	57%	83%	90%	6
Feeling of support (from various group i.e. family, professional, friends)	72%	4%	6	65%	77%	71%	Re-present	43%	33%	50%	50%	5
Suicide Risk	71%	3%	6	58%	77%	81%	Re-present	42%	43%	56%	25%	5
Family/carer/other involvement in discharge planning (inc. feeling listened too)	71%	6%	6	65%	77%	75%	Re-present	58%	50%	67%	65%	6
service user readiness for discharge (incl. preparation)	71%	6%	6	58%	77%	81%	Re-present	35%	27%	50%	30%	5
Feeling of safety	70%	7%	6	65%	73%	71%	Re-present	36%	37%	56%	25%	5
service user satisfaction with discharge	69%	4%	6	50%	76%	86%	Re-present	45%	40%	50%	50%	5
Physical Health and Wellbeing	68%	3%	6	50%	77%	81%	Re-present	49%	43%	61%	45%	5
service user experience of discharge	68%	10%	6	46%	86%	76%	Re-present	29%	23%	44%	30%	5
Housing stability	68%	3%	6	62%	77%	67%	Re-present	41%	37%	39%	40%	5
Coping skills	68%	6%	6	50%	77%	80%	Re-present	39%	40%	50%	35%	5
Continuity of contact	68%	6%	6	64%	77%	62%	Re-present	65%	63%	72%	60%	6
Mortality	67%	7%	6	68%	76%	57%	Re-present	42%	43%	50%	30%	5
self management	67%	4%	6	46%	86%	71%	Re-present	29%	23%	39%	20%	5
Length of time before follow up/aftercare	65%	6%	6	46%	77%	76%	Re-present	58%	43%	83%	65%	6
Isolation	65%	9%	6	46%	73%	81%	Re-present	55%	50%	72%	45%	6
Availability of appropriate community support (i.e. allocated key worker)	64%	9%	6	38%	82%	76%	Re-present	52%	27%	72%	70%	6
service user knowledge of own condition	64%	6%	6	42%	91%	62%	Re-present	36%	30%	50%	35%	4
service user expectation of care	63%	3%	6	35%	76%	81%	Re-present	28%	20%	28%	40%	4
Hopelessness	63%	9%	6	54%	67%	71%	Re-present	46%	47%	44%	45%	5
suicidal Ideation	62%	7%	6	46%	77%	67%	Re-present	51%	50%	61%	45%	6

Medication Adherence	62%	4%	6	50%	82%	52%	Re-present	45%	47%	44%	40%	5
Loneliness	62%	10%	6	42%	68%	81%	Re-present	41%	37%	56%	30%	5
Information about the discharge reported to other relevant service	62%	9%	6	50%	73%	71%	Re-present	48%	37%	78%	40%	5
service user experience of coercion at discharge	61%	6%	6	46%	77%	63%	Re-present	43%	40%	67%	35%	5
Clinical readiness for discharge	60%	6%	6	48%	67%	67%	Re-present	36%	30%	39%	40%	5
Medication side Effects	59%	7%	6	38%	77%	67%	Re-present	41%	30%	61%	35%	5
Emergency department visit post discharge	59%	6%	6	58%	68%	52%	Re-present	62%	63%	67%	60%	6
Quality of interpersonal relationship (friend and family)	59%	7%	6	50%	77%	52%	Re-present	46%	40%	72%	35%	5
Iatrogenic harm (patient safety incident or harm caused by the care system)	59%	4%	6	40%	64%	71%	Re-present	51%	37%	72%	50%	6
Completion of planned care	59%	7%	6	50%	76%	52%	Re-present	39%	40%	56%	30%	5
Concern/anxiety about discharge	58%	9%	6	31%	73%	76%	Re-present	26%	17%	33%	35%	4
Autonomy (e.g. independence, autonomous decision making)	57%	3%	6	35%	73%	70%	Re-present	36%	27%	50%	30%	5
staff understanding of safe and effective discharge	57%	10%	6	40%	68%	67%	Re-present	41%	30%	67%	35%	4
self injury (i.e. harm to self)	57%	6%	6	50%	64%	52%	Re-present	49%	53%	61%	35%	5
Financial support (e.g. benefit advice, debt advice)	57%	9%	6	38%	82%	57%	Re-present	46%	40%	72%	30%	5
stable relationship with health professional	55%	4%	6	38%	71%	58%	Re-present	33%	37%	50%	20%	5
Medication Management	54%	3%	6	42%	71%	52%	Re-present	42%	37%	61%	40%	5
Carer/family/other satisfaction with discharge	54%	9%	6	35%	68%	57%	Re-present	36%	27%	61%	35%	4
Clinical Recovery	53%	7%	6	60%	55%	43%	Re-present	36%	33%	50%	30%	5
service use	52%	4%	6	46%	50%	63%	Re-present	30%	33%	39%	15%	4
Violence	52%	9%	6	46%	60%	48%	Re-present	29%	33%	33%	15%	4
Victim of crime	52%	12%	6	38%	68%	48%	Re-present	28%	30%	44%	10%	4
Experience of stigma	52%	7%	6	42%	59%	57%	Re-present	30%	20%	50%	25%	4

For peer review only

Engagement with psychological intervention	51%	4%	6	35%	45%	76%	Re-present	33%	30%	67%	5%	4
Cancellation or change to follow up meeting	51%	12%	6	19%	82%	57%	Re-present	42%	23%	67%	40%	5
serious incident (i.e. serious incident reported formally)	50%	6%	5.5	44%	45%	62%	Exclude					
Employment	50%	7%	5.5	58%	48%	38%	Exclude					
Medication Knowledge	49%	9%	5	35%	64%	52%	Exclude					
Engagement with community service	49%	4%	5	35%	50%	71%	Exclude					
Length of stay pre-discharge	46%	10%	5	38%	55%	48%	Exclude					
Family/carer/other readiness for discharge	46%	9%	5	42%	55%	43%	Exclude					
Leaving the hospital against medical advice	45%	14%	5	46%	55%	33%	Exclude					
Outpatient appointment/visit adherence	44%	4%	5	54%	57%	24%	Exclude					
Aggression	44%	10%	5	42%	43%	48%	Exclude					
Delay in expected discharge	43%	13%	5	12%	67%	57%	Exclude					
substance Misuse (incl. alcohol)	41%	9%	5	28%	50%	48%	Exclude					
serious accidental injury	40%	12%	5	25%	36%	62%	Exclude					
Community participation	39%	6%	5	35%	36%	48%	Exclude					
stability	38%	4%	5	13%	59%	47%	Exclude					
Advocate involvement in discharge planning	37%	12%	5	24%	45%	45%	Exclude					
Professional/care team satisfaction with information provision at discharge	36%	13%	5	19%	45%	48%	Exclude					
Primary Care/Community service/Nongovernmental or Charity service satisfaction	35%	16%	5	15%	50%	48%	Exclude					
Police intervention post discharge	34%	16%	5	35%	32%	35%	Exclude					
staff satisfaction with discharge	28%	16%	5	19%	36%	29%	Exclude					
Boredom	26%	25%	4	12%	36%	33%	Exclude					
Meaningful Activity (i.e. employment, studying, volunteering)	New outcome proposed in round 1						Include	77%	73%	83.33%	80%	6
Resilience	New outcome proposed in round 1				Do not present							
Dual harm (i.e. a person harms themselves and others)	New outcome proposed in round 1						Exclude	32%	23%	55.56%	30%	4

For peer review only

Annual admissions	New outcome proposed in round 1	Do not present							
Harm to others (violence, criminal or risk-taking behaviour)	New outcome proposed in round 1	Do not present							
Levels of patient confidentiality	New outcome proposed in round 1	Do not present							
Personal learning	New outcome proposed in round 1	Do not present							

For peer review only

File 5: Proposed potential service user discharge experience PROM that includes items of most importance to stakeholders in addition to the core outcome set

Item	Strongly Agree	Agree	Neither Agree nor Disagree	Disagree	Strongly Disagree
1. I felt involved in my discharge planning					
2. I understood my discharge plan					
3. I know how to access support in the community					
4. I was frequently involved in decisions about my care at discharge					
5. I was discharged to appropriate accommodation					
6. I was satisfied with the information provided to me at discharge					

File 6: Outcome Measure Recommendation Results

Methods

After the core outcome set was agreed in the consensus meeting, we invited all participants from the earlier stages of the project to recommend measures and time markers in a final online questionnaire. Participants were invited to participate if they had been involved in any of the previous online rounds. The invitation made it clear that the questionnaire is most relevant to researchers, but that other groups with an opinion or interest are welcome to contribute. This was due to the specific knowledge of instruments required to complete this round.

In this questionnaire participants were presented with the four core outcomes. For each core outcome they were presented with any measures used to assess that outcome in our systematic review studies [1] and any additional measures that had been recommended to the team during the process. Participants were asked to choose the one most appropriate, (don't know, other, new instrument, no instrument were also options). A second question also asked which time markers would be recommended, with options to select all applicable. These options were also developed based on time markers used in the systematic review [1].

Results

Forty-three of the 93 invited participants responded (15 service users, 8 family members/carers, 23 researchers, 10 healthcare professionals, 3 end users of research), although as in previous rounds these were not distinct categories. Fifty-three percent of the respondents were researchers, this was expected as in the email we suggested that this stage may be more meaningful or of interest to this group, but as a team we chose not to exclude other groups with opinions on measurement instruments. Twenty-three percent of participants were international

For peer review only

researchers (from USA, Switzerland, Canada, and Australia). Table 4 shows the preliminary minimum measure recommendations and time markers, additional file 5 shows the results upon which the recommendations were based.

Table 1: Summary of Measurement Recommendations

Core Outcome	Instrument/Measure	Time Marker
Readmission	Retrospective review of administrative data	Within 28 days of discharge
Suicide Completed	Retrospective review of administrative data	Within 28 days of discharge
Psychological Distress	Kessler Psychological Distress (K10)	One month post-discharge
Quality of Life	ReQoL-10	One month post-discharge

1. Readmission

A minimum recommendation of using retrospective review of administrative data for readmissions within a defined time period, the most agreed was 28 days. Participants indicated that routine data collection might cover slightly different time periods. Twenty-six of the 43 participants recommended a measure of around a month (1 month, 30 days or 28 days) with 28 days being this most popular. However, they also advise that this should be supplemented either by cross-checking with service-users, case managers or carers, where possible to improve quality of data. Those looking for more comprehensive data may also like to record 7 days, 3 months and 6 months as these were also popular recommendations.

2. Quality of Life

The participants recommended that researchers use the Recovering Quality of Life (ReQoL-10) at one month post-discharge [35]. This was the most recommended instrument by the group. However, many participants also voted for ReQoL 20, a large proportion of the group suggested this outcome. As this is a quality of life measure specific to mental health recovery they felt this is most appropriate. The one month time marker is in-keeping with the other COS time frames, making a more comprehensive and accessible core outcome set. Those using within-participant measures of quality of life may like to also measure a pre-discharge baseline. Researchers looking for more thorough assessment of quality of life may like to also measure at 7 days post-discharge and 3 months, as these were also highly recommended time markers or use the ReQoL 20 and report both scores for comparability.

3. Suicide Completed

The participants recommended retrospective review of administrative data, for suicide completed within 28 days of discharge. Retrospective review is in line with other outcomes and was marginally the highest suggestion. We chose within 28 days for consistency with readmission data. Researchers looking for more comprehensive data may want to use 7 days and 3 months as these were highly recommended also. They may also want to cross-check this information against other sources (carers/case managers) to ensure it is correct and

For peer review only

reported, particularly as participants mentioned the impact of incorrect coroner's reports on such data.

4. Psychological Distress

The participants recommended Kessler Psychological Distress (K10) one month post-discharge [36] . For consistency with other outcomes we recommend measure at one month. Seven days and 3 months are also highly recommended, so we would recommend these for research that is more robust. Although there were very few votes for instruments for psychological distress and qualitative comments revealed that participants felt this is not measurable. The same amount of people who voted for K10 also voted for interviews or other measures and a similar amount recommended the development of a new measure, CORE-10 was similarly close [37]. Whilst we make this recommendation, we also suggest that future researchers may look to develop something specific for Psychological Distress in this core outcome set. Interviews would not effectively facilitate the between study comparison, the key purpose of a COS.

Outcome 1: Readmission

Measure	Number of votes	Important comments
Interviews with SUs	12	• In some countries… there is no easily accessible data on readmission rates…in our experience self-reported in the most reliable way
Retrospective review of administrative data	13	• Might not show people who need admission but don't because there's no bed
Extracted from case-managers notes and cross-checked with hospital records	10	• Might be easier to gather administrative data, but worth cross-checking to improve quality of information
Self-reported questionnaire	1	
Other- carer interview	2	
Total	38	

Conclusion: A minimum recommendation of using retrospective review of administrative data. This will allow for various studies with diverse time and financial limits to use the COS. However, we also advise that this should supplemented either by cross-checking with service-users, case managers or carers, where possible to improve quality of data.

Time Markers

Time marker	Number of Votes
Within 2 days	3
Within 3 days	2
Within 7 days	17
Within 28 days	12
Within 30 days	7
Within 1 month	7
Within 6 weeks	4
Within 12 weeks	7
Within 3 months	11
Within 6 months	14
Other (1yr and 3yrs)	1

For peer review only

Around 3 days	5
Around a month	26
Around 3 months	18

Conclusion: The minimum recommendation to record readmission within 28 days. 26 of the 43 participants recommended a measure of around a month (1 month, 30 days or 28 days) with 28 being this most popular. Those looking for higher quality or more comprehensive data may also like to record 7 days, 3 months and 6 months as these were also popular recommendations.

Outcome 2: Quality of Life

Instrument	**Number of votes**	**Comments**
ReQol- 10	9	'I think ReQol would be best for patients in MH services as I understand it was validated for CMHT patients but if someone didn't have contact with MH services before and isn't under a CMHT afterwards another measure might be better.'
ReQol- 20	3	
Quality of Life Brief Version (Lehman)	2	
WHO Quality of Life Scale	5	
Manchester Short Assessment Quality of Life (MANSA)	1	
Satisfaction with Life Scale (SWLS)	1	
SF12	2	
EQ5D	0	
AQoL-8D	0	
Other (ICECAP)	1	
Develop a new tool	2	
No instrument (interviews etc. instead)	7	I think the use of tools should be complemented with interviews with service users and carers.
Total	33	
ReQoL combined (10+20)	12	

Conclusion: We recommend that researchers use ReQoL-10. This was the most voted for instrument. If we also combine the scores with those who voted for ReQoL 20, a large proportion of the group suggested this outcome. As this is a quality of life measure specific to mental health recovery we feel this is most appropriate.

Time markers

**Time Point**	**Number of votes**
Within 2 days	3
Within 3 days	2
Within 7 days	16
Within 28 days	11
Within 30 days	6
Within 1 month	9
Within 6 weeks	3
Within 12 weeks	5
Within 3 months	21
Within 6 months	11
Other (within 9 months, 12 months and 3 years)	2
Pre-discharge	9
Around 3 days	5
Around 1 month	26
Around 3 months	26

Conclusion: We recommend a minimum measure of QoL at one month post-discharge in RCTs. This is in keeping with the readmission time frames, making a more comprehensive and accessible core outcome set. Those using within-participant measures of quality of life may like to

For peer review only

also measure a pre-discharge baseline. Those looking for more thorough assessment of QoL may like to also measure at 7 days post-discharge and 3 months, as these were also highly recommended time markers.

Core Outcome 3: Suicide completed

Measure	Number of votes
Retrospective review of administrative data	18
Extracted from case managers and cross-checked with hospital records	17
Extracted from clinical case notes	14
Other<br>Extracted from serious incident reporting<br>From family and friends	4
Total	53

Conclusion: Retrospective review of administrative data. To keep in line with other outcomes and was marginally highest measure.

Time Marker	Number of votes
Within 2 days	2
Within 3 days	2
Within 7 days	20
Within 28 days	12
Within 30 days	4
Within 1 month	9
Within 6 weeks	0
Within 12 weeks	4
Within 3 months	14
Within 6 months	13
Other	2
Around 3 days	4
Around 1 month	25
Around 3 months	18

Conclusion. Within 28 days for consistency with other outcomes. Other researchers may want to use 7 days and 3 months as these were highly recommended also.

Outcome 4: Psychological Distress

Instrument	Number of votes
Kessler Psychological Distress (K10)	6
CORE-10	5
Brief Psychiatric Rating Scale (BPRS)	3
Brief Symptom Inventory (BSI)	1
The positive and negative symptom scale	1
The Discharge List (DL) â€“ 20 item scale	2
Clinical Global Impression to Assess illness severity	0
ASI (Psychiatric problem subscale)	0
PSYRAT (Psychological Distress subscale)	1
Develop a new self-assessment tool for psychological distress	5
Other	1
No instrument (interview or other method instead)	6
Total	31

Conclusion: We recommend Kessler Psychological Distress (K10). Although there were very few votes for measures. The same amount of people who voted for K10 also voted for interviews or other measures and a similar amount recommended the development of a new measure,

For peer review only

CORE-10 was similarly close. Whilst we make this recommendation, we also suggest that future researchers may look to develop something specific for this core outcome set. Interviews would not allow for easy comparison of scores so would not be relevant for a core outcome set.

Time Markers

Time Marker	Number of votes
Within 2 days	2
Within 3 days	4
Within 7 days	22
Within 28 days	8
Within 30 days	4
Within 1 month	14
Within 6 weeks	3
Within 12 weeks	4
Within 3 months	15
Within 6 months	14
Other	3
Pre-discharge baseline measure at ...	5
Around 3 days	6
Around 1 month	26
Around 3 months	19

Conclusion: The minimum recommendation is one month post-discharge. For consistency with other outcomes we recommend measure at one month. 7 days and 3 months are also highly recommended, so we would recommend these for more thorough research.

For peer review only

For peer review only

For peer review only

SECTION/TOPIC	ITEM No.	CHECKLIST ITEM	PAGE NO.
**TITLE/ABSTRACT**			
Title	1a	Identify in the title that the paper reports the development of a COS	1
Abstract	1b	Provide a structured summary	2
**INTRODUCTION**			
Background and Objectives	2a	Describe the background and explain the rationale for developing the COS.	3-4
	2b	Describe the specific objectives with reference to developing a COS.	4
Scope	3a	Describe the health condition(s) and population(s) covered by the COS.	4
	3b	Describe the intervention(s) covered by the COS.	4
	3c	Describe the setting(s) in which the COS is to be applied.	4
**METHODS**			
Protocol/Registry Entry	4	Indicate where the COS development protocol can be accessed, if available, and/or the study registration details.	4
Participants	5	Describe the rationale for stakeholder groups involved in the COS development process, eligibility criteria for participants from each group, and a description of how the individuals involved were identified.	5
Information Sources	6a	Describe the information sources used to identify an initial list of outcomes.	5
	6b	Describe how outcomes were dropped/combined, with reasons (if applicable).	6
Consensus Process	7	Describe how the consensus process was undertaken.	6-8
Outcome Scoring	8	Describe how outcomes were scored and how scores were summarised.	6-8
Consensus Definition	9a	Describe the consensus definition.	7-8
	9b	Describe the procedure for determining how outcomes were included or excluded from consideration during the consensus process.	7-8
Ethics and Consent	10	Provide a statement regarding the ethics and consent issues for the study.	8
**RESULTS**			
Protocol Deviations	11	Describe any changes from the protocol (if applicable), with reasons, and describe what impact these changes have on the results.	N/A
Participants	12	Present data on the number and relevant characteristics of the people involved at all stages of COS development.	8-9
Outcomes	13a	List all outcomes considered at the start of the consensus process.	SF4
	13b	Describe any new outcomes introduced and any outcomes dropped, with reasons, during the consensus process.	SF2&4
COS	14	List the outcomes in the final COS.	12
**DISCUSSION**			
Limitations	15	Discuss any limitations in the COS development process.	15
Conclusions	16	Provide an interpretation of the final COS in the context of other evidence, and implications for future research.	15
**OTHER INFORMATION**			
Funding	17	Describe sources of funding/role of funders.	17
Conflicts of Interest	18	Describe any conflicts of interest within the study team and how these were managed.	16

doi:10.1371/journal.pmed.1002148.t001
